# Silent struggles: Assessing physical and psychosocial burdens among caregivers of children with sickle cell disease in western Sudan–A cross-sectional study

**DOI:** 10.1371/journal.pone.0336469

**Published:** 2025-11-25

**Authors:** Weaam Abdallah, Wisal Abbas, Swsan A. M. Elsharif, Maaza Hamid Ahmed Ibrahim, Walaa Abdalla, Fadwa Saad

**Affiliations:** 1 Faculty of Medicine, University of Khartoum, Khartoum, Sudan; 2 Department of Physiology, Faculty of Medicine, Kordofan University, Elobeid, North Kordofan, Sudan; 3 Faculty of Medicine, Kordofan University, Elobeid, North Kordofan, Sudan,; 4 Medicine Program, Sheikan College, Elobeid, North Kordofan, Sudan; 5 Department of Community Medicine, Faculty of Medicine, University of Khartoum, Khartoum, Sudan; University of Ghana College of Health Sciences, GHANA

## Abstract

Sickle cell disease (SCD) is a major cause of morbidity and mortality. It poses a major health problem in Sudan, especially in the western region, where the prevalence of the disease is highest. SCD places a substantial psychosocial and economic burden on the caregivers. Given that there is no readily available effective treatment, caregiving for children with SCD is highly demanding and is associated with inescapable stress. This observational, cross-sectional facility-based study aimed to assess the caregivers’ physical and psychosocial burdens. A total of 123 caregivers who attended the Sudan Sickle Cell Anaemia Centre, El Obeid, western Sudan, were interviewed using the abridged Arabic Zarit Burden Interview Scale (ZBI-A) between March 15 and April 12, 2023. Data was then analysed using SPSS v.20 and summarised into medians and interquartile ranges. A p-value of <0.05 was considered statistically significant. The Mann-Whitney U test and Kruskal-Wallis tests were used to identify the characteristics of caregivers and their SCD children that were associated with the total Zarit burden score of the caregivers. (84.6%) of caregivers were biological mothers, (41.5%) were (20–30) years old, and (39.0%) were from outside Elobeid. Most mothers (37.4%) and fathers (39.8%) only completed primary schooling; thus, most mothers (84.6%) were housewives, and most fathers (77.2%) were free workers. (82.9%) had a family monthly income level of <100,000 SDG (<167 USD). (46.8%), (35.5%), and (17.7%) experienced mild, moderate, and severe levels of caregiving burden, respectively. Total Zarit burden scores of the caregivers were significantly associated with residing outside Elobeid (p = 0.028), lower maternal and paternal educational levels (p = 0.008) (p = 0.036), respectively, lower paternal employment status (p = 0.034) and those whose children with SCD were aged 5–9 years (p = 0.008). In conclusion, (46.8%), (35.5%) and (17.7%) of the participants experienced mild, moderate, and severe levels of caregiving burden, respectively.

## Introduction

Sickle Cell Disease (SCD) is a chronic blood disorder that starts manifesting in the first six months of life [1]. The first description of SCD was made in the United States by Dr. James Herrick at the beginning of the 20th century. He described the disease as “peculiar, elongated, and sickle-shaped red blood corpuscles in a case of severe anaemia” [2]. Sickling of the red blood cells gives rise to the two main clinical manifestations of the disease: hemolytic anemia [3,4] and vasocclusion, which follows the sticking of the sickled red cells in the small blood vessels. Vasocclusion, in turn, leads to reduced blood flow and tissue necrosis, which is followed by episodes of severe pain termed “sickle cell crisis” [5]. These sickle cell crises are the leading causes of distress among SCD patients, accounting for nearly 90% of SCD-related hospital admissions [6]. They are characterised by being unpredictable and are usually variable in severity, duration, and location [7].

It is estimated that each year, 300,000 are born with SCD, with 70% of them in Africa alone [8]. Death rates of children in Africa reach up to 90%. These extremely high mortality rates in African SCD patients are the result of cultural background, a paucity of public awareness of the disease, and a scarcity of healthcare centres, accounting for limited early detection of the disease, and thus it remains undiagnosed and untreated. The recent introduction of simple and comprehensive SCD programs in sub-Saharan African countries has significantly reduced mortality rates [9]. This disease was first reported in Sudan by Archibald in 1926 [10]. It poses a major health problem in the country, especially in the western region, where the prevalence of the disease is the highest (30.4%) [11], especially in the Messeriya tribe, a branch of the Bagara tribe, in which it is estimated to be 18.2% in Kordofan and 30.4% in Darfur [12].

SCD places a significant psychosocial and economic burden on the children’s caregivers. This burden can extend to involve the whole community, especially in LMICs like Sudan. Generally, the caregiving burden consists of two components: subjective and objective burden. Objective burden includes measurable effects such as disrupted interrelationships, economic hardships, loss of jobs, and social and leisure activities. In contrast, subjective burden is demonstrated by the psychological sufferings experienced by the caregivers, like depression, feelings of shame, and embarrassment. Caregiving for children with serious chronic medical conditions in general is highly demanding and is associated with pervasive stresses correlating with poor physical and psychological health outcomes [13–16]. It is worth noting that a high percentage of families with SCD-affected children face difficulties with transportation and in accessing medical centres, resulting in less than 70% of them receiving the appropriate medical care. Furthermore, sickle cell centres are few and are usually centralised in urban cities, rendering it difficult for those living in distant, rural residences to seek adequate medical care for their SCD children [17]. Psychosocial distress perceived by the caregivers is associated with lower psychological health of their SCD children. Moreover, it has a direct negative impact on the SCD-related health outcomes of their children, as evidenced by worse perceived sickle cell pain, poorer child health outcomes, and increased use of health care. Previous studies associated poorer caregiver psychological health with poorer treatment adherence for their SCD children [18,19].

Despite the high prevalence of SCD in western Sudan, the studies on the burden on the caregivers of children with SCD are limited, and this issue has rarely been considered in previous studies. This study, therefore, aims to assess the physical and psychosocial burdens experienced by caregivers of children with SCD in Elobeid, western Sudan and to assess the association between the sociodemographic characteristics of the caregivers and the level of burdens perceived by them. The results of this study could be used as a resource to develop strategies to reduce the impact of SCD on families in Sudan.

## Materials and methods

### Study design

This study utilised an observational, descriptive, cross-sectional, facility-based design. This study design was used as it was feasible and suitable for both the context and the participants. It provides timely identification of the current burden levels and possible sociodemographic risk factors. It also allows comparability with other similar studies done in LMICs [6,13,16].

### Study setting

The study was conducted at the Sudan Sickle Cell Anaemia Centre (SSCAC), located in El Obeid Specialised Pediatric Hospital, El Obeid, North Kordofan, Sudan, from March 15, 2023, to April 12, 2023. The SSCAC is a national, voluntary, non-governmental organisation licensed by the Federal Humanitarian Aid Commission. It was established in Elobeid in 2012, which started as a sickle cell clinic led by a senior paediatrician, but then evolved gradually to include training packages, genetic counselling, and laboratory investigations. The choice of Elobeid for the SSCAC is based on its representation of the Kordofan region, an area with a documented high prevalence of SCD in Sudan [20].

### Study population

The study targeted caregivers of children diagnosed with SCD who attended the SSCAC at El Obeid Specialised Pediatric Hospital during the study period. The study included caregivers of SCD children who were younger than 18 years of age and who lived with their SCD children in the same house. The study excluded caregivers of children older than 18 years, patients acting as their own caregivers, and caregivers with a diagnosed psychiatric illness.

### Sample size

To measure the sample size for this study, the Cochran’s formula for sample size estimation was used [21].


$$n=Z^{\text{2}}*p*\frac{\left(\text{1}-p\right)}{e^{\text{2}}}$$

Where:

n = Required sample size

z = Z-score corresponding to the desired confidence level (1.96 corresponds to 95% confidence level)

p = Estimated prevalence (14.8%) [22].

e = Margin of error (here is 5%)

The calculated sample size was 194 participants. Based on the clinic’s flow rate, the study period was to be held for 8 weeks to cover the target. However, due to the onset of the war in Sudan on April 15, 2023, data collection was disrupted, and the SSCAC was shut down. Consequently, only 123 caregivers were included in the study.

### Sampling technique

The study used a convenience sampling technique. All eligible caregivers attending the SSCAC during the study period were included in the study.

### Data collection

Data collection took place weekly on Wednesdays from 8:00 AM to 2:00 PM. Data was then collected from 124 caregivers during their waiting time at the SSCAC. Caregivers were interviewed using a semi-structured questionnaire and the Arabic abridged Zarit Burden Interview Scale (ZBI-A). The researcher conducted individual interviews in a comfortable and private setting. Verbal consent was obtained after explaining the study’s purpose and assuring confidentiality. Each interview lasted approximately 15 minutes without interrupting clinic operations. One caregiver was excluded from the study for having a child older than 18 years old.

### Tools and instruments

A semi-structured questionnaire was developed by the researcher to gather socio-demographic information about the caregivers and their SCD children, including age, marital status, residence, tribe, educational level, employment status, and monthly income. The abridged Arabic version of the Zarit Burden Interview Scale (ZBI-A) was used to measure caregivers’ burden levels. It was validated in 2013, with a Cronbach’s alpha value of 0.77 [23]. This scale is an adapted Arabic version of the original Bedard’s short version ZBI-12. This scale consists of 12 items rated on a 5-point Likert scale (0–4), with higher scores indicating a greater burden. The total score ranges from 0 to 48, classified as follows: 0–10: No to mild burden, 10–20: Mild to moderate burden, and > 20: High burden [24]. A pilot study, which was conducted at the start of the study with 12 participants, confirmed the comprehensibility of the (ZBI-A), which was in standard Arabic, with the participants who spoke in the local Sudanese dialect. Data from the pilot study were excluded from the final analysis to avoid contamination.

### Statistical analysis

Data was collected and entered into Excel 2022. Then it was cleaned, revised, coded, and imported into the Statistical Package for the Social Sciences (SPSS) version 20. Data was skewed, so descriptive data was summarised into medians and interquartile ranges to show the magnitude of differences. Non-parametric tests, the Mann-Whitney U test (for two groups) and Kruskal-Wallis’s test (for three or more groups), were used to identify the characteristics of the caregivers and their SCD children that were associated with the total Zarit burden score of the caregivers. A p-value of <0.05 was considered statistically significant.

### Ethical considerations

Ethical approval for this study was obtained from the Department of Community Medicine, Faculty of Medicine, University of Khartoum, approval number: [COMMED 2023-95-17]. All participants were informed about the purpose of the study clearly without any enhancement or convincing to participate, and verbal informed consent was obtained prior to data collection. The consent process was witnessed and documented by attending medical staff who were not directly involved in the research.

## Results

### Participants’ sociodemographic characteristics

A total of 123 caregivers were included in this study. Most of the participants were females (89.4%), and the majority (77.3%) belonged to the 20–40 years age group. Most of the participants were married (91.1%), and the remainder (8.9g%) were either single, widowed, or divorced. Regarding the caregivers’ relationship to their SCD children, the majority (84.6%) were biological mothers, and (10.6%) were fathers. More than two-thirds of the participants (72.4%) lived in urban areas, while (39.0%) resided outside Elobeid. More than half of the participants (54.5%) had more than three children in the family, and nearly one-third (35.8%) had two or three children. Most of them had a single child with SCD (82.1%), (16.3%) had two children with SCD, and (1.6%) had three or more children with SCD. The majority (92.7%) of the participants had a family monthly income level of less than 200,000 SDG (less than 1.85 USD per day per head), which is below the international poverty line of fewer than 2.15 USD per day per head [25]. There was a significant difference in the total burden levels across the area in which the caregiver resided, with people residing outside Elobeid recording a higher median score (Md (IQR)) = (16(8–25)), than people residing in Elobeid (Md (IQR)) = (10(8–17)), (p-value <0.05). (Table 1) presents the condensed data on baseline characteristics of participants; for a fully detailed table, refer to S1 Table.

**Table 1:** Baseline characteristics of caregivers of children with SCD participating in the study, SSCAC, Elobeid, North Kordofan, Sudan, 2023, N: 123.

Variable	N	%	Total Zarit	
			Median (IQR)	P value
**Age**				0.092^a^
<20 Years	2	1.60%	28 (16-40)	
20-30 Years	51	41.5%	10 (8–18)	
31-40 Years	44	35.8%	10 (8-17)	
>40 Years	26	21.1%	15 (10 –20 )	
**Relationship to child**				0.909^a^
Mother	104	84.6%	12 (8-20)	
Father	13	10.6%	13 (9-16)	
Sibling	1	0.80%	16 (16-16)	
Aunt	3	2.40%	17 (8-18)	
Grandparent	2	1.60%	9 (8-10)	
**Gender of the caregiver**				0.862^b^
Male	13	10.6%	13 (9-16)	
Female	110	89.4%	12 (8-19)	
**Area**				**0.028** ^**b**^
Elobeid	75	61.0%	10 (8-17)	
Outside Elobeid	48	39.0%	16 (8-25)	

p-value: significant level at 95% confidence interval, IQR: interquartile range.

^a^Kruskal-Wallis’s test, ^b^Mann-Whitney U test.

*At the time of the study, 1 USD = 600 SDG.

Regarding mothers’ education, (37.4%) had primary education, and (18.7%) had university degrees. Similarly, (39.8%) of fathers completed primary education, but only (13.8%) had a university degree or higher. In terms of occupation, most (84.6%) mothers were housewives, and the majority (77.2%) of fathers were free workers. The difference in burden scale for parents’ characteristics was statistically significant for mothers’ education, fathers’ education, and fathers’ occupation (p-value <0.05). Illiterate mothers (Md(IQR)= 21(16–28)), and mothers who went to Khalwa (Md(IQR)= 18(14–26)) recorded higher median scores of total Zarit burden scale, than mothers with secondary education (Md(IQR)= 10(6-14)), and university degree (Md(IQR)= 11(8–18)), (p-value: 0.008). Similarly, illiterate fathers recorded the highest median scores of the total Zarit burden scale (Md(IQR)= 20(17–24)) among other education sub-categories (p-value: 0.036). In terms of occupation, the highest median scores of the total Zarit burden scale are recorded among unemployed fathers (Md(IQR)= 20(18–21)) (p-value: 0.034) (Table 2).

**Table 2:** Characteristics of parents of children with SCD participating in the study, SSCAC, Elobeid, North Kordofan, Sudan, 2023, N: 123.

Variable	N	%	Total Zarit	
			Median (IQR)	P value
**Mothers’ education**				**0.008** ^a^
Illiteracy	9	7.30%	21 (16–28)	
Khalwa	12	9.80%	18 (14–26)	
Primary education	46	37.4%	11 (8–20)	
Secondary education	33	26.8%	10 (6–14)	
University	23	18.7%	11 (8–18)	
**Fathers’ education**				**0.036** ^a^
Illiteracy	13	10.6%	20 (17-24)	
Khalwa	10	8.10%	10 (7-18)	
Primary education	49	39.8%	11 (8-19)	
Secondary education	34	27.6%	10 (8-15)	
University	15	12.2%	10 (6-18)	
Higher degrees	2	1.60%	10 (8-12)	
**Mother Occupation**				0.270^a^
Employee	12	9.80%	14 (9-21)	
Housewife	104	84.6%	11 (8-18)	
Daily worker	7	5.70%	20 (10-26)	
**Father Occupation**				**0.034** ^a^
Employee	23	18.7%	8 (8-12)	
Free work	95	77.2%	12 (8-20)	
Unemployed	5	4.10%	20 (18-21)	

p-value: significant level at 95% confidence interval, IQR: interquartile range.

^a^Kruskal-Wallis’s test.

**Fig 1:**
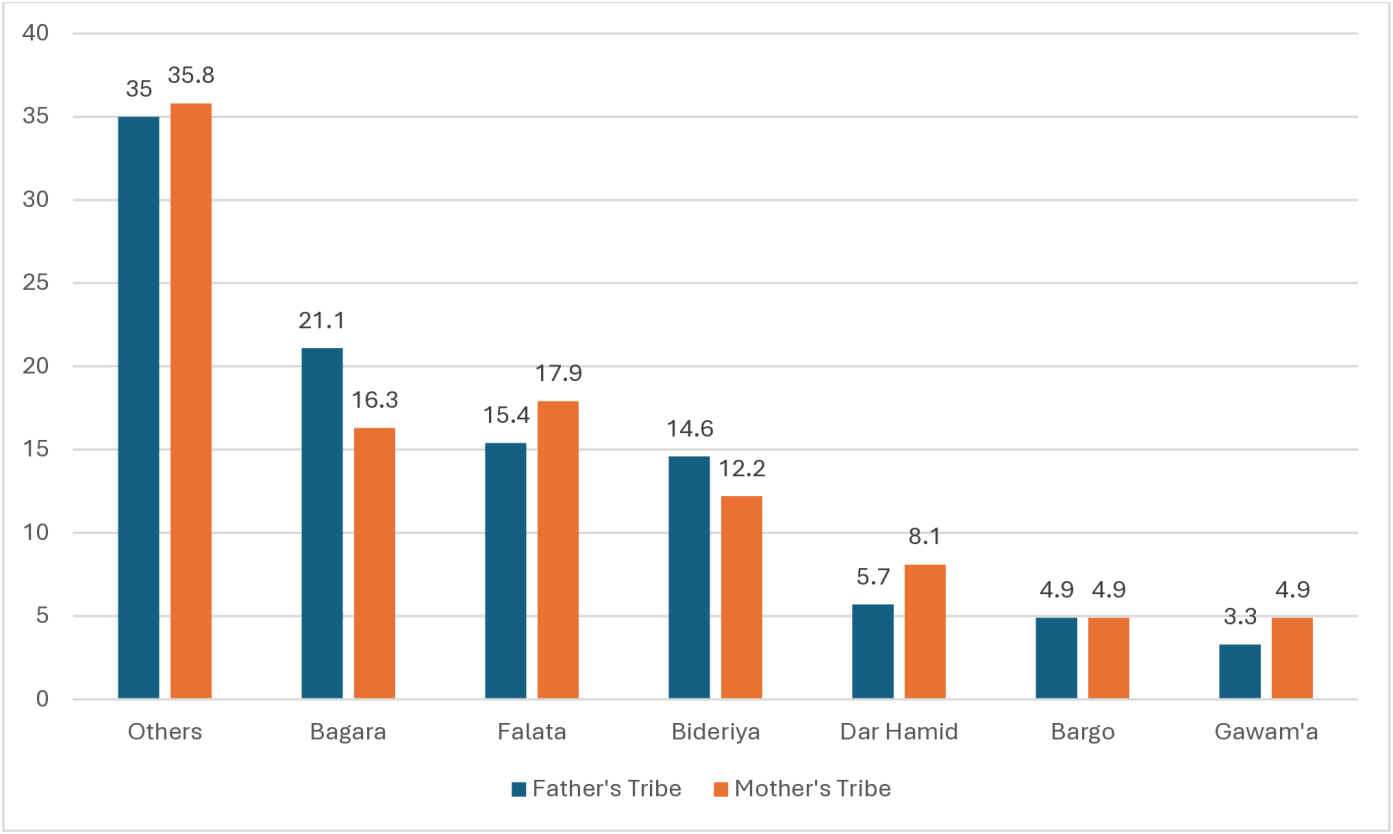
Tribal affiliation of parents of children with SCD participating in the study, SSCAC, Elobeid, North Kordofan, Sudan, 2023, N: 123.

The most prevalent mothers’ tribes were Bagara (16.3%), Bideriya (12.2%), and Falata (17.9%). Most fathers also belonged to the same tribes, with (21.1%), (14.6%), and (15.4%) from the Bagara, Bideriya, and Falata tribes, respectively, as shown in Fig 1.

### Characteristics of the children with SCD

(37.4%) of children were in the age group of (0–4 years), and (32.5%) of children were in the age group of (5–9) years. (22.0%) of children were diagnosed with SCD at younger than 6 months of age, more than half (52.8%) of them were diagnosed at age (6–12 months), and (52.8%) of them were diagnosed at older than 12 months. The majority of children regularly take hydroxyurea (86.2%) and folic acid (97.6%). Nearly three-quarters (71.5%) of children had health insurance. Regarding school attendance, (68.3%) of children reported that they irregularly or never went to school, with (24.4%) of them stating that the cause was their illness. Significant statistical difference in the burden scale was found across child age groups and the reasons for irregular school attendance (p-value <0.05). The highest median score of the total Zarit burden scale was recorded among participants, whose sick child’s age ranged from five to nine years (Md(IQR)= 16(10–25)), (p-value: 0.008). Participants who reported school attendance irregularity due to financial reasons (Md(IQR)= 26(20–32)), and due to illness (Md(IQR)= 17(12–22)) recorded higher median score than due to young age (Md(IQR)= 9(6–16)) (p-value: 0.008). (Table 3) presents the condensed data on the characteristics of children with SCD participating in the study. For the fully detailed table, refer to S2 Table.

**Table 3:** Characteristics of children with SCD participating in the study, SSCAC, Elobeid, North Kordofan, Sudan, 2023, N: 123.

Variable	N	%	Total Zarit	
			Median (IQR)	P value
**Child gender**				0.635^b^
Male	64	52.0%	12 (8-20)	
Female	59	48.0%	10 (8-17)	
**Child age**				**0.008** ^a^
0-4 Years	46	37.4%	9 (6-16)	
5-9 Years	40	32.5%	16 (10-25)	
10-13 Years	29	23.6%	10 (8-18)	
14-18 Years	8	6.50%	9 (8-15)	
**Diagnosis age**				0.430^a^
<6 months	27	22.0%	14 (8-25)	
6-12 months	65	52.8%	11 (8-17)	
>12 months	31	25.2%	10 (6-20)	
**School attendance**				0.293^a^
Regularly	40	32.5%	10 (8-17)	
Irregularly	13	10.6%	14 (12-20)	
Never	70	56.9%	12 (8-20)	
**If irregularly or never to the previous question, why?**				**0.003** ^a^
Below school age (<5 years)	51	61.4%	9 (6-16)	
Illness	30	36.1%	17 (12-22)	
Financial	2	2.40%	26 (20-32)	

p-value: significant level at 95% confidence interval, IQR: interquartile range.

^a^Kruskal-Wallis’s test, ^b^Mann-Whitney U test.

### Total burden scores of the caregivers of children with SCD

**Fig 2:**
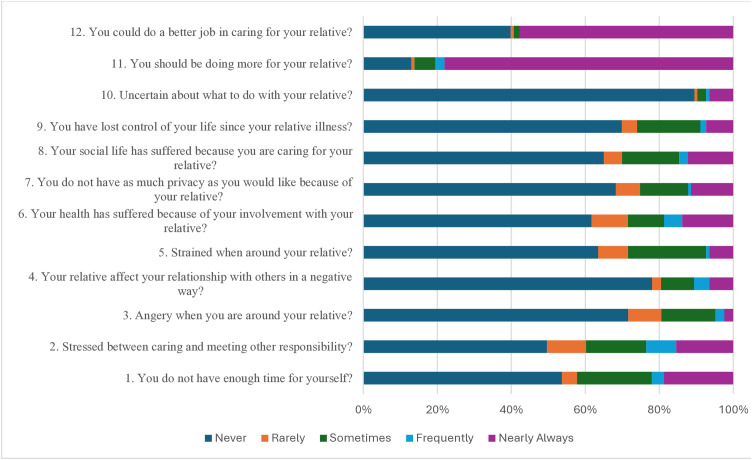
Distribution of Zarit burden scale items across caregivers of children with SCD, SSCAC, Elobeid, North Kordofan, Sudan, 2023, N: 123.

Total burden scores of caregivers with SCD were measured using the Zarit Burden Interview (ZBI-12), which consisted of 12 items as listed in Fig 2. Items were rated on a 5-point Likert scale (0–4), with higher scores indicating greater burden levels. The total score ranges from 0 to 48, classified as follows: 0–10: No to mild burden, 10–20: Mild to moderate burden, and > 20: High burden. (46.8%), (35.5%), and (17.7%) experienced mild, moderate, and severe levels of caregiving burden, respectively. The majority of the caregivers (80.4%) felt that they should be doing more for their SCD children, and (89.4%) reported feelings of uncertainty towards their caregiving duties. (42.3%) reported that caregiving interfered with their time for themselves, and (25.2%) described that their privacy was compromised due to caregiving duties. (26%) reported loss of control over their lives, (28.4%) reported feeling physically strained while caregiving, and (19.4%) reported feelings of anger around their SCD child. A majority (78%) reported that caregiving did not affect their relationships with others, and (65%) reported that their social life was never affected by their caregiving duties. Half (49.6%) of the caregivers reported never feeling stressed between caregiving and other responsibilities.

## Discussion

### Overview of the caregiving burdens of children with SCD

The majority of duties and responsibilities of care towards children with SCD are home-based and are carried out almost exclusively by the child’s caregivers. These duties range from daily administration of medications, including emergency management of the unpredictable sickle crises of their children [6]. Thus, this cross-sectional study aimed to demonstrate the physical and psychosocial burdens faced by caregivers of children with SCD attending the SSCAC, Elobeid, western Sudan, where the disease carries the highest prevalence in the country, which is mainly attributed to the high consanguinity rates in this population, as the disease is recessively inherited [26]. Our study showed similar results, as (69.1%) of participants were married to a relative.

### Sociodemographic characteristics of the caregivers

Most participants in this study were from the Bagara tribe (29%). Falata and Bideriya tribes represented (15.3%) and (14.5%) of the sample, respectively. This observation aligned with most of the published data on the prevalence of SCD in Western Sudan [11,12].

Previous studies concluded that caregivers with higher burden levels experienced worse quality of life [27]. Using the Zarit-12 burden score, (17.7%) of this study’s participants experienced severe burden levels, and (35.5%) experienced moderate burden. The majority of caregivers were females and biological mothers of the diseased child. Most of them were housewives who only completed primary education. This aligned with earlier studies made in populations with similar sociodemographic characteristics [13,14,16,28]. Caregivers with lower education levels experienced a greater burden compared to those with higher education. This is probably due to improper management of their children’s disease related to their limited knowledge of their illness. Our participants’ spouses mostly also only completed primary schooling, with (77.2%) of them working as free workers.

### Financial burdens

Jude demonstrated a strong association between financial distress and difficulty coping with the disease [23], and Khadija reported that more than 10% of caregivers’ income was spent on their SCD-diseased child’s health [29]. Financial burdens among the caregivers were highlighted by the multiple, frequent hospitalizations of their SCD child and long-term medications and transportation fees, while the majority of the caregivers in this sample were of low socioeconomic status (SES), with approximately (80%) of them having a monthly income of less than 100,000 SDG (< 160 USD). Studies in Ghana, Iran, and Iraq reported similar observations [6,30,31]. The low socioeconomic background of families of SCD was documented in many published studies [14,18,30–33]. A study revealed that caregivers of children with SCD experienced significant financial burdens due to work absenteeism and job loss related to their caregiving responsibilities [13]. (39.0%) of participants in this study came from outside Elobeid. These caregivers reported higher burden scores than their counterparts who lived in Elobeid, reflecting the constant physical and financial hardships these caregivers go through. This finding is also consistent with a study made in Iran, where families living in distant, rural areas suffered greatly from transportation burdens [6].

### Psychosocial burdens

Half the participants in our study reported constant stress between their caregiving duties and their other responsibilities, and (23%) felt as if they lost control over their lives. Taniya et al. reported that the high levels of stress among these caregivers, in addition to the child’s illness, were due to marital conflicts, death of children, and housing changes, which were indirect consequences of the child’s illness [34]. Magda et al. reported that (11.2%) of the caregivers experienced extremely severe anxiety [35]. Similar findings were made by Shaysteh et. al as they reported that families of children with SCD sometimes felt hopeless, angry, guilty, and anxious as a result of the chronicity of the disease [6]. (78.2%) of caregivers in this study had the constant feeling that they should do more for their children, and (58.1%) always felt that they could do a better job for their child, adding to the overall stress perceived by them. This finding reflects the great need for emotional support amongst these caregivers, especially from their close families and friends. A study made in Bahrain demonstrated that (73.7%) of the caregivers received satisfactory familial support, (81.2%) of it being emotional support [29]. On the other hand, Magda et al. reported that most caregivers lacked emotional support, which they needed to meet their children and families’ demands [35]. In some countries, like Iran, families of children with SCD faced stigmatisation from their surrounding communities because of their child’s illness. This could be due to the ignorance of people with the disease [6]. In contrast, SCD families in communities like those in Kenya and Nigeria, which are similar to the Sudanese communities, were never stigmatised [13,32]. This is due to the high prevalence of the disease in these communities, making it well known among them. (22%) of the caregivers stated that because of caregiving for their SCD child, they never had time for themselves, and (20%) stated that sometimes they felt their privacy was interrupted. A study by Andrew et al. compared the burden on caregivers of children with SCD to the normal US population. It was reported that caregivers’ private time was severely compromised due to caregiving. They also reported negative impacts on family activities and emotions [28]. On the other hand, (78%) of caregivers in our sample stated that their relationships with others (other family members, friends, neighbours, and colleagues) were never affected by their caregiving responsibilities, and (65.3%) stated their social life was never interrupted, but (20%) said sometimes it was. This result aligned with the results from Kenya, Nigeria, and Bahrain, in which most caregivers’ relationships were not affected by their caregiving responsibilities [13,29,32]. On the contrary, several studies did suggest that relationships between the caregivers and the other unaffected children were compromised. This is mainly caused by negligence of parental responsibilities and frequent absence from home due to the frequent hospitalisation of their diseased sibling [29,30].

### Physical burdens

(19%) of caregivers in this study reported that caregiving negatively affected their physical health, which is probably due to the constant caring for a child with such a chronic condition and very unpredictable crises, thus neglecting their physical well-being. This is lower than the results reported in a study in Kenya, in which (41%) of the caregivers reported always feeling tired throughout the day [13]. A study in Brazil reported that 27% of the caregivers complained of physical problems [27]. In spite of the increased perceived burdens among the caregivers in our study, they were generally content with their overall situation and were positive about their SCD children’s future, which helped them cope with the challenges of caregiving. This was highly due to their strong religious beliefs and tight familial connections. This was similar to what Ali et al. reported in Iraq [30].

### Characteristics of the children with SCD

This study revealed that caregivers of children aged 5–9 years old suffered the most. Only (55.6%) of school-aged children in our sample attended their classes regularly, and (26.4%) were never enrolled in school. (41.7%) of the caregivers reported that their children’s illness and multiple unpredictable sickle crises were the main culprits for their absence from school. Similar observations were reported in a study in Kenya, in which school-aged children missed 4 days a month due to symptoms related to their illness [13]. Most of them (75%) were diagnosed with the disease when they were younger than 1 year of age. This is in contrast with a study made in Iraq, where the majority of the children were diagnosed when they were older than 1 year old [30]. The early diagnosis observed in this study can be interpreted by the high prevalence of the disease in western Sudan, which is further expanded by the very high rates of consanguineous marriages in the community, making the disease well-known throughout the health sectors as well as amongst common people. The majority of the children in this sample were regularly administered folic acid (97.6%) and hydroxyurea (86.2%). This was a lot higher than reported results from an online survey made in the USA, in which only (50%) took hydroxyurea and (78%) took folic acid. [28]. Results from Kenya also reported lower rates of using hydroxyurea (60%) [13]. Most caregivers in a study in Ghana reported that their diseased children took folic acid with multivitamins and antibiotics regularly, but they refused to give them hydroxyurea because of its cost and perceived future side effects, like infertility [31].

## Study limitations

The study had a number of limitations. The primary limitation is that the final sample size (n = 123) was lower than the calculated 194 due to the Sudan conflict. The reduced sample size can have a great impact on the statistical power of the study’s results, and it is suggested that this study be replicated when conditions improve. This cross-sectional study design limits the ability to draw any causal inferences about the role of caregiving for a child with SCD on caregivers’ physical and psychosocial distress. Results from this study will require additional investigations within a well-powered longitudinal design and comparison groups to test a predictive, causal model. Another limitation was the interruption of the interview process due to caregivers being called by the medical staff and physicians, which could have affected the quality of the data. These interruptions could have affected the responses of the participants by disrupting the conversation flow and, thus, reducing the depth of responses. Moreover, the face-to-face interviews held by the authors could have contributed to potential recall bias and social desirability by the participants.

## Recommendations

It is recommended to consider the role of caregivers in the treatment of patients in the planning of the health system. This could be achieved through counselling sessions done with the caregivers during their visits for regular follow-up. It is also recommended to raise awareness in the community and inform the public about the disease through regular campaigns and encourage premarital screening, especially in those tribes where the disease is prevalent.

## Conclusion

This study highlights the significant physical and psychosocial burdens faced by caregivers of children with SCD living in Elobeid, western Sudan. (46.8%), (35.5%) and (17.7%) experienced mild, moderate, and severe levels of caregiving burden, respectively. These burdens were further exacerbated by the low socioeconomic status and lower education levels of these caregivers.

## Supporting information

S1 Table Baseline characteristics of caregivers of children with SCD participating in the study, SSCAC, Elobeid, North Kordofan, Sudan, 2023, N: 123. (DOCX)

S2 Table Characteristics of children with SCD participating in the study, SSCAC, Elobeid, North Kordofan, Sudan, 2023, N: 123. (DOCX)

S1 File STROBE Checklist. (DOCX)

S2 File Survey questionnaire in English. (DOCX)

S3 File Survey questionnaire in Arabic. (DOCX)

S4 File Data. Software data.
(DOCX)

## References

[1] Kato GJ, Piel FB, Reid CD, Gaston MH, Ohene-Frempong K, Krishnamurti L, et al. Sickle cell disease. Nat Rev Dis Primers. 2018;4:18010. doi: 10.1038/nrdp.2018.10 29542687

[2] Herrick JB. Peculiar elongated and sickle-shaped red blood corpuscles in a case of severe anemia. Arch Intern Med. 1910;6:517–21. PMC2588723 11501714

[3] Obied-sudan OS, Toro SA, Al M, Abdalrazag M, Bazie EA. Clinical presentation of sickle cell disease in patients admitted to Al. Journal of Medical - Clinical Research & Reviews. 2022;6(4):1–5.

[4] Salman FH, Elmahdi Z, Moawia S, Elnour B. Depression in Adolescent Sickle Cell Patients: A 2021 Sudan Study. 2021.

[5] Laurence B, George D, Woods D, Shosanya A, Katz RV, Lanzkron S, et al. The association between sickle cell disease and dental caries in African Americans. Spec Care Dentist. 2006;26(3):95–100. doi: 10.1111/j.1754-4505.2006.tb01430.x 16774185 PMC1786275

[6] Haghighi S, Rostami S, Jahani S. Caregiver suffering of the families of the patients with sickle cell: a qualitative study. 2020;10(1):158–62.

[7] Ismail WIM, Elnour M, Mustafa AEM. Evaluation of transcranial Doppler abnormalities in children with sickle cell disease in El-Obeid Specialized Children’s Hospital. J Family Med Prim Care. 2019;8(3):1176–81. doi: 10.4103/jfmpc.jfmpc_112_19 31041270 PMC6482797

[8] Hay SI, Gupta S, Weatherall DJ, Williams TN. Global burden of sickle cell anaemia in children under five, 2010 – 2050: modelling based on demographics, excess mortality, and interventions. 2013;10(7). 10.1371/journal.pmed.1001484 PMC3712914 23874164

[9] Rahimy MC, Gangbo A, Ahouignan G, Adjou R, Deguenon C, Goussanou S, et al. Effect of a comprehensive clinical care program on disease course in severely ill children with sickle cell anemia in a sub-Saharan African setting. Blood. 2003;102(3):834–8. doi: 10.1182/blood-2002-05-1453 12702514

[10] Archibald RG. A case of sickle cell anemia in the Sudan. N/A. 2023;10:389.

[11] Access O. The ethnic distribution of sickle cell disease in Sudan. 2014;8688:1–4. 10.11604/pamj.2014.18.13.3280 PMC4213521 25360197

[12] Daak AA, Elsamani E, Ali EH, Mohamed FA, Abdel-rahman ME. Sickle cell disease in western Sudan: genetic epidemiology and predictors of knowledge attitude and practices. Sudan J Paediatr. 2016;21(5):642–53. 10.1111/tmi.12689 PMC10699227 27028397

[13] Kuerten BG, Ms C, Brotkin S, Bonner M, Ayuku DO, Njuguna F. Psychosocial Burden of Childhood Sickle Cell Disease on Caregivers in Kenya. 2020;45(5):561–72. 10.1093/jpepsy/jsaa021 PMC7825476 32374404

[14] Madani BM, Raddadi RA l, Jaouni SA l, Omer M, Awa MA l. Quality of life among caregivers of sickle cell disease patients: a cross sectional study. 2018;:1–9. 10.1186/s12955-018-1009-5 PMC6131823 30200992

[15] Hassan AA, Mohamed AA. Physical and psychological burden on caregivers of children with intellectual developmental disabilities attending psychiatric outpatient – clinic - Khartoum state - Sudan. Journal of Psychiatry. 2020;4(2):1–9.

[16] Tweel XWVD, Hatzmann J, Ensink E, Lee JHVD, Peters M, Fijnvandraat K. Quality of life of female caregivers of children with sickle cell disease: a survey. 2008;93(4). 10.3324/haematol.11610 18322259

[17] Jacob SA, Bouck J, Daas R, Jackson MD, Lamotte JE, Carroll AE. Understanding caregiver burden with accessing sickle cell care in the Midwest and their perspective on telemedicine. 2023;:1–6. 10.1186/s12913-023-09383-x PMC10189684 37198614

[18] Bruzzese J, Usseglio J, Informationist S, Iannacci-manasia L, Diggs KA, Smaldone AM, et al. Mental and Emotional Health of Caregivers of Youth with Sickle Cell Disease: A Systematic Review. 2023;. PMC10683928 38015138

[19] Reader SK, Ruppe NM, Deatrick JA, Rash-ellis DL, Wadman JR, Miller RE. Clinical Practice in Pediatric Psychology Sickle Cell Disease: Informing the Adaptation of the Psychosocial Assessment Tool. 2017.

[20] Eldin A, Mustafa M. Improving the outcome of sickle cell disease patients in a resource limited setting Sudan sickle cell anemia centre (SSCAC): a promising and developing experience. Sudan Sickle Cell Anemia Centre (SSCAC). 2021;14(1).

[21] Cochran WG, Wiley J. Sampling Techniques. 3 ed. Wiley. 2023.

[22] Adam MA, Adam NK, Mohamed BA. Prevalence of sickle cell disease and sickle cell trait among children admitted to Al Fashir Teaching Hospital North Darfur State, Sudan. BMC Res Notes. 2019;12(1):659. doi: 10.1186/s13104-019-4682-5 31619285 PMC6796395

[23] Bachner YG. Preliminary assessment of the psychometric properties of the abridged Arabic version of the Zarit Burden Interview among caregivers of cancer patients. Eur J Oncol Nurs. 2013;17(5):657–60. doi: 10.1016/j.ejon.2013.06.005 23867141

[24] Bédard M, Molloy DW, Squire L, Dubois S, Lever JA, O’Donnell M. The Zarit Burden Interview: a new short version and screening version. Gerontologist. 2001;41(5):652–7. doi: 10.1093/geront/41.5.652 11574710

[25] Bank W. Poverty [Internet]. 2022. Available from: https://worldbank.org/en/topic/poverty

[26] Bayoumi RA, Taha TS, Saha N. A study of some genetic characteristics of the Fur and Baggara tribes of the Sudan. Am J Phys Anthropol. 1985;67(4):363–70. doi: 10.1002/ajpa.1330670408 2932917

[27] da Silva LBL, Ivo ML, de Souza AS, Pontes ERJC, Pinto AMAC, de Araujo OMR. The burden and quality of life of caregivers of sickle cell anemia patients taking hydroxyurea versus those not taking hydroxyurea. Rev Bras Hematol Hemoter. 2012;34(4):270–4. doi: 10.5581/1516-8484.20120070 23049439 PMC3460397

[28] Campbell A, Rizio AA, Mccausland KL, Iorga S, Yen GP, Paulose J. The burden of sickle cell disease on children and their caregivers: Caregiver reports of children’s health-related quality of life and school experiences, caregiver burden, and their association with frequency of vaso-occlusive crises. 2023. 10.2147/PROM.S419607 PMC10693247 38046665

[29] Al Saif K, Abdulla FM, Alrahim A, Abduljawad S, Matrook Z, Abdulla JJ, et al. Caregivers’ experience of seeking care for adolescents with sickle cell disease in a tertiary care hospital in Bahrain. PLoS One. 2022;17(4):e0266501. doi: 10.1371/journal.pone.0266501 35390069 PMC8989311

[30] Habeeb AL, MEA A, Hassan K, Abd B, Hussein AL. Psychosocial impact of sickle cell disease on families in Basra, southern Iraq; an experience of caregivers. 2015;5(4):41–52.

[31] Mumuni ND, Osman W, Alhassan BA. Burden experienced by informal caregivers of children with sickle cell disease (SCD): a qualitative exploratory study at Tamale Teaching. 2023. 10.1136/bmjopen-2022-066311 PMC10083813 37024250

[32] Ohaeri JU, Shokunbi WA. Psychosocial burden of sickle cell disease on caregivers in a Nigerian setting. 2002. PMC2568407 12510705

[33] Motta AB. The psychological impact on family caregivers of children and adolescents with sickle cell anemia O impacto psicológico em familiares cuidadores de crianças e adolescentes com anemia falciforme. 2021.

[34] Varughese TE, Otr L, Hoyt CR, Hotta AJL, Ikemenogo PA. Stress and the home environment in caregivers of children with sickle cell. 2020. 1–9. 10.1093/jpepsy/jsaa016 PMC12104525 32232470

[35] Yousif M, Abdelrahman A, Al Jamea LH, Al-Yami FS, Woodman A. Psychosocial Impact of Sickle Cell Disease and Diabetes Mellitus on Affected Children and Their Parents in Khartoum State, Sudan. J Trop Pediatr. 2022;68(3):fmac042. doi: 10.1093/tropej/fmac042 35641127

