# Supplementary material for: Silent struggles: Assessing physical and psychosocial burdens among caregivers of children with sickle cell disease in western Sudan–A cross-sectional study
Source: PLoS One. 2025 Nov 25;20(11):e0336469. doi: 10.1371/journal.pone.0336469 (PMC12646449; doi:10.1371/journal.pone.0336469)
Supplement: S1 Table — (DOCX) [file pone.0336469.s001.docx]

Table 1: Baseline characteristics of caregivers of children with SCD participating in the study, SSCAC, Elobeid, North Kordofan, Sudan, 2023, N: 123.

| **Variable** | **N** | **%** | **Total Zarit** | |
| --- | --- | --- | --- | --- |
|  |  |  | **Median (IQR)** | **P value** |
| **Age** |  |  |  | 0.092^a^ |
| <20 Years | 2 | 1.60% | 28 (16-40) |  |
| 20-30 Years | 51 | 41.5% | 10 (8-18) |  |
| 31-40 Years | 44 | 35.8% | 10 (8-17) |  |
| >40 Years | 26 | 21.1% | 15 (10-20) |  |
| **Relationship to child** |  |  |  | 0.909^a^ |
| Mother | 104 | 84.6% | 12 (8-20) |  |
| Father | 13 | 10.6% | 13 (9-16) |  |
| Sibling | 1 | 0.80% | 16 (16-16) |  |
| Aunt | 3 | 2.40% | 17 (8-18) |  |
| Grandparent | 2 | 1.60% | 9 (8-10) |  |
| **Gender of the caregiver** |  |  |  | 0.862^b^ |
| Male | 13 | 10.6% | 13 (9-16) |  |
| Female | 110 | 89.4% | 12 (8-19) |  |
| **Marital status** |  |  |  | 0.906^a^ |
| Single | 2 | 1.60% | 12 (8-16) |  |
| Married | 112 | 91.1% | 12 (8-18) |  |
| Divorced | 3 | 2.40% | 17 (4-32) |  |
| Widowed | 6 | 4.90% | 15 (9-22) |  |
| **Area** |  |  |  | **0.028^b^** |
| Elobeid | 75 | 61.0% | 10 (8-17) |  |
| Outside Elobeid | 48 | 39.0% | 16 (8-25) |  |
| **Residence** |  |  |  | 0.057^b^ |
| Rural | 34 | 27.6% | 14 (9-25) |  |
| Urban | 89 | 72.4% | 11 (8-17) |  |
| **Total children** |  |  |  | 0.673^a^ |
| 1 | 11 | 8.90% | 10 (6-20) |  |
| 2 | 23 | 18.7% | 9 (6-16) |  |
| 3 | 21 | 17.1% | 10 (8-19) |  |
| >3 | 67 | 54.5% | 12 (8-19) |  |
| **Total children with SCD** |  |  |  | 0.456^a^ |
| 1 | 101 | 82.1% | 11 (8-18) |  |
| 2 | 20 | 16.3% | 16 (10-21) |  |
| 3 | 1 | 0.80% | 10 (10-10) |  |
| >3 | 1 | 0.80% | 16 (16-16) |  |
| **Monthly income** |  |  |  | 0.369^a^ |
| <50,000 SDG | 53 | 43.1% | 13 (8-20) |  |
| 50,000-100,000 SDG | 49 | 39.8% | 10 (7-17) |  |
| 100,000-200,000 SDG | 12 | 9.80% | 14 (8-18) |  |
| >200,000 SDG | 9 | 7.30% | 15 (10-18) |  |
| *p-value: significant level at 95% confidence interval, IQR: Interquartile range* | | | | |
| *^a^Kruskal-Wallis test, ^b^Mann-whitney U test* | | | | |
