# Supplementary material for: Silent struggles: Assessing physical and psychosocial burdens among caregivers of children with sickle cell disease in western Sudan–A cross-sectional study
Source: PLoS One. 2025 Nov 25;20(11):e0336469. doi: 10.1371/journal.pone.0336469 (PMC12646449; doi:10.1371/journal.pone.0336469)
Supplement: S2 Table — (DOCX) [file pone.0336469.s002.docx]

Table 3: Characteristics of children participating in the study, Elobeid, North Kordofan, Sudan, 2023, N: 123.

| **Variable** | **N** | **%** | **Total Zarit** | |
| --- | --- | --- | --- | --- |
|  |  |  | **Median (IQR)** | **P value** |
| **Child gender** |  |  |  | 0.635^b^ |
| Male | 64 | 52.0% | 12 (8-20) |  |
| Female | 59 | 48.0% | 10 (8-17) |  |
| **Child age** |  |  |  | **0.008**^a^ |
| 0-4 Years | 46 | 37.4% | 9 (6-16) |  |
| 5-9 Years | 40 | 32.5% | 16 (10-25) |  |
| 10-13 Years | 29 | 23.6% | 10 (8-18) |  |
| 14-18 Years | 8 | 6.50% | 9 (8-15) |  |
| **Diagnosis age** |  |  |  | 0.430^a^ |
| <6 month | 27 | 22.0% | 14 (8-25) |  |
| 6-12 months | 65 | 52.8% | 11 (8-17) |  |
| >12 months | 31 | 25.2% | 10 (6-20) |  |
| **Hydroxyurea intake** |  |  |  | 0.397^a^ |
| Regularly | 106 | 86.2% | 12 (8-18) |  |
| Irregularly | 5 | 4.10% | 16 (11-32) |  |
| Never | 12 | 9.80% | 13 (8-26) |  |
| **Folic acid intake** |  |  |  | 0.092^a^ |
| Regularly | 120 | 97.6% | 11 (8-18) |  |
| Irregularly | 1 | 0.80% | 38 (38-38) |  |
| Never | 2 |  | 24 (15-32) |  |
| **Health insurance** |  |  |  | 0.996^b^ |
| Yes | 88 | 71.5% | 12 (8-18) |  |
| No | 35 | 28.5% | 12 (8-20) |  |
| **School attendance** |  |  |  | 0.293^a^ |
| Regularly | 40 | 32.5% | 10 (8-17) |  |
| Irregularly | 13 | 10.6% | 14 (12-20) |  |
| Never | 70 | 56.9% | 12 (8-20) |  |
| **If irregularly or never to the previous question, why?** |  |  |  | **0.003**^a^ |
| Below school age (<5 years) | 51 | 61.4% | 9 (6-16) |  |
| Illness | 30 | 36.1% | 17 (12-22) |  |
| Financial | 2 | 2.40% | 26 (20-32) |  |
| *p-value: significant level at 95% confidence interval, IQR: Interquartile range* | | | | |
| *^a^Kruskal-Wallis test, ^b^Mann-whitney U test* | | | | |
