# Supplementary material for: Silent struggles: Assessing physical and psychosocial burdens among caregivers of children with sickle cell disease in western Sudan–A cross-sectional study
Source: PLoS One. 2025 Nov 25;20(11):e0336469. doi: 10.1371/journal.pone.0336469 (PMC12646449; doi:10.1371/journal.pone.0336469)
Supplement: S1 File — (DOCX) [file pone.0336469.s003.docx]

STROBE Statement—checklist of items that should be included in reports of observational studies

|  | **Item No.** | **Recommendation** | **Page  No.** | **Relevant text from manuscript** |
| --- | --- | --- | --- | --- |
| **Title and abstract** | 1 | (*a*) Indicate the study’s design with a commonly used term in the title or the abstract | 2 | This observational, cross-sectional facility-based study aimed to assess the caregivers' physical and psychosocial burdens. |
|  |  | (*b*) Provide in the abstract an informative and balanced summary of what was done and what was found | 2 | A total of 123 caregivers who attended the Sudan Sickle Cell Anaemia Centre, El Obeid, western Sudan, were interviewed using the abridged Arabic Zarit Burden Interview Scale (ZBI-A) between March 15 and April 12, 2023. Data was then analysed using SPSS v.20 and summarised into medians and interquartile ranges. A p-value of <0.05 was considered statistically significant. The Mann-Whitney U test and Kruskal-Wallis tests were used to identify the characteristics of caregivers and their SCD children that were associated with the total Zarit burden score of the caregivers. (84.6%) of caregivers were biological mothers, (41.5%) were (20-30) years old, and (39.0%) were from outside Elobeid. Most mothers (37.4%) and fathers (39.8%) only completed primary schooling; thus, most mothers (84.6%) were housewives, and most fathers (77.2%) were free workers. (82.9%) had a family monthly income level of <100,000 SDG (<167 USD). (46.8%), (35.5%), and (17.7%) experienced mild, moderate, and severe levels of caregiving burden, respectively. Total Zarit burden scores of the caregivers were significantly associated with residing outside Elobeid (p=0.028), lower maternal and paternal educational levels (p=0.008) (p=0.036), respectively, lower paternal employment status (p=0.034) and those whose children with SCD were aged 5-9 years (p=0.008). In conclusion, (46.8%), (35.5%) and (17.7%) of the participants experienced mild, moderate, and severe levels of caregiving burden, respectively. |
| **Introduction** | | | |  |
| Background/rationale | 2 | Explain the scientific background and rationale for the investigation being reported | 3 | Sickle Cell Disease (SCD) is a chronic blood disorder that starts manifesting in the first six months of life. (1). The first description of SCD was made in the United States by Dr. James Herrick at the beginning of the 20th century. He described the disease as "peculiar, elongated, and sickle-shaped red blood corpuscles in a case of severe anaemia" (2). Sickling of the red blood cells gives rise to the two main clinical manifestations of the disease: hemolytic anemia (3,4) and vasocclusion, which follows the sticking of the sickled red cells in the small blood vessels. Vasocclusion, in turn, leads to reduced blood flow and tissue necrosis, which is followed by episodes of severe pain termed “sickle cell crisis” (5). These sickle cell crises are the leading causes of distress among SCD patients, accounting for nearly 90% of SCD-related hospital admissions. (6). They are characterised by being unpredictable and are usually variable in severity, duration, and location (7).  It is estimated that each year, 300,000 are born with SCD, with 70% of them in Africa alone. (8). Death rates of children in Africa reach up to 90%. These extremely high mortality rates in African SCD patients are the result of cultural background, a paucity of public awareness of the disease, and a scarcity of healthcare centres, accounting for limited early detection of the disease, and thus it remains undiagnosed and untreated. The recent introduction of simple and comprehensive SCD programs in sub-Saharan African countries has significantly reduced mortality rates (9). This disease was first reported in Sudan by Archibald in 1926 (10). It poses a major health problem in the country, especially in the western region, where the prevalence of the disease is the highest (30.4%) (11), especially in the Messeriya tribe, a branch of the Bagara tribe, in which it is estimated to be 18.2% in Kordofan and 30.4% in Darfur (12).  SCD places a significant psychosocial and economic burden on the children’s caregivers. This burden can extend to involve the whole community, especially in LMICs like Sudan. Generally, the caregiving burden consists of two components: subjective and objective burden. Objective burden includes measurable effects such as disrupted interrelationships, economic hardships, loss of jobs, and social and leisure activities. In contrast, subjective burden is demonstrated by the psychological sufferings experienced by the caregivers, like depression, feelings of shame, and embarrassment. Caregiving for children with serious chronic medical conditions in general is highly demanding and is associated with pervasive stresses correlating with poor physical and psychological health outcomes (13,14,1516). It is worth noting that a high percentage of families with SCD-affected children face difficulties with transportation and in accessing medical centres, resulting in less than 70% of them receiving the appropriate medical care. Furthermore, sickle cell centres are few and are usually centralised in urban cities, rendering it difficult for those living in distant, rural residences to seek adequate medical care for their SCD children (17). Psychosocial distress perceived by the caregivers is associated with lower psychological health of their SCD children. Moreover, it has a direct negative impact on the SCD-related health outcomes of their children, as evidenced by worse perceived sickle cell pain, poorer child health outcomes, and increased use of health care. Previous studies associated poorer caregiver psychological health with poorer treatment adherence for their SCD children (18,19). |
| Objectives | 3 | State specific objectives, including any prespecified hypotheses | 4 | This study, therefore, aims to assess the physical and psychosocial burdens experienced by caregivers of children with SCD in Elobeid, western Sudan and to assess the association between the sociodemographic characteristics of the caregivers and the level of burdens perceived by them. |
| **Methods** | | | |  |
| Study design | 4 | Present key elements of study design early in the paper | 5 | This study utilised an observational, descriptive, cross-sectional, facility-based design. This study design was used as it was feasible and suitable for both the context and the participants. It provides timely identification of the current burden levels and possible sociodemographic risk factors. It also allows comparability with other similar studies done in LMICs. |
| Setting | 5 | Describe the setting, locations, and relevant dates, including periods of recruitment, exposure, follow-up, and data collection | 5  6 | The study was conducted at the Sudan Sickle Cell Anaemia Centre (SSCAC), located in El Obeid Specialized Pediatric Hospital, El Obeid, North Kordofan, Sudan, from March 15, 2023, to April 12, 2023.  Data collection took place weekly on Wednesdays from 8:00 AM to 2:00 PM. Data was then collected from 124 caregivers during their waiting time at the SSCAC. Caregivers were interviewed using a semi-structured questionnaire and the Arabic abridged Zarit Burden Interview Scale (ZBI-A). The researcher conducted individual interviews in a comfortable and private setting. Verbal consent was obtained after explaining the study's purpose and assuring confidentiality. Each |
| Participants | 6 | *Cross-sectional study*—Give the eligibility criteria, and the sources and methods of selection of participants | 5 | The study included caregivers of SCD children who were younger than 18 years of age and who lived with their SCD children in the same house. The study excluded caregivers of children older than 18 years, patients acting as their own caregivers, and caregivers with a diagnosed psychiatric illness. |
| Variables | 7 | Clearly define all outcomes, exposures, predictors, potential confounders, and effect modifiers. Give diagnostic criteria, if applicable | 7 | A semi-structured questionnaire was developed by the researcher to gather socio-demographic information about the caregivers and their SCD children, including age, marital status, residence, tribe, educational level, employment status, and monthly income. The abridged Arabic version of the Zarit Burden Interview Scale (ZBI-A) was used to measure caregiver burden. It consists of 12 items rated on a 5-point Likert scale (0–4), with higher scores indicating a greater burden. The total score ranges from 0 to 48, classified as follows: 0–10: No to mild burden, 10–20: Mild to moderate burden, and > 20: High burden |
| Data sources/ measurement | 8* | For each variable of interest, give sources of data and details of methods of assessment (measurement). Describe comparability of assessment methods if there is more than one group | 6 | Caregivers were interviewed using a semi-structured questionnaire and the Zarit Burden Interview Scale (ZBI-12).  Only one group was studied. |
| Bias | 9 | Describe any efforts to address potential sources of bias | 7 | A pilot study, which was conducted at the start of the study with 12 participants, confirmed the comprehensibility of the (ZBI-A), which was in standard Arabic, with the participants who spoke in the local Sudanese dialect. Data from the pilot study were excluded from the final analysis to avoid contamination. |
| Study size | 10 | Explain how the study size was arrived at | 6 | Cochran’s formula for sample size estimation was used.  n=Z^2*p*(1-p)/e^2  Where:  n = Required sample size  z = Z-score corresponding to the desired confidence level (1.96 corresponds to 95% confidence level)  p = Estimated prevalence (14.8%).  e = Margin of error (here is 5%)  The calculated sample size was 194 participants. However, due to the onset of the war in Sudan on April 15, 2023, data collection was disrupted, and the SSCAC was shut down. Consequently, only 124 caregivers were included in the study. |

Continued on next page

| Quantitative variables | 11 | Explain how quantitative variables were handled in the analyses. If applicable, describe which groupings were chosen and why | 6 | The abridged Arabic version of the Zarit Burden Interview Scale (ZBI-A) was used to measure caregiver burden. It consists of 12 items rated on a 5-point Likert scale (0–4), with higher scores indicating a greater burden. The total score ranges from 0 to 48, classified as follows: 0–10: No to mild burden, 10–20: Mild to moderate burden, and > 20: High burden |
| --- | --- | --- | --- | --- |
| Statistical methods | 12 | (*a*) Describe all statistical methods, including those used to control for confounding | 7 | Data was analyzed by SPSS v. 20. Data was skewed, so descriptive data was summarized into medians and interquartile ranges. Non-parametric tests, Mann-Whitney U test (for two groups) and Kruskal-Wallis’s test (for three or more groups), were used to identify the characteristics of the caregivers and their SCD children that were associated with the total Zarit burden score of the caregivers. A p-value of <0.05 was considered statistically significant. |
|  |  | (*b*) Describe any methods used to examine subgroups and interactions | 7 | Mann-Whitney U test and Kruskal-Wallis’s test, were used to identify the characteristics of the caregivers and their SCD children that were associated with the total Zarit burden score of the caregivers. |
|  |  | (*c*) Explain how missing data were addressed |  | No missing data |
|  |  | (*d*) *Cohort study*—If applicable, explain how loss to follow-up was addressed  *Case-control study*—If applicable, explain how matching of cases and controls was addressed  *Cross-sectional study*—If applicable, describe analytical methods taking account of sampling strategy |  | Not applicable |
|  |  | (*e*) Describe any sensitivity analyses |  | Not addressed. Sensitivity analysis not performed. |
| **Results** | | | | |
| Participants | 13* | (a) Report numbers of individuals at each stage of study—eg numbers potentially eligible, examined for eligibility, confirmed eligible, included in the study, completing follow-up, and analysed | 7 | A total of 123 caregivers were included in this study. |
|  |  | (b) Give reasons for non-participation at each stage | 6 | One caregiver was excluded from the study for having a child older than 18 years old. |
|  |  | (c) Consider use of a flow diagram |  | Not available |
| Descriptive data | 14* | (a) Give characteristics of study participants (eg demographic, clinical, social) and information on exposures and potential confounders | 7 | The majority of caregivers (84.6%) were biological mothers, and (10.6%) were fathers. (41.5%) of caregivers in the age range of 20-30 years old. (61%) of caregivers lived in Elobeid, while (39.0%) lived outside Elobeid. (16.3%) of the caregivers had two children with SCD. (92.7%) of the participants had a family monthly income level of less than 200,000 SDG. Regarding mothers’ education, (37.4%) had primary education, and (18.7%) had university degrees. Similarly, (39.8%) of fathers completed primary education, but only (13.8%) had a university degree or higher. In terms of occupation, most (84.6%) mothers were housewives, and the majority (77.2%) of fathers were free workers. |
|  |  | (b) Indicate number of participants with missing data for each variable of interest |  | No missing data |
|  |  | (c) *Cohort study*—Summarise follow-up time (eg, average and total amount) |  | Not applicable |
| Outcome data | 15* | *Cohort study*—Report numbers of outcome events or summary measures over time |  | Not applicable |
|  |  | *Case-control study—*Report numbers in each exposure category, or summary measures of exposure |  | Not applicable |
|  |  | *Cross-sectional study—*Report numbers of outcome events or summary measures | 15 | (46.8%), (35.5%) and (17.7%) experienced mild, moderate, and severe levels of caregiving burden, respectively |
| Main results | 16 | (*a*) Give unadjusted estimates and, if applicable, confounder-adjusted estimates and their precision (eg, 95% confidence interval). Make clear which confounders were adjusted for and why they were included | 7  10 | In comparison with those living in Elobeid, caregivers resided outside Elobeid showed a significant association with total burden score (p-value = 0.028).  The lower educational level for parents' is strongly associated with the Zarit Burden Interview Scale (ZBI-A) (for mothers, p-value = 0.008), (for father’s, p-value = 0.036). Lower father’s employment status is strongly associated with the Zarit Burden Interview Scale (ZBI-A) (p-value = 0.034) |
|  |  | (*b*) Report category boundaries when continuous variables were categorized | 15 | Total burden scores of caregivers with SCD were measured using the Zarit Burden Interview (ZBI-12), which consisted of 12 items items rated on a 5-point Likert scale (0–4), with higher scores indicating a greater burden. The total score ranges from 0 to 48, classified as follows: 0–10: No to mild burden, 10–20: Mild to moderate burden, and > 20: High burden |
|  |  | (*c*) If relevant, consider translating estimates of relative risk into absolute risk for a meaningful time period |  | Not applicable |

Continued on next page

| Other analyses | 17 | Report other analyses done—eg analyses of subgroups and interactions, and sensitivity analyses |  | Not applicable |
| --- | --- | --- | --- | --- |
| **Discussion** | | | | |
| Key results | 18 | Summarise key results with reference to study objectives |  | Caregivers of children with SCD in western Sudan experienced significant burden. 17.7% severe, 35.5% moderate, and 46.8% mild burden levels. Higher burden levels were associated with living outside Elobeid (p=0.028), low parental education (mothers, p=0.008, fathers, p=0.036) and lower paternal employment status (p=0.034). Caregivers of children 5-9 years old reported the highest burden levels (p=0.008) and a strongly significant statistical association was discovered between irregular school attendance and the financial burden (p-value = 0.003) |
| Limitations | 19 | Discuss limitations of the study, taking into account sources of potential bias or imprecision. Discuss both direction and magnitude of any potential bias | 19 | The study had a number of limitations. The primary limitation is the small number of caregivers interviewed due to the onset of the war in Sudan on April 15, 2023, in which the SSCAC was shut down and the data collection process was disrupted. This cross-sectional study design limits the ability to draw any causal inferences about the role of caregiving for a child with SCD on caregivers’ physical and psychosocial distress. Results from this study will require additional investigations within a well-powered longitudinal design and comparison groups to test a predictive, causal model. Another limitation was the interruption of the interview process due to caregivers being called by the medical staff and physicians, which could have affected the participants’ responses and hence the quality of the data. Moreover, the face-to-face interviews held by the authors could have contributed to potential recall bias and social desirability by the participants. |
| Interpretation | 20 | Give a cautious overall interpretation of results considering objectives, limitations, multiplicity of analyses, results from similar studies, and other relevant evidence | 16 | Using the Zarit-12 burden score, (17.7%) of this study’s participants experienced severe burden levels, and (35.5%) experienced moderate burden. Caregivers with lower education levels experienced greater burden compared to those with higher education. This is probably due to improper management of their children’s disease relating to their limited knowledge of their illness. Financial burdens among the caregivers were highlighted by the multiple, frequent hospitalizations of their SCD child and long-term medications and transportation fees, while the majority of the caregivers in this sample were of low socioeconomic status (SES), with approximately (80%) of them having a monthly income of less than 100,000 SDG (< 160 USD). Studies in Ghana, Iran, and Iraq reported similar observations. (39.0%) of participants in this study came from outside Elobeid. These caregivers reported higher burden scores than their counterparts who lived in Elobeid, reflecting the constant physical and financial hardships these caregivers go through. Half the participants in our study reported constant stress between their caregiving duties and their other responsibilities and (23%) felt as if they lost control over their lives. Taniya et al. reported that the high levels of stress among these caregivers, in addition to the child’s illness, were due to marital conflicts, death of children, and housing changes, which were indirect consequences of the child’s illness (34). Magda et al. reported that (11.2%) of the caregivers experienced extremely severe anxiety (35). Similar findings were made by Shaysteh et. al as they reported that families of children with SCD sometimes felt hopeless, angry, guilty, and anxious as a result of the chronicity of the disease (6). (78.2%) of caregivers in this study had the constant feeling that they should do more for their children, and (58.1%) always felt that they could do a better job for their child, adding to the overall stress perceived by them. In spite of the increased perceived burdens among the caregivers in our study, they were quite content with their overall situation and were positive about their SCD children’s future, which helped them cope with the challenges of caregiving. This was highly due to their strong religious beliefs and tight familial connections. This was similar to what Ali et al. reported in Iraq (30). |
| Generalisability | 21 | Discuss the generalisability (external validity) of the study results | 16 | Findings can be generalised to similar developing, sub-Saharan countries with similar consanguinity rates and caregiving situations (Kenya, Ghana, Nigeria). |
| **Other information** | |  | | |
| Funding | 22 | Give the source of funding and the role of the funders for the present study and, if applicable, for the original study on which the present article is based | 21 | This research did not receive any financial support. |

*Give information separately for cases and controls in case-control studies and, if applicable, for exposed and unexposed groups in cohort and cross-sectional studies.

**Note:** An Explanation and Elaboration article discusses each checklist item and gives methodological background and published examples of transparent reporting. The STROBE checklist is best used in conjunction with this article (freely available on the Web sites of PLoS Medicine at http://www.plosmedicine.org/, Annals of Internal Medicine at http://www.annals.org/, and Epidemiology at http://www.epidem.com/). Information on the STROBE Initiative is available at www.strobe-statement.org.
