# Supplementary material for: Silent struggles: Assessing physical and psychosocial burdens among caregivers of children with sickle cell disease in western Sudan–A cross-sectional study
Source: PLoS One. 2025 Nov 25;20(11):e0336469. doi: 10.1371/journal.pone.0336469 (PMC12646449; doi:10.1371/journal.pone.0336469)
Supplement: S2 File — (DOCX) [file pone.0336469.s004.docx]

# **S2 File. Survey questionnaire in english**

1. Demographic characteristics:

**1.1 Caregivers' characteristics:**

1. **Serial no.** …………………………………………………………
2. **Caregiver relationship to sickler child:** Mother ( ) Father ( ) Sibling ( ) Aunt/Uncle ( ) Grandparent ( )
3. **Gender:**  Male ( ) Female ( )
4. **Area of living:**  Elobeid ( ) Outside Elobeid ( )
5. **Residence :**Rural ( ) Urban ( )
6. **Age:**  >20 ( ) 20-30 ( ) 31-40 ( ) > 40 years ( ).
7. **Marital status:** Single ( ) Married ( ) Separated ( ) Widowed ( ).
8. **Mother’s education:** Illiteracy ( ) Khalwa ( ) Primary ( ) Secondary ( ) University ( ) Postgraduate ( )
9. **Father’s education:** Illiteracy ( ) Khalwa ( ) Primary ( ) Secondary ( ) University ( ) Postgraduate ( ).
10. **Father’s occupation:** Employee ( ) Free work ( ) Unemployed. ( )
11. **Mother’s occupation:**  Housewife ( ) Worker ( ) Employee ( )
12. **Father’s tribe:** Bagara ( ) Bideryia ( ) Gawam’aa ( ) Falata ( ) Dar Hamid ( ) Bargo ( ) Barno ( ) Galaba Hawara ( ) Others: ( ) specify: ……
13. **Mother’s tribe:** Bagara ( ) Bideryia **( )** Gawam’aa ( ) Falata ( ) Dar Hamid ( ) Bargo ( ) Barno ( ) Galaba Hawara ( ) Others ( ) specify: ……
14. **Family monthly income:** <50,0000 SDG ( ) 50,000 - 100,000 SDG ( ) 100,000 - 200,000 SDG ( ) >200,000 SDG ( )
15. **Total number of children in the family?** 1 ( ) 2 ( ) 3 ( ) >3 ( )
16. **Total number of children diagnosed with SCD?** 1 ( ) 2 ( ) 3 ( ) >3 ( )

**1.2 Care recipient characteristics:**

1. **Gender of child:** Male ( ) Female ( )
2. **Child age (in years):**  0-4 ( ) 5-9 ( ) 10-13 ( ) 14-18 ( )
3. **Age at First Diagnosis (in months):** < 6 ( ) 6-12 ( ) > 12 ( )
4. **Does the child take Hydroxyurea?** Regularly ( ) Irregularly ( ) Never ( )
5. **Does the child take Folic Acid?** Regularly ( ) Irregularly ( ) Never ( )
6. **Does the child have health insurance:**  Yes ( ) No ( )
7. **Does the child go to school:** Regularly ( ) Irregularly ( ) Never ( )
8. **If the child never went to school/ went irregularly, why?** Young age ( ) Illness ( ) Financial ( ) Psychological ( )

**Zarit Caregiver Burden Assessment (Short, 12-items)**

The following is a list of statements that reflect how people sometimes feel when taking care of another person. After reading each statement, indicate how often you experience the feelings listed by circling the number that best corresponds to the frequency of these feelings.

|  | **Never** | **Rarely** | **Sometimes** | **Frequently** | **Nearly Always** |
| --- | --- | --- | --- | --- | --- |
| 1) Do you feel you don’t have enough time for yourself? | 0 | 1 | 2 | 3 | 4 |
| 2) Do you feel stressed between caring and meeting other responsibilities? | 0 | 1 | 2 | 3 | 4 |
| 3) Do you feel angry when you are around your relative? | 0 | 1 | 2 | 3 | 4 |
| 4) Do you feel your relative affects your relationship with others in a negative way? | 0 | 1 | 2 | 3 | 4 |
| 5) Do you feel strained when are around your relative? | 0 | 1 | 2 | 3 | 4 |
| 6) Do you feel your health has suffered because of your involvement with your relative? | 0 | 1 | 2 | 3 | 4 |
| 7) Do you feel you don’t have as much privacy as you would like, because of your relative? | 0 | 1 | 2 | 3 | 4 |
| 8) Do you feel your social life has  suffered because you are caring for your relative? | 0 | 1 | 2 | 3 | 4 |
| 9) Do you feel you have lost control of your life since your relative’s illness? | 0 | 1 | 2 | 3 | 4 |
| 10) Do you feel uncertain about what to do about relative? | 0 | 1 | 2 | 3 | 4 |
| 11) Do you feel you should be doing more for your relative? | 0 | 1 | 2 | 3 | 4 |
| 12) Do you feel you could do a better job in caring for your relative? | 0 | 1 | 2 | 3 | 4 |

Scoring Instructions: Add Items 1‐12 Total 1-12 (maximum score = 48)
