# Supplementary material for: Silent struggles: Assessing physical and psychosocial burdens among caregivers of children with sickle cell disease in western Sudan–A cross-sectional study
Source: PLoS One. 2025 Nov 25;20(11):e0336469. doi: 10.1371/journal.pone.0336469 (PMC12646449; doi:10.1371/journal.pone.0336469)
Supplement: S3 File — (DOCX) [file pone.0336469.s005.docx]

**S3 File. Survey questionnaire in arabic**

1. الخصائص الديموغرافية:

**1.1 خصائص مقدمي الرعاية:**

1. **الرقم التسلسلي** ..................................................................
2. **علاقة مقدم الرعاية بالطفل المنجل:** الأم ( ) الأب ( ) الأخ ( ) العمة / العم ( ) الجد ( )
3. **الجنس:**  ذكر ( ) أنثى ( )
4. **منطقة المعيشة:** الأبيض ( ) خارج الأبيض ( )
5. **السكن :**ريفي ( ) حضري ( )
6. **العمر:**  >20 () 20-30 () 31-40 () > 40 سنة ().
7. **الحالة الاجتماعية:** أعزب ( ) متزوج ( ) منفصل ( ) أرمل ( ) .
8. **تعليم الأم :** الأمية ( ) خلوة ( ) الابتدائي ( ) الثانوي ( ) الجامعة ( ) الدراسات العليا ( )
9. **تعليم الأب:** الأمية ( ) خلوة ( ) الابتدائي ( ) الثانوي ( ) الجامعة ( ) الدراسات العليا ( ).
10. **مهنة الأب:** موظف ( ) عمل حر ( ) عاطل عن العمل. ( )
11. **مهنة الأم:**  ربة منزل ( ) عاملة ( ) موظفة ( )
12. **قبيلة الأب:** بقارة () بيدرية **()** جوامعة () فلاتة () دار حامد () برقو () برنو () جلابة هوارة () أخرى ()
13. **قبيلة الأم:** بقارة () بيدرية **()** جوامعة () فلاتة () دار حامد () برقو () برنو () جلابة هوارة () أخرى ()
14. **الدخل الشهري للأسرة:** <50,0000 جنيه سوداني () 50,000 - 100,000 جنيه سوداني () 100,000 - 200,000 جنيه سوداني () >200,000 جنيه سوداني ()
15. **إجمالي عدد الأطفال في الأسرة؟** 1 ( ) 2 ( ) 3 ( ) >3 ( )
16. **إجمالي عدد الأطفال الذين تم تشخيص إصابتهم بداء الكريات المنجلية؟** 1 ( ) 2 ( ) 3 ( ) >3 ( )

**1.2 خصائص متلقي الرعاية:**

1. **جنس الطفل:** ذكر ( ) أنثى ( )
2. **عمر الطفل (بالسنوات):**  0-4 ( ) 5-9 ( ) 10-13 ( ) 14-18 ( )
3. **العمر عند التشخيص الأول (بالشهور):** < 6 () 6-12 () > 12 ()
4. **هل يتناول الطفل هيدروكسي يوريا؟** بانتظام ( ) غير منتظم ( ) أبدا ( )
5. **هل يتناول الطفل حمض الفوليك؟** بانتظام ( ) غير منتظم ( ) أبدا ( )
6. **هل لدى الطفل تأمين صحي:**  نعم ( ) لا ( )
7. **هل يذهب الطفل إلى المدرسة:** بانتظام ( ) بشكل غير منتظم ( ) أبدا ( )
8. **إذا لم يذهب الطفل إلى المدرسة / ذهب بشكل غير منتظم ، فلماذا؟** صغر السن ( ) مرض ( ) مالي ( ) ( )

**تقييم عبء مقدمي الرعاية من زاريت (قصير، 12 عنصرا)**

فيما يلي قائمة بالعبارات التي تعكس ما يشعر به الناس أحيانا عند الاعتناء بشخص آخر. بعد قراءة كل عبارة ، حدد عدد المرات التي تشعر فيها بالمشاعر المدرجة عن طريق تدوير الرقم الذي يتوافق بشكل أفضل مع تكرار هذه المشاعر.

|  | **أبدا** | **نادرا** | **أحيانا** | **شكل متكرر** | **دائما تقريبا** |
| --- | --- | --- | --- | --- | --- |
| 1) هل تشعر أنه ليس لديك الوقت الكافي لنفسك؟ | 0 | 1 | 2 | 3 | 4 |
| 2) هل تشعر بالتوتر بين الرعاية والوفاء بالمسؤوليات الأخرى؟ | 0 | 1 | 2 | 3 | 4 |
| 3) هل تشعر بالغضب عندما تكون حول قريبك؟ | 0 | 1 | 2 | 3 | 4 |
| 4) هل تشعر أن قريبك يؤثر على علاقتك بالآخرين بطريقة سلبية؟ | 0 | 1 | 2 | 3 | 4 |
| 5) هل تشعر بالتوتر عندما تكون حول قريبك؟ | 0 | 1 | 2 | 3 | 4 |
| 6) هل تشعر أن صحتك قد عانت بسبب مشاركتك مع قريبك؟ | 0 | 1 | 2 | 3 | 4 |
| 7) هل تشعر أنك لا تتمتع بقدر كبير من الخصوصية كما تريد ، بسبب قريبك؟ | 0 | 1 | 2 | 3 | 4 |
| 8) هل تشعر أن حياتك الاجتماعية لديها  عانت لأنك تعتني بقريبك؟ | 0 | 1 | 2 | 3 | 4 |
| 9) هل تشعر أنك فقدت السيطرة على حياتك منذ مرض قريبك؟ | 0 | 1 | 2 | 3 | 4 |
| 10) هل تشعر بعدم اليقين بشأن ما يجب فعله حيال القريب؟ | 0 | 1 | 2 | 3 | 4 |
| 11) هل تشعر أنه يجب عليك فعل المزيد من أجل قريبك؟ | 0 | 1 | 2 | 3 | 4 |
| 12) هل تشعر أنه يمكنك القيام بعمل أفضل في رعاية قريبك؟ | 0 | 1 | 2 | 3 | 4 |

تعليمات تسجيل الدرجات: إضافة العناصر 1-12 المجموع 1-12 (الحد الأقصى للدرجة = 48)
