# Supplementary material for: Silent struggles: Assessing physical and psychosocial burdens among caregivers of children with sickle cell disease in western Sudan–A cross-sectional study
Source: PLoS One. 2025 Nov 25;20(11):e0336469. doi: 10.1371/journal.pone.0336469 (PMC12646449; doi:10.1371/journal.pone.0336469)
Supplement: S4 File — Software data. (DOCX) [file pone.0336469.s006.docx]

# File 4: Software data

GET

FILE="C:\Users\X415\Documents\Data analysis\Weaam's Data.sav".

DATASET NAME DataSet1 WINDOW=FRONT.

FREQUENCIES VARIABLES=Relationshiptochild Genderofthecaregiver Area Residence Age Maritalstatus M.Education F.Education F.Occupation M.Occupation F.Tribe M.Tribe Income Totalchildren Sicklerchildren Genchild Agechild Diagnosisage Hydroxyurea Folicacid

Healthinsurance School Ifnotothepreviousquestion burdenoncaregiver Genchild2 Agechild2 Diagnosisage2 Hydroxyurea2 Folicacid2 Healthinsurance2 School2 Ifno2 Burden2 Genchild3 Agechild3 Diagnosisage3 Hydroxyurea3 Folicacid3 Healthinsurance3 school3 Ifno3

Burden3 Q1 Q2 Q3 Q4 Q5 Q6 Q7 Q8 Q9 Q10 Q11 Q12 Physical Social Financial Psychological

/ORDER=ANALYSIS.

**Frequencies**

| **Notes** | | |
| --- | --- | --- |
| Output Created | | 05-DEC-2024 13:52:47 |
| Comments | |  |
| Input | Data | C:\Users\X415\Documents\Data analysis\Weaam's Data.sav |
|  | Active Dataset | DataSet1 |
|  | Filter | <none> |
|  | Weight | <none> |
|  | Split File | <none> |
|  | N of Rows in Working Data File | 985 |
| Missing Value Handling | Definition of Missing | User-defined missing values are treated as missing. |
|  | Cases Used | Statistics are based on all cases with valid data. |

| **Notes** | | |
| --- | --- | --- |
| Syntax | | FREQUENCIES VARIABLES=Relationshiptochild Genderofthecaregiver Area Residence Age Maritalstatus M.Education F.Education F.Occupation M.Occupation F.Tribe M.Tribe Income Totalchildren Sicklerchildren Genchild Agechild Diagnosisage Hydroxyurea Folicacid  Healthinsurance School Ifnotothepreviousquestion burdenoncaregiver Genchild2 Agechild2 Diagnosisage2 Hydroxyurea2 Folicacid2 Healthinsurance2 School2 Ifno2 Burden2 Genchild3 Agechild3 Diagnosisage3 Hydroxyurea3 Folicacid3 Healthinsurance3 school3 Ifno3  Burden3 Q1 Q2 Q3 Q4 Q5 Q6 Q7 Q8 Q9 Q10 Q11 Q12 Physical Social Financial Psychological  /ORDER=ANALYSIS. |
| Resources | Processor Time | 00:00:00.02 |
|  | Elapsed Time | 00:00:00.02 |

[DataSet1] C:\Users\X415\Documents\Data analysis\Weaam's Data.sav

| **Statistics** | | | | | | | |
| --- | --- | --- | --- | --- | --- | --- | --- |
|  | | Relationship to child | Gender of the caregiver | Area | Residence | Age | Marital status |
| N | Valid | 123 | 123 | 123 | 123 | 123 | 123 |
|  | Missing | 862 | 862 | 862 | 862 | 862 | 862 |

| **Statistics** | | | | | | | |
| --- | --- | --- | --- | --- | --- | --- | --- |
|  | | M. Education | F. Education | F. Occupation | M. Occupation | F. Tribe | M. Tribe |
| N | Valid | 123 | 123 | 123 | 123 | 123 | 123 |
|  | Missing | 862 | 862 | 862 | 862 | 862 | 862 |

| **Statistics** | | | | | | | |
| --- | --- | --- | --- | --- | --- | --- | --- |
|  | | F. Income | Total children | Sickler children | Gen child | Age child | Diagnosis age |
| N | Valid | 123 | 122 | 123 | 123 | 123 | 123 |
|  | Missing | 862 | 863 | 862 | 862 | 862 | 862 |

| **Statistics** | | | | | | | |
| --- | --- | --- | --- | --- | --- | --- | --- |
|  | | Hydroxyurea | Folic acid | Health insurance | School | If no to the previous question | burden on care giver |
| N | Valid | 123 | 123 | 123 | 123 | 123 | 123 |
|  | Missing | 862 | 862 | 862 | 862 | 862 | 862 |

| **Statistics** | | | | | | |
| --- | --- | --- | --- | --- | --- | --- |
|  | | Gen child 2 | Age child 2 | Diagnosis age 2 | Hydroxyurea 2 | Folic acid 2 |
| N | Valid | 17 | 17 | 17 | 17 | 17 |
|  | Missing | 968 | 968 | 968 | 968 | 968 |

| **Statistics** | | | | | | | |
| --- | --- | --- | --- | --- | --- | --- | --- |
|  | | Health insurance 2 | School 2 | If no 2 | Burden 2 | Gen child 3 | Age child 3 |
| N | Valid | 17 | 17 | 8 | 17 | 1 | 1 |
|  | Missing | 968 | 968 | 977 | 968 | 984 | 984 |

| **Statistics** | | | | | | | |
| --- | --- | --- | --- | --- | --- | --- | --- |
|  | | Diagnosis age 3 | Hydroxyurea 3 | Folic acid 3 | Health insurance 3 | school 3 | If no 3 |
| N | Valid | 1 | 1 | 1 | 1 | 1 | 1 |
|  | Missing | 984 | 984 | 984 | 984 | 984 | 984 |

| **Statistics** | | | | | | | | |
| --- | --- | --- | --- | --- | --- | --- | --- | --- |
|  | | Burden 3 | Q1 | Q2 | Q3 | Q4 | Q5 | Q6 |
| N | Valid | 1 | 123 | 123 | 123 | 123 | 123 | 123 |
|  | Missing | 984 | 862 | 862 | 862 | 862 | 862 | 862 |

| **Statistics** | | | | | | | | |
| --- | --- | --- | --- | --- | --- | --- | --- | --- |
|  | | Q7 | Q8 | Q9 | Q10 | Q11 | Q12 | Physical |
| N | Valid | 123 | 123 | 123 | 123 | 123 | 123 | 123 |
|  | Missing | 862 | 862 | 862 | 862 | 862 | 862 | 862 |

| **Statistics** | | | | |
| --- | --- | --- | --- | --- |
|  | | Social | Financial | Psychological |
| N | Valid | 123 | 123 | 123 |
|  | Missing | 862 | 862 | 862 |

**Frequency Table**

| **Relationship to child** | | | | | |
| --- | --- | --- | --- | --- | --- |
|  | | Frequency | Percent | Valid Percent | Cumulative Percent |
| Valid | Mother | 104 | 10.6 | 84.6 | 84.6 |
|  | Father | 13 | 1.3 | 10.6 | 95.1 |
|  | Sibling | 1 | .1 | .8 | 95.9 |
|  | Aunt | 3 | .3 | 2.4 | 98.4 |
|  | Grandparent | 2 | .2 | 1.6 | 100.0 |
|  | Total | 123 | 12.5 | 100.0 |  |
| Missing | System | 862 | 87.5 |  |  |
| Total | | 985 | 100.0 |  |  |

| **Gender of the caregiver** | | | | | |
| --- | --- | --- | --- | --- | --- |
|  | | Frequency | Percent | Valid Percent | Cumulative Percent |
| Valid | Male | 13 | 1.3 | 10.6 | 10.6 |
|  | Female | 110 | 11.2 | 89.4 | 100.0 |
|  | Total | 123 | 12.5 | 100.0 |  |
| Missing | System | 862 | 87.5 |  |  |
| Total | | 985 | 100.0 |  |  |

| **Area** | | | | | |
| --- | --- | --- | --- | --- | --- |
|  | | Frequency | Percent | Valid Percent | Cumulative Percent |
| Valid | Elobeid | 75 | 7.6 | 61.0 | 61.0 |
|  | Outside Elobeid | 48 | 4.9 | 39.0 | 100.0 |
|  | Total | 123 | 12.5 | 100.0 |  |
| Missing | System | 862 | 87.5 |  |  |
| Total | | 985 | 100.0 |  |  |

| **Residence** | | | | | |
| --- | --- | --- | --- | --- | --- |
|  | | Frequency | Percent | Valid Percent | Cumulative Percent |
| Valid | Rural | 34 | 3.5 | 27.6 | 27.6 |
|  | Urban | 89 | 9.0 | 72.4 | 100.0 |
|  | Total | 123 | 12.5 | 100.0 |  |
| Missing | System | 862 | 87.5 |  |  |
| Total | | 985 | 100.0 |  |  |

| **Age** | | | | | |
| --- | --- | --- | --- | --- | --- |
|  | | Frequency | Percent | Valid Percent | Cumulative Percent |
| Valid | <20 Years | 2 | .2 | 1.6 | 1.6 |
|  | 20-30 Years | 51 | 5.2 | 41.5 | 43.1 |
|  | 31-40 Years | 44 | 4.5 | 35.8 | 78.9 |
|  | >40 Years | 26 | 2.6 | 21.1 | 100.0 |
|  | Total | 123 | 12.5 | 100.0 |  |
| Missing | System | 862 | 87.5 |  |  |
| Total | | 985 | 100.0 |  |  |

| **Marital status** | | | | | |
| --- | --- | --- | --- | --- | --- |
|  | | Frequency | Percent | Valid Percent | Cumulative Percent |
| Valid | Single | 2 | .2 | 1.6 | 1.6 |
|  | Married | 112 | 11.4 | 91.1 | 92.7 |
|  | Divorced | 3 | .3 | 2.4 | 95.1 |
|  | Widowed | 6 | .6 | 4.9 | 100.0 |
|  | Total | 123 | 12.5 | 100.0 |  |
| Missing | System | 862 | 87.5 |  |  |
| Total | | 985 | 100.0 |  |  |

| **M. Education** | | | | | |
| --- | --- | --- | --- | --- | --- |
|  | | Frequency | Percent | Valid Percent | Cumulative Percent |
| Valid | Illiteracy | 9 | .9 | 7.3 | 7.3 |
|  | Khalwa | 12 | 1.2 | 9.8 | 17.1 |
|  | Primary | 46 | 4.7 | 37.4 | 54.5 |
|  | Secondary | 33 | 3.4 | 26.8 | 81.3 |
|  | University | 23 | 2.3 | 18.7 | 100.0 |
|  | Total | 123 | 12.5 | 100.0 |  |
| Missing | System | 862 | 87.5 |  |  |
| Total | | 985 | 100.0 |  |  |

| **F. Education** | | | | | |
| --- | --- | --- | --- | --- | --- |
|  | | Frequency | Percent | Valid Percent | Cumulative Percent |
| Valid | Illiteracy | 13 | 1.3 | 10.6 | 10.6 |
|  | Khalwa | 10 | 1.0 | 8.1 | 18.7 |
|  | Primary | 49 | 5.0 | 39.8 | 58.5 |
|  | Secondary | 34 | 3.5 | 27.6 | 86.2 |
|  | University | 15 | 1.5 | 12.2 | 98.4 |
|  | Postgraduate | 2 | .2 | 1.6 | 100.0 |
|  | Total | 123 | 12.5 | 100.0 |  |
| Missing | System | 862 | 87.5 |  |  |
| Total | | 985 | 100.0 |  |  |

| **F. Occupation** | | | | | |
| --- | --- | --- | --- | --- | --- |
|  | | Frequency | Percent | Valid Percent | Cumulative Percent |
| Valid | Employee | 23 | 2.3 | 18.7 | 18.7 |
|  | Freework | 95 | 9.6 | 77.2 | 95.9 |
|  | Unemployed | 5 | .5 | 4.1 | 100.0 |
|  | Total | 123 | 12.5 | 100.0 |  |
| Missing | System | 862 | 87.5 |  |  |
| Total | | 985 | 100.0 |  |  |

| **M. Occupation** | | | | | |
| --- | --- | --- | --- | --- | --- |
|  | | Frequency | Percent | Valid Percent | Cumulative Percent |
| Valid | Employee | 12 | 1.2 | 9.8 | 9.8 |
|  | Housewife | 104 | 10.6 | 84.6 | 94.3 |
|  | Worker | 7 | .7 | 5.7 | 100.0 |
|  | Total | 123 | 12.5 | 100.0 |  |
| Missing | System | 862 | 87.5 |  |  |
| Total | | 985 | 100.0 |  |  |

| **F. Tribe** | | | | | |
| --- | --- | --- | --- | --- | --- |
|  | | Frequency | Percent | Valid Percent | Cumulative Percent |
| Valid | Bagara | 26 | 2.6 | 21.1 | 21.1 |
|  | Bideriya | 18 | 1.8 | 14.6 | 35.8 |
|  | Gawam'a | 4 | .4 | 3.3 | 39.0 |
|  | Falata | 19 | 1.9 | 15.4 | 54.5 |
|  | Bargo | 6 | .6 | 4.9 | 59.3 |
|  | Dar Hamid | 7 | .7 | 5.7 | 65.0 |
|  | Others | 43 | 4.4 | 35.0 | 100.0 |
|  | Total | 123 | 12.5 | 100.0 |  |
| Missing | System | 862 | 87.5 |  |  |
| Total | | 985 | 100.0 |  |  |

| **M. Tribe** | | | | | |
| --- | --- | --- | --- | --- | --- |
|  | | Frequency | Percent | Valid Percent | Cumulative Percent |
| Valid | Bagara | 20 | 2.0 | 16.3 | 16.3 |
|  | Bideriya | 15 | 1.5 | 12.2 | 28.5 |
|  | Gawam'a | 6 | .6 | 4.9 | 33.3 |
|  | Falata | 22 | 2.2 | 17.9 | 51.2 |
|  | Bargo | 6 | .6 | 4.9 | 56.1 |
|  | Dar Hamid | 10 | 1.0 | 8.1 | 64.2 |
|  | Others | 44 | 4.5 | 35.8 | 100.0 |
|  | Total | 123 | 12.5 | 100.0 |  |
| Missing | System | 862 | 87.5 |  |  |
| Total | | 985 | 100.0 |  |  |

| **F. Income** | | | | | |
| --- | --- | --- | --- | --- | --- |
|  | | Frequency | Percent | Valid Percent | Cumulative Percent |
| Valid | <50,000 SDG | 53 | 5.4 | 43.1 | 43.1 |
|  | 50,000-100,000 SDG | 49 | 5.0 | 39.8 | 82.9 |
|  | 100,000-200,000 SDG | 12 | 1.2 | 9.8 | 92.7 |
|  | >200,000 SDG | 9 | .9 | 7.3 | 100.0 |
|  | Total | 123 | 12.5 | 100.0 |  |
| Missing | System | 862 | 87.5 |  |  |
| Total | | 985 | 100.0 |  |  |

| **Total children** | | | | | |
| --- | --- | --- | --- | --- | --- |
|  | | Frequency | Percent | Valid Percent | Cumulative Percent |
| Valid | 1 | 11 | 1.1 | 9.0 | 9.0 |
|  | 2 | 23 | 2.3 | 18.9 | 27.9 |
|  | 3 | 21 | 2.1 | 17.2 | 45.1 |
|  | >3 | 67 | 6.8 | 54.9 | 100.0 |
|  | Total | 122 | 12.4 | 100.0 |  |
| Missing | System | 863 | 87.6 |  |  |
| Total | | 985 | 100.0 |  |  |

| **Sickler children** | | | | | |
| --- | --- | --- | --- | --- | --- |
|  | | Frequency | Percent | Valid Percent | Cumulative Percent |
| Valid | 1 | 101 | 10.3 | 82.1 | 82.1 |
|  | 2 | 20 | 2.0 | 16.3 | 98.4 |
|  | 3 | 1 | .1 | .8 | 99.2 |
|  | >3 | 1 | .1 | .8 | 100.0 |
|  | Total | 123 | 12.5 | 100.0 |  |
| Missing | System | 862 | 87.5 |  |  |
| Total | | 985 | 100.0 |  |  |

| **Gen child** | | | | | |
| --- | --- | --- | --- | --- | --- |
|  | | Frequency | Percent | Valid Percent | Cumulative Percent |
| Valid | Male | 64 | 6.5 | 52.0 | 52.0 |
|  | Female | 59 | 6.0 | 48.0 | 100.0 |
|  | Total | 123 | 12.5 | 100.0 |  |
| Missing | System | 862 | 87.5 |  |  |
| Total | | 985 | 100.0 |  |  |

| **Age child** | | | | | |
| --- | --- | --- | --- | --- | --- |
|  | | Frequency | Percent | Valid Percent | Cumulative Percent |
| Valid | 0-4 | 46 | 4.7 | 37.4 | 37.4 |
|  | 5-9 | 40 | 4.1 | 32.5 | 69.9 |
|  | 10-13 | 29 | 2.9 | 23.6 | 93.5 |
|  | 14-18 | 8 | .8 | 6.5 | 100.0 |
|  | Total | 123 | 12.5 | 100.0 |  |
| Missing | System | 862 | 87.5 |  |  |
| Total | | 985 | 100.0 |  |  |

| **Diagnosis age** | | | | | |
| --- | --- | --- | --- | --- | --- |
|  | | Frequency | Percent | Valid Percent | Cumulative Percent |
| Valid | <6 Months | 27 | 2.7 | 22.0 | 22.0 |
|  | 6-12 Months | 65 | 6.6 | 52.8 | 74.8 |
|  | >12 Months | 31 | 3.1 | 25.2 | 100.0 |
|  | Total | 123 | 12.5 | 100.0 |  |
| Missing | System | 862 | 87.5 |  |  |
| Total | | 985 | 100.0 |  |  |

| **Hydroxyurea** | | | | | |
| --- | --- | --- | --- | --- | --- |
|  | | Frequency | Percent | Valid Percent | Cumulative Percent |
| Valid | Regularly | 106 | 10.8 | 86.2 | 86.2 |
|  | Irregularly | 5 | .5 | 4.1 | 90.2 |
|  | Never | 12 | 1.2 | 9.8 | 100.0 |
|  | Total | 123 | 12.5 | 100.0 |  |
| Missing | System | 862 | 87.5 |  |  |
| Total | | 985 | 100.0 |  |  |

| **Folic acid** | | | | | |
| --- | --- | --- | --- | --- | --- |
|  | | Frequency | Percent | Valid Percent | Cumulative Percent |
| Valid | Regularly | 120 | 12.2 | 97.6 | 97.6 |
|  | Irregularly | 1 | .1 | .8 | 98.4 |
|  | Never | 2 | .2 | 1.6 | 100.0 |
|  | Total | 123 | 12.5 | 100.0 |  |
| Missing | System | 862 | 87.5 |  |  |
| Total | | 985 | 100.0 |  |  |

| **Health insurance** | | | | | |
| --- | --- | --- | --- | --- | --- |
|  | | Frequency | Percent | Valid Percent | Cumulative Percent |
| Valid | Yes | 88 | 8.9 | 71.5 | 71.5 |
|  | No | 35 | 3.6 | 28.5 | 100.0 |
|  | Total | 123 | 12.5 | 100.0 |  |
| Missing | System | 862 | 87.5 |  |  |
| Total | | 985 | 100.0 |  |  |

| **School** | | | | | |
| --- | --- | --- | --- | --- | --- |
|  | | Frequency | Percent | Valid Percent | Cumulative Percent |
| Valid | Regularly | 39 | 4.0 | 31.7 | 31.7 |
|  | Irregularly | 14 | 1.4 | 11.4 | 43.1 |
|  | Never | 70 | 7.1 | 56.9 | 100.0 |
|  | Total | 123 | 12.5 | 100.0 |  |
| Missing | System | 862 | 87.5 |  |  |
| Total | | 985 | 100.0 |  |  |

| **If no to the previous question** | | | | | |
| --- | --- | --- | --- | --- | --- |
|  | | Frequency | Percent | Valid Percent | Cumulative Percent |
| Valid | Young age | 51 | 5.2 | 41.5 | 41.5 |
|  | Illness | 30 | 3.0 | 24.4 | 65.9 |
|  | Financial | 2 | .2 | 1.6 | 67.5 |
|  | Yes | 40 | 4.1 | 32.5 | 100.0 |
|  | Total | 123 | 12.5 | 100.0 |  |
| Missing | System | 862 | 87.5 |  |  |
| Total | | 985 | 100.0 |  |  |

| **burden on care giver** | | | | | |
| --- | --- | --- | --- | --- | --- |
|  | | Frequency | Percent | Valid Percent | Cumulative Percent |
| Valid | Physical | 15 | 1.5 | 12.2 | 12.2 |
|  | Social | 14 | 1.4 | 11.4 | 23.6 |
|  | Financial | 45 | 4.6 | 36.6 | 60.2 |
|  | Psychological | 18 | 1.8 | 14.6 | 74.8 |
|  | None | 9 | .9 | 7.3 | 82.1 |
|  | All | 11 | 1.1 | 8.9 | 91.1 |
|  | Financial and Psychological | 9 | .9 | 7.3 | 98.4 |
|  | Financial and Physical | 2 | .2 | 1.6 | 100.0 |
|  | Total | 123 | 12.5 | 100.0 |  |
| Missing | System | 862 | 87.5 |  |  |
| Total | | 985 | 100.0 |  |  |

| **Gen child 2** | | | | | |
| --- | --- | --- | --- | --- | --- |
|  | | Frequency | Percent | Valid Percent | Cumulative Percent |
| Valid | Male | 6 | .6 | 35.3 | 35.3 |
|  | Female | 11 | 1.1 | 64.7 | 100.0 |
|  | Total | 17 | 1.7 | 100.0 |  |
| Missing | 99 | 106 | 10.8 |  |  |
|  | System | 862 | 87.5 |  |  |
|  | Total | 968 | 98.3 |  |  |
| Total | | 985 | 100.0 |  |  |

| **Age child 2** | | | | | |
| --- | --- | --- | --- | --- | --- |
|  | | Frequency | Percent | Valid Percent | Cumulative Percent |
| Valid | 0-4 | 3 | .3 | 17.6 | 17.6 |
|  | 5-9 | 6 | .6 | 35.3 | 52.9 |
|  | 10-13 | 3 | .3 | 17.6 | 70.6 |
|  | 14-18 | 5 | .5 | 29.4 | 100.0 |
|  | Total | 17 | 1.7 | 100.0 |  |
| Missing | 99 | 106 | 10.8 |  |  |
|  | System | 862 | 87.5 |  |  |
|  | Total | 968 | 98.3 |  |  |
| Total | | 985 | 100.0 |  |  |

| **Diagnosis age 2** | | | | | |
| --- | --- | --- | --- | --- | --- |
|  | | Frequency | Percent | Valid Percent | Cumulative Percent |
| Valid | <6 Months | 6 | .6 | 35.3 | 35.3 |
|  | 6-12 Months | 7 | .7 | 41.2 | 76.5 |
|  | >12 Months | 4 | .4 | 23.5 | 100.0 |
|  | Total | 17 | 1.7 | 100.0 |  |
| Missing | 99 | 106 | 10.8 |  |  |
|  | System | 862 | 87.5 |  |  |
|  | Total | 968 | 98.3 |  |  |
| Total | | 985 | 100.0 |  |  |

| **Hydroxyurea 2** | | | | | |
| --- | --- | --- | --- | --- | --- |
|  | | Frequency | Percent | Valid Percent | Cumulative Percent |
| Valid | Regularly | 15 | 1.5 | 88.2 | 88.2 |
|  | Never | 2 | .2 | 11.8 | 100.0 |
|  | Total | 17 | 1.7 | 100.0 |  |
| Missing | 99 | 106 | 10.8 |  |  |
|  | System | 862 | 87.5 |  |  |
|  | Total | 968 | 98.3 |  |  |
| Total | | 985 | 100.0 |  |  |

| **Folic acid 2** | | | | | |
| --- | --- | --- | --- | --- | --- |
|  | | Frequency | Percent | Valid Percent | Cumulative Percent |
| Valid | Regularly | 17 | 1.7 | 100.0 | 100.0 |
| Missing | 99 | 106 | 10.8 |  |  |
|  | System | 862 | 87.5 |  |  |
|  | Total | 968 | 98.3 |  |  |
| Total | | 985 | 100.0 |  |  |

| **Health insurance 2** | | | | | |
| --- | --- | --- | --- | --- | --- |
|  | | Frequency | Percent | Valid Percent | Cumulative Percent |
| Valid | Yes | 13 | 1.3 | 76.5 | 76.5 |
|  | No | 4 | .4 | 23.5 | 100.0 |
|  | Total | 17 | 1.7 | 100.0 |  |
| Missing | 99 | 106 | 10.8 |  |  |
|  | System | 862 | 87.5 |  |  |
|  | Total | 968 | 98.3 |  |  |
| Total | | 985 | 100.0 |  |  |

| **School 2** | | | | | |
| --- | --- | --- | --- | --- | --- |
|  | | Frequency | Percent | Valid Percent | Cumulative Percent |
| Valid | Regularly | 9 | .9 | 52.9 | 52.9 |
|  | Irregularly | 3 | .3 | 17.6 | 70.6 |
|  | Never | 5 | .5 | 29.4 | 100.0 |
|  | Total | 17 | 1.7 | 100.0 |  |
| Missing | 99 | 106 | 10.8 |  |  |
|  | System | 862 | 87.5 |  |  |
|  | Total | 968 | 98.3 |  |  |
| Total | | 985 | 100.0 |  |  |

| **If no 2** | | | | | |
| --- | --- | --- | --- | --- | --- |
|  | | Frequency | Percent | Valid Percent | Cumulative Percent |
| Valid | Young age | 3 | .3 | 37.5 | 37.5 |
|  | Illness | 4 | .4 | 50.0 | 87.5 |
|  | Yes | 1 | .1 | 12.5 | 100.0 |
|  | Total | 8 | .8 | 100.0 |  |
| Missing | 99 | 115 | 11.7 |  |  |
|  | System | 862 | 87.5 |  |  |
|  | Total | 977 | 99.2 |  |  |
| Total | | 985 | 100.0 |  |  |

| **Burden 2** | | | | | |
| --- | --- | --- | --- | --- | --- |
|  | | Frequency | Percent | Valid Percent | Cumulative Percent |
| Valid | Physical | 4 | .4 | 23.5 | 23.5 |
|  | Social | 4 | .4 | 23.5 | 47.1 |
|  | Financial | 6 | .6 | 35.3 | 82.4 |
|  | Psychological | 2 | .2 | 11.8 | 94.1 |
|  | Financial and Physical | 1 | .1 | 5.9 | 100.0 |
|  | Total | 17 | 1.7 | 100.0 |  |
| Missing | 99 | 106 | 10.8 |  |  |
|  | System | 862 | 87.5 |  |  |
|  | Total | 968 | 98.3 |  |  |
| Total | | 985 | 100.0 |  |  |

| **Gen child 3** | | | | | |
| --- | --- | --- | --- | --- | --- |
|  | | Frequency | Percent | Valid Percent | Cumulative Percent |
| Valid | Female | 1 | .1 | 100.0 | 100.0 |
| Missing | 99 | 122 | 12.4 |  |  |
|  | System | 862 | 87.5 |  |  |
|  | Total | 984 | 99.9 |  |  |
| Total | | 985 | 100.0 |  |  |

| **Age child 3** | | | | | |
| --- | --- | --- | --- | --- | --- |
|  | | Frequency | Percent | Valid Percent | Cumulative Percent |
| Valid | 0-4 | 1 | .1 | 100.0 | 100.0 |
| Missing | 99 | 122 | 12.4 |  |  |
|  | System | 862 | 87.5 |  |  |
|  | Total | 984 | 99.9 |  |  |
| Total | | 985 | 100.0 |  |  |

| **Diagnosis age 3** | | | | | |
| --- | --- | --- | --- | --- | --- |
|  | | Frequency | Percent | Valid Percent | Cumulative Percent |
| Valid | <6 Months | 1 | .1 | 100.0 | 100.0 |
| Missing | 99 | 122 | 12.4 |  |  |
|  | System | 862 | 87.5 |  |  |
|  | Total | 984 | 99.9 |  |  |
| Total | | 985 | 100.0 |  |  |

| **Hydroxyurea 3** | | | | | |
| --- | --- | --- | --- | --- | --- |
|  | | Frequency | Percent | Valid Percent | Cumulative Percent |
| Valid | Regularly | 1 | .1 | 100.0 | 100.0 |
| Missing | 99 | 122 | 12.4 |  |  |
|  | System | 862 | 87.5 |  |  |
|  | Total | 984 | 99.9 |  |  |
| Total | | 985 | 100.0 |  |  |

| **Folic acid 3** | | | | | |
| --- | --- | --- | --- | --- | --- |
|  | | Frequency | Percent | Valid Percent | Cumulative Percent |
| Valid | Regularly | 1 | .1 | 100.0 | 100.0 |
| Missing | 99 | 122 | 12.4 |  |  |
|  | System | 862 | 87.5 |  |  |
|  | Total | 984 | 99.9 |  |  |
| Total | | 985 | 100.0 |  |  |

| **Health insurance 3** | | | | | |
| --- | --- | --- | --- | --- | --- |
|  | | Frequency | Percent | Valid Percent | Cumulative Percent |
| Valid | No | 1 | .1 | 100.0 | 100.0 |
| Missing | 99 | 122 | 12.4 |  |  |
|  | System | 862 | 87.5 |  |  |
|  | Total | 984 | 99.9 |  |  |
| Total | | 985 | 100.0 |  |  |

| **school 3** | | | | | |
| --- | --- | --- | --- | --- | --- |
|  | | Frequency | Percent | Valid Percent | Cumulative Percent |
| Valid | Never | 1 | .1 | 100.0 | 100.0 |
| Missing | 99 | 122 | 12.4 |  |  |
|  | System | 862 | 87.5 |  |  |
|  | Total | 984 | 99.9 |  |  |
| Total | | 985 | 100.0 |  |  |

| **If no 3** | | | | | |
| --- | --- | --- | --- | --- | --- |
|  | | Frequency | Percent | Valid Percent | Cumulative Percent |
| Valid | Young age | 1 | .1 | 100.0 | 100.0 |
| Missing | 99 | 122 | 12.4 |  |  |
|  | System | 862 | 87.5 |  |  |
|  | Total | 984 | 99.9 |  |  |
| Total | | 985 | 100.0 |  |  |

| **Burden 3** | | | | | |
| --- | --- | --- | --- | --- | --- |
|  | | Frequency | Percent | Valid Percent | Cumulative Percent |
| Valid | Financial | 1 | .1 | 100.0 | 100.0 |
| Missing | 99 | 122 | 12.4 |  |  |
|  | System | 862 | 87.5 |  |  |
|  | Total | 984 | 99.9 |  |  |
| Total | | 985 | 100.0 |  |  |

| **Q1** | | | | | |
| --- | --- | --- | --- | --- | --- |
|  | | Frequency | Percent | Valid Percent | Cumulative Percent |
| Valid | Never | 66 | 6.7 | 53.7 | 53.7 |
|  | Rarely | 5 | .5 | 4.1 | 57.7 |
|  | Sometimes | 25 | 2.5 | 20.3 | 78.0 |
|  | Frequently | 4 | .4 | 3.3 | 81.3 |
|  | Nearly always | 23 | 2.3 | 18.7 | 100.0 |
|  | Total | 123 | 12.5 | 100.0 |  |
| Missing | System | 862 | 87.5 |  |  |
| Total | | 985 | 100.0 |  |  |

| **Q2** | | | | | |
| --- | --- | --- | --- | --- | --- |
|  | | Frequency | Percent | Valid Percent | Cumulative Percent |
| Valid | Never | 61 | 6.2 | 49.6 | 49.6 |
|  | Rarely | 13 | 1.3 | 10.6 | 60.2 |
|  | Sometimes | 20 | 2.0 | 16.3 | 76.4 |
|  | Frequently | 10 | 1.0 | 8.1 | 84.6 |
|  | Nearly always | 19 | 1.9 | 15.4 | 100.0 |
|  | Total | 123 | 12.5 | 100.0 |  |
| Missing | System | 862 | 87.5 |  |  |
| Total | | 985 | 100.0 |  |  |

| **Q3** | | | | | |
| --- | --- | --- | --- | --- | --- |
|  | | Frequency | Percent | Valid Percent | Cumulative Percent |
| Valid | Never | 88 | 8.9 | 71.5 | 71.5 |
|  | Rarely | 11 | 1.1 | 8.9 | 80.5 |
|  | Sometimes | 18 | 1.8 | 14.6 | 95.1 |
|  | Frequently | 3 | .3 | 2.4 | 97.6 |
|  | Nearly always | 3 | .3 | 2.4 | 100.0 |
|  | Total | 123 | 12.5 | 100.0 |  |
| Missing | System | 862 | 87.5 |  |  |
| Total | | 985 | 100.0 |  |  |

| **Q4** | | | | | |
| --- | --- | --- | --- | --- | --- |
|  | | Frequency | Percent | Valid Percent | Cumulative Percent |
| Valid | Never | 96 | 9.7 | 78.0 | 78.0 |
|  | Rarely | 3 | .3 | 2.4 | 80.5 |
|  | Sometimes | 11 | 1.1 | 8.9 | 89.4 |
|  | Frequently | 5 | .5 | 4.1 | 93.5 |
|  | Nearly always | 8 | .8 | 6.5 | 100.0 |
|  | Total | 123 | 12.5 | 100.0 |  |
| Missing | System | 862 | 87.5 |  |  |
| Total | | 985 | 100.0 |  |  |

| **Q5** | | | | | |
| --- | --- | --- | --- | --- | --- |
|  | | Frequency | Percent | Valid Percent | Cumulative Percent |
| Valid | Never | 78 | 7.9 | 63.4 | 63.4 |
|  | Rarely | 10 | 1.0 | 8.1 | 71.5 |
|  | Sometimes | 26 | 2.6 | 21.1 | 92.7 |
|  | Frequently | 1 | .1 | .8 | 93.5 |
|  | Nearly always | 8 | .8 | 6.5 | 100.0 |
|  | Total | 123 | 12.5 | 100.0 |  |
| Missing | System | 862 | 87.5 |  |  |
| Total | | 985 | 100.0 |  |  |

| **Q6** | | | | | |
| --- | --- | --- | --- | --- | --- |
|  | | Frequency | Percent | Valid Percent | Cumulative Percent |
| Valid | Never | 76 | 7.7 | 61.8 | 61.8 |
|  | Rarely | 12 | 1.2 | 9.8 | 71.5 |
|  | Sometimes | 12 | 1.2 | 9.8 | 81.3 |
|  | Frequently | 6 | .6 | 4.9 | 86.2 |
|  | Nearly always | 17 | 1.7 | 13.8 | 100.0 |
|  | Total | 123 | 12.5 | 100.0 |  |
| Missing | System | 862 | 87.5 |  |  |
| Total | | 985 | 100.0 |  |  |

| **Q7** | | | | | |
| --- | --- | --- | --- | --- | --- |
|  | | Frequency | Percent | Valid Percent | Cumulative Percent |
| Valid | Never | 84 | 8.5 | 68.3 | 68.3 |
|  | Rarely | 8 | .8 | 6.5 | 74.8 |
|  | Sometimes | 16 | 1.6 | 13.0 | 87.8 |
|  | Frequently | 1 | .1 | .8 | 88.6 |
|  | Nearly always | 14 | 1.4 | 11.4 | 100.0 |
|  | Total | 123 | 12.5 | 100.0 |  |
| Missing | System | 862 | 87.5 |  |  |
| Total | | 985 | 100.0 |  |  |

| **Q8** | | | | | |
| --- | --- | --- | --- | --- | --- |
|  | | Frequency | Percent | Valid Percent | Cumulative Percent |
| Valid | Never | 80 | 8.1 | 65.0 | 65.0 |
|  | Rarely | 6 | .6 | 4.9 | 69.9 |
|  | Sometimes | 19 | 1.9 | 15.4 | 85.4 |
|  | Frequently | 3 | .3 | 2.4 | 87.8 |
|  | Nearly always | 15 | 1.5 | 12.2 | 100.0 |
|  | Total | 123 | 12.5 | 100.0 |  |
| Missing | System | 862 | 87.5 |  |  |
| Total | | 985 | 100.0 |  |  |

| **Q9** | | | | | |
| --- | --- | --- | --- | --- | --- |
|  | | Frequency | Percent | Valid Percent | Cumulative Percent |
| Valid | Never | 86 | 8.7 | 69.9 | 69.9 |
|  | Rarely | 5 | .5 | 4.1 | 74.0 |
|  | Sometimes | 21 | 2.1 | 17.1 | 91.1 |
|  | Frequently | 2 | .2 | 1.6 | 92.7 |
|  | Nearly always | 9 | .9 | 7.3 | 100.0 |
|  | Total | 123 | 12.5 | 100.0 |  |
| Missing | System | 862 | 87.5 |  |  |
| Total | | 985 | 100.0 |  |  |

| **Q10** | | | | | |
| --- | --- | --- | --- | --- | --- |
|  | | Frequency | Percent | Valid Percent | Cumulative Percent |
| Valid | Never | 110 | 11.2 | 89.4 | 89.4 |
|  | Rarely | 1 | .1 | .8 | 90.2 |
|  | Sometimes | 3 | .3 | 2.4 | 92.7 |
|  | Frequently | 1 | .1 | .8 | 93.5 |
|  | Nearly always | 8 | .8 | 6.5 | 100.0 |
|  | Total | 123 | 12.5 | 100.0 |  |
| Missing | System | 862 | 87.5 |  |  |
| Total | | 985 | 100.0 |  |  |

| **Q11** | | | | | |
| --- | --- | --- | --- | --- | --- |
|  | | Frequency | Percent | Valid Percent | Cumulative Percent |
| Valid | Never | 16 | 1.6 | 13.0 | 13.0 |
|  | Rarely | 1 | .1 | .8 | 13.8 |
|  | Sometimes | 7 | .7 | 5.7 | 19.5 |
|  | Frequently | 3 | .3 | 2.4 | 22.0 |
|  | Nearly always | 96 | 9.7 | 78.0 | 100.0 |
|  | Total | 123 | 12.5 | 100.0 |  |
| Missing | System | 862 | 87.5 |  |  |
| Total | | 985 | 100.0 |  |  |

| **Q12** | | | | | |
| --- | --- | --- | --- | --- | --- |
|  | | Frequency | Percent | Valid Percent | Cumulative Percent |
| Valid | Never | 49 | 5.0 | 39.8 | 39.8 |
|  | Rarely | 1 | .1 | .8 | 40.7 |
|  | Sometimes | 2 | .2 | 1.6 | 42.3 |
|  | Nearly always | 71 | 7.2 | 57.7 | 100.0 |
|  | Total | 123 | 12.5 | 100.0 |  |
| Missing | System | 862 | 87.5 |  |  |
| Total | | 985 | 100.0 |  |  |

| **Physical** | | | | | |
| --- | --- | --- | --- | --- | --- |
|  | | Frequency | Percent | Valid Percent | Cumulative Percent |
| Valid | No | 95 | 9.6 | 77.2 | 77.2 |
|  | Yes | 28 | 2.8 | 22.8 | 100.0 |
|  | Total | 123 | 12.5 | 100.0 |  |
| Missing | System | 862 | 87.5 |  |  |
| Total | | 985 | 100.0 |  |  |

| **Social** | | | | | |
| --- | --- | --- | --- | --- | --- |
|  | | Frequency | Percent | Valid Percent | Cumulative Percent |
| Valid | No | 98 | 9.9 | 79.7 | 79.7 |
|  | Yes | 25 | 2.5 | 20.3 | 100.0 |
|  | Total | 123 | 12.5 | 100.0 |  |
| Missing | System | 862 | 87.5 |  |  |
| Total | | 985 | 100.0 |  |  |

| **Financial** | | | | | |
| --- | --- | --- | --- | --- | --- |
|  | | Frequency | Percent | Valid Percent | Cumulative Percent |
| Valid | No | 56 | 5.7 | 45.5 | 45.5 |
|  | Yes | 67 | 6.8 | 54.5 | 100.0 |
|  | Total | 123 | 12.5 | 100.0 |  |
| Missing | System | 862 | 87.5 |  |  |
| Total | | 985 | 100.0 |  |  |

| **Psychological** | | | | | |
| --- | --- | --- | --- | --- | --- |
|  | | Frequency | Percent | Valid Percent | Cumulative Percent |
| Valid | No | 85 | 8.6 | 69.1 | 69.1 |
|  | Yes | 38 | 3.9 | 30.9 | 100.0 |
|  | Total | 123 | 12.5 | 100.0 |  |
| Missing | System | 862 | 87.5 |  |  |
| Total | | 985 | 100.0 |  |  |

NPAR TESTS

/M-W= TotalZarit BY Genderofthecaregiver(1 2)

/MISSING ANALYSIS.

**NPar Tests**

| **Notes** | | |
| --- | --- | --- |
| Output Created | | 05-DEC-2024 13:59:21 |
| Comments | |  |
| Input | Data | C:\Users\X415\Documents\Data analysis\Weaam's Data.sav |
|  | Active Dataset | DataSet1 |
|  | Filter | <none> |
|  | Weight | <none> |
|  | Split File | <none> |
|  | N of Rows in Working Data File | 985 |
| Missing Value Handling | Definition of Missing | User-defined missing values are treated as missing. |
|  | Cases Used | Statistics for each test are based on all cases with valid data for the variable(s) used in that test. |
| Syntax | | NPAR TESTS  /M-W= TotalZarit BY Genderofthecaregiver(1 2)  /MISSING ANALYSIS. |
| Resources | Processor Time | 00:00:00.00 |
|  | Elapsed Time | 00:00:00.00 |
|  | Number of Cases Allowed^a^ | 112347 |

| a. Based on availability of workspace memory. |
| --- |

[DataSet1] C:\Users\X415\Documents\Data analysis\Weaam's Data.sav

**Mann-Whitney Test**

| **Ranks** | | | | |
| --- | --- | --- | --- | --- |
|  | Gender of the caregiver | N | Mean Rank | Sum of Ranks |
| Total Zarit | Male | 13 | 63.62 | 827.00 |
|  | Female | 110 | 61.81 | 6799.00 |
|  | Total | 123 |  |  |

| **Test Statistics^a^** | |
| --- | --- |
|  | Total Zarit |
| Mann-Whitney U | 694.000 |
| Wilcoxon W | 6799.000 |
| Z | -.173 |
| Asymp. Sig. (2-tailed) | .862 |

| a. Grouping Variable: Gender of the caregiver |
| --- |

NPAR TESTS

/M-W= TotalZarit BY Area(1 2)

/MISSING ANALYSIS.

**NPar Tests**

| **Notes** | | |
| --- | --- | --- |
| Output Created | | 05-DEC-2024 14:12:00 |
| Comments | |  |
| Input | Data | C:\Users\X415\Documents\Data analysis\Weaam's Data.sav |
|  | Active Dataset | DataSet1 |
|  | Filter | <none> |
|  | Weight | <none> |
|  | Split File | <none> |
|  | N of Rows in Working Data File | 985 |
| Missing Value Handling | Definition of Missing | User-defined missing values are treated as missing. |
|  | Cases Used | Statistics for each test are based on all cases with valid data for the variable(s) used in that test. |
| Syntax | | NPAR TESTS  /M-W= TotalZarit BY Area(1 2)  /MISSING ANALYSIS. |
| Resources | Processor Time | 00:00:00.00 |
|  | Elapsed Time | 00:00:00.00 |
|  | Number of Cases Allowed^a^ | 112347 |

| a. Based on availability of workspace memory. |
| --- |

[DataSet1] C:\Users\X415\Documents\Data analysis\Weaam's Data.sav

**Mann-Whitney Test**

| **Ranks** | | | | |
| --- | --- | --- | --- | --- |
|  | Area | N | Mean Rank | Sum of Ranks |
| Total Zarit | Elobeid | 75 | 56.37 | 4228.00 |
|  | Outside Elobeid | 48 | 70.79 | 3398.00 |
|  | Total | 123 |  |  |

| **Test Statistics^a^** | |
| --- | --- |
|  | Total Zarit |
| Mann-Whitney U | 1378.000 |
| Wilcoxon W | 4228.000 |
| Z | -2.195 |
| Asymp. Sig. (2-tailed) | .028 |

| a. Grouping Variable: Area |
| --- |

NPAR TESTS

/M-W= TotalZarit BY Residence(1 2)

/MISSING ANALYSIS.

**NPar Tests**

| **Notes** | | |
| --- | --- | --- |
| Output Created | | 05-DEC-2024 14:45:55 |
| Comments | |  |
| Input | Data | C:\Users\X415\Documents\Data analysis\Weaam's Data.sav |
|  | Active Dataset | DataSet1 |
|  | Filter | <none> |
|  | Weight | <none> |
|  | Split File | <none> |
|  | N of Rows in Working Data File | 985 |
| Missing Value Handling | Definition of Missing | User-defined missing values are treated as missing. |
|  | Cases Used | Statistics for each test are based on all cases with valid data for the variable(s) used in that test. |
| Syntax | | NPAR TESTS  /M-W= TotalZarit BY Residence(1 2)  /MISSING ANALYSIS. |
| Resources | Processor Time | 00:00:00.00 |
|  | Elapsed Time | 00:00:00.00 |
|  | Number of Cases Allowed^a^ | 112347 |

| a. Based on availability of workspace memory. |
| --- |

[DataSet1] C:\Users\X415\Documents\Data analysis\Weaam's Data.sav

**Mann-Whitney Test**

| **Ranks** | | | | |
| --- | --- | --- | --- | --- |
|  | Residence | N | Mean Rank | Sum of Ranks |
| Total Zarit | Rural | 34 | 71.85 | 2443.00 |
|  | Urban | 89 | 58.24 | 5183.00 |
|  | Total | 123 |  |  |

| **Test Statistics^a^** | |
| --- | --- |
|  | Total Zarit |
| Mann-Whitney U | 1178.000 |
| Wilcoxon W | 5183.000 |
| Z | -1.901 |
| Asymp. Sig. (2-tailed) | .057 |

| a. Grouping Variable: Residence |
| --- |

NPAR TESTS

/M-W= TotalZarit BY Healthinsurance(1 2)

/MISSING ANALYSIS.

**NPar Tests**

| **Notes** | | |
| --- | --- | --- |
| Output Created | | 05-DEC-2024 14:48:00 |
| Comments | |  |
| Input | Data | C:\Users\X415\Documents\Data analysis\Weaam's Data.sav |
|  | Active Dataset | DataSet1 |
|  | Filter | <none> |
|  | Weight | <none> |
|  | Split File | <none> |
|  | N of Rows in Working Data File | 985 |
| Missing Value Handling | Definition of Missing | User-defined missing values are treated as missing. |
|  | Cases Used | Statistics for each test are based on all cases with valid data for the variable(s) used in that test. |
| Syntax | | NPAR TESTS  /M-W= TotalZarit BY Healthinsurance(1 2)  /MISSING ANALYSIS. |
| Resources | Processor Time | 00:00:00.00 |
|  | Elapsed Time | 00:00:00.00 |
|  | Number of Cases Allowed^a^ | 112347 |

| a. Based on availability of workspace memory. |
| --- |

[DataSet1] C:\Users\X415\Documents\Data analysis\Weaam's Data.sav

**Mann-Whitney Test**

| **Ranks** | | | | |
| --- | --- | --- | --- | --- |
|  | Health insurance | N | Mean Rank | Sum of Ranks |
| Total Zarit | Yes | 88 | 62.01 | 5457.00 |
|  | No | 35 | 61.97 | 2169.00 |
|  | Total | 123 |  |  |

| **Test Statistics^a^** | |
| --- | --- |
|  | Total Zarit |
| Mann-Whitney U | 1539.000 |
| Wilcoxon W | 2169.000 |
| Z | -.006 |
| Asymp. Sig. (2-tailed) | .996 |

| a. Grouping Variable: Health insurance |
| --- |

NPAR TESTS

/M-W= TotalZarit BY Physical(0 1)

/MISSING ANALYSIS.

**NPar Tests**

| **Notes** | | |
| --- | --- | --- |
| Output Created | | 05-DEC-2024 14:48:39 |
| Comments | |  |
| Input | Data | C:\Users\X415\Documents\Data analysis\Weaam's Data.sav |
|  | Active Dataset | DataSet1 |
|  | Filter | <none> |
|  | Weight | <none> |
|  | Split File | <none> |
|  | N of Rows in Working Data File | 985 |
| Missing Value Handling | Definition of Missing | User-defined missing values are treated as missing. |
|  | Cases Used | Statistics for each test are based on all cases with valid data for the variable(s) used in that test. |
| Syntax | | NPAR TESTS  /M-W= TotalZarit BY Physical(0 1)  /MISSING ANALYSIS. |
| Resources | Processor Time | 00:00:00.00 |
|  | Elapsed Time | 00:00:00.00 |
|  | Number of Cases Allowed^a^ | 112347 |

| a. Based on availability of workspace memory. |
| --- |

[DataSet1] C:\Users\X415\Documents\Data analysis\Weaam's Data.sav

**Mann-Whitney Test**

| **Ranks** | | | | |
| --- | --- | --- | --- | --- |
|  | Physical | N | Mean Rank | Sum of Ranks |
| Total Zarit | No | 95 | 58.89 | 5594.50 |
|  | Yes | 28 | 72.55 | 2031.50 |
|  | Total | 123 |  |  |

| **Test Statistics^a^** | |
| --- | --- |
|  | Total Zarit |
| Mann-Whitney U | 1034.500 |
| Wilcoxon W | 5594.500 |
| Z | -1.788 |
| Asymp. Sig. (2-tailed) | .074 |

| a. Grouping Variable: Physical |
| --- |

NPAR TESTS

/K-W=TotalZarit BY Relationshiptochild(1 5)

/MISSING ANALYSIS.

**NPar Tests**

| **Notes** | | |
| --- | --- | --- |
| Output Created | | 05-DEC-2024 14:50:59 |
| Comments | |  |
| Input | Data | C:\Users\X415\Documents\Data analysis\Weaam's Data.sav |
|  | Active Dataset | DataSet1 |
|  | Filter | <none> |
|  | Weight | <none> |
|  | Split File | <none> |
|  | N of Rows in Working Data File | 985 |
| Missing Value Handling | Definition of Missing | User-defined missing values are treated as missing. |
|  | Cases Used | Statistics for each test are based on all cases with valid data for the variable(s) used in that test. |
| Syntax | | NPAR TESTS  /K-W=TotalZarit BY Relationshiptochild(1 5)  /MISSING ANALYSIS. |
| Resources | Processor Time | 00:00:00.00 |
|  | Elapsed Time | 00:00:00.00 |
|  | Number of Cases Allowed^a^ | 112347 |

| a. Based on availability of workspace memory. |
| --- |

[DataSet1] C:\Users\X415\Documents\Data analysis\Weaam's Data.sav

**Kruskal-Wallis Test**

| **Ranks** | | | |
| --- | --- | --- | --- |
|  | Relationship to child | N | Mean Rank |
| Total Zarit | Mother | 104 | 61.71 |
|  | Father | 13 | 63.62 |
|  | Sibling | 1 | 80.50 |
|  | Aunt | 3 | 70.83 |
|  | Grandparent | 2 | 44.00 |
|  | Total | 123 |  |

| **Test Statistics^a,b^** | |
| --- | --- |
|  | Total Zarit |
| Chi-Square | 1.003 |
| df | 4 |
| Asymp. Sig. | .909 |

| a. Kruskal Wallis Test |
| --- |
| b. Grouping Variable: Relationship to child |

NPAR TESTS

/K-W=TotalZarit BY Age(1 4)

/MISSING ANALYSIS.

**NPar Tests**

| **Notes** | | |
| --- | --- | --- |
| Output Created | | 05-DEC-2024 14:52:48 |
| Comments | |  |
| Input | Data | C:\Users\X415\Documents\Data analysis\Weaam's Data.sav |
|  | Active Dataset | DataSet1 |
|  | Filter | <none> |
|  | Weight | <none> |
|  | Split File | <none> |
|  | N of Rows in Working Data File | 985 |
| Missing Value Handling | Definition of Missing | User-defined missing values are treated as missing. |
|  | Cases Used | Statistics for each test are based on all cases with valid data for the variable(s) used in that test. |
| Syntax | | NPAR TESTS  /K-W=TotalZarit BY Age(1 4)  /MISSING ANALYSIS. |
| Resources | Processor Time | 00:00:00.00 |
|  | Elapsed Time | 00:00:00.00 |
|  | Number of Cases Allowed^a^ | 112347 |

| a. Based on availability of workspace memory. |
| --- |

[DataSet1] C:\Users\X415\Documents\Data analysis\Weaam's Data.sav

**Kruskal-Wallis Test**

| **Ranks** | | | |
| --- | --- | --- | --- |
|  | Age | N | Mean Rank |
| Total Zarit | <20 Years | 2 | 101.75 |
|  | 20-30 Years | 51 | 58.54 |
|  | 31-40 Years | 44 | 57.42 |
|  | >40 Years | 26 | 73.48 |
|  | Total | 123 |  |

| **Test Statistics^a,b^** | |
| --- | --- |
|  | Total Zarit |
| Chi-Square | 6.431 |
| df | 3 |
| Asymp. Sig. | .092 |

| a. Kruskal Wallis Test |
| --- |
| b. Grouping Variable: Age |

NPAR TESTS

/K-W=TotalZarit BY Maritalstatus(1 4)

/MISSING ANALYSIS.

**NPar Tests**

| **Notes** | | |
| --- | --- | --- |
| Output Created | | 05-DEC-2024 14:53:28 |
| Comments | |  |
| Input | Data | C:\Users\X415\Documents\Data analysis\Weaam's Data.sav |
|  | Active Dataset | DataSet1 |
|  | Filter | <none> |
|  | Weight | <none> |
|  | Split File | <none> |
|  | N of Rows in Working Data File | 985 |
| Missing Value Handling | Definition of Missing | User-defined missing values are treated as missing. |
|  | Cases Used | Statistics for each test are based on all cases with valid data for the variable(s) used in that test. |
| Syntax | | NPAR TESTS  /K-W=TotalZarit BY Maritalstatus(1 4)  /MISSING ANALYSIS. |
| Resources | Processor Time | 00:00:00.00 |
|  | Elapsed Time | 00:00:00.00 |
|  | Number of Cases Allowed^a^ | 112347 |

| a. Based on availability of workspace memory. |
| --- |

[DataSet1] C:\Users\X415\Documents\Data analysis\Weaam's Data.sav

**Kruskal-Wallis Test**

| **Ranks** | | | |
| --- | --- | --- | --- |
|  | Marital status | N | Mean Rank |
| Total Zarit | Single | 2 | 57.50 |
|  | Married | 112 | 61.41 |
|  | Divorced | 3 | 70.83 |
|  | Widowed | 6 | 70.08 |
|  | Total | 123 |  |

| **Test Statistics^a,b^** | |
| --- | --- |
|  | Total Zarit |
| Chi-Square | .559 |
| df | 3 |
| Asymp. Sig. | .906 |

| a. Kruskal Wallis Test |
| --- |
| b. Grouping Variable: Marital status |

NPAR TESTS

/K-W=TotalZarit BY M.Education(1 6)

/MISSING ANALYSIS.

**NPar Tests**

| **Notes** | | |
| --- | --- | --- |
| Output Created | | 05-DEC-2024 14:54:08 |
| Comments | |  |
| Input | Data | C:\Users\X415\Documents\Data analysis\Weaam's Data.sav |
|  | Active Dataset | DataSet1 |
|  | Filter | <none> |
|  | Weight | <none> |
|  | Split File | <none> |
|  | N of Rows in Working Data File | 985 |
| Missing Value Handling | Definition of Missing | User-defined missing values are treated as missing. |
|  | Cases Used | Statistics for each test are based on all cases with valid data for the variable(s) used in that test. |
| Syntax | | NPAR TESTS  /K-W=TotalZarit BY M.Education(1 6)  /MISSING ANALYSIS. |
| Resources | Processor Time | 00:00:00.00 |
|  | Elapsed Time | 00:00:00.00 |
|  | Number of Cases Allowed^a^ | 112347 |

| a. Based on availability of workspace memory. |
| --- |

[DataSet1] C:\Users\X415\Documents\Data analysis\Weaam's Data.sav

**Kruskal-Wallis Test**

| **Ranks** | | | |
| --- | --- | --- | --- |
|  | M. Education | N | Mean Rank |
| Total Zarit | Illiteracy | 9 | 87.17 |
|  | Khalwa | 12 | 85.58 |
|  | Primary | 46 | 61.28 |
|  | Secondary | 33 | 49.83 |
|  | University | 23 | 58.74 |
|  | Total | 123 |  |

| **Test Statistics^a,b^** | |
| --- | --- |
|  | Total Zarit |
| Chi-Square | 13.881 |
| df | 4 |
| Asymp. Sig. | .008 |

| a. Kruskal Wallis Test |
| --- |
| b. Grouping Variable: M. Education |

NPAR TESTS

/K-W=TotalZarit BY F.Education(1 6)

/MISSING ANALYSIS.

**NPar Tests**

| **Notes** | | |
| --- | --- | --- |
| Output Created | | 05-DEC-2024 14:54:52 |
| Comments | |  |
| Input | Data | C:\Users\X415\Documents\Data analysis\Weaam's Data.sav |
|  | Active Dataset | DataSet1 |
|  | Filter | <none> |
|  | Weight | <none> |
|  | Split File | <none> |
|  | N of Rows in Working Data File | 985 |
| Missing Value Handling | Definition of Missing | User-defined missing values are treated as missing. |
|  | Cases Used | Statistics for each test are based on all cases with valid data for the variable(s) used in that test. |
| Syntax | | NPAR TESTS  /K-W=TotalZarit BY F.Education(1 6)  /MISSING ANALYSIS. |
| Resources | Processor Time | 00:00:00.00 |
|  | Elapsed Time | 00:00:00.00 |
|  | Number of Cases Allowed^a^ | 112347 |

| a. Based on availability of workspace memory. |
| --- |

[DataSet1] C:\Users\X415\Documents\Data analysis\Weaam's Data.sav

**Kruskal-Wallis Test**

| **Ranks** | | | |
| --- | --- | --- | --- |
|  | F. Education | N | Mean Rank |
| Total Zarit | Illiteracy | 13 | 92.23 |
|  | Khalwa | 10 | 58.20 |
|  | Primary | 49 | 62.59 |
|  | Secondary | 34 | 53.87 |
|  | University | 15 | 56.50 |
|  | Postgraduate | 2 | 49.50 |
|  | Total | 123 |  |

| **Test Statistics^a,b^** | |
| --- | --- |
|  | Total Zarit |
| Chi-Square | 11.924 |
| df | 5 |
| Asymp. Sig. | .036 |

| a. Kruskal Wallis Test |
| --- |
| b. Grouping Variable: F. Education |

NPAR TESTS

/K-W=TotalZarit BY F.Occupation(1 3)

/MISSING ANALYSIS.

**NPar Tests**

| **Notes** | | |
| --- | --- | --- |
| Output Created | | 05-DEC-2024 14:56:36 |
| Comments | |  |
| Input | Data | C:\Users\X415\Documents\Data analysis\Weaam's Data.sav |
|  | Active Dataset | DataSet1 |
|  | Filter | <none> |
|  | Weight | <none> |
|  | Split File | <none> |
|  | N of Rows in Working Data File | 985 |
| Missing Value Handling | Definition of Missing | User-defined missing values are treated as missing. |
|  | Cases Used | Statistics for each test are based on all cases with valid data for the variable(s) used in that test. |
| Syntax | | NPAR TESTS  /K-W=TotalZarit BY F.Occupation(1 3)  /MISSING ANALYSIS. |
| Resources | Processor Time | 00:00:00.00 |
|  | Elapsed Time | 00:00:00.00 |
|  | Number of Cases Allowed^a^ | 112347 |

| a. Based on availability of workspace memory. |
| --- |

[DataSet1] C:\Users\X415\Documents\Data analysis\Weaam's Data.sav

**Kruskal-Wallis Test**

| **Ranks** | | | |
| --- | --- | --- | --- |
|  | F. Occupation | N | Mean Rank |
| Total Zarit | Employee | 23 | 47.07 |
|  | Freework | 95 | 64.36 |
|  | Unemployed | 5 | 85.90 |
|  | Total | 123 |  |

| **Test Statistics^a,b^** | |
| --- | --- |
|  | Total Zarit |
| Chi-Square | 6.743 |
| df | 2 |
| Asymp. Sig. | .034 |

| a. Kruskal Wallis Test |
| --- |
| b. Grouping Variable: F. Occupation |

NPAR TESTS

/K-W=TotalZarit BY M.Occupation(1 3)

/MISSING ANALYSIS.

**NPar Tests**

| **Notes** | | |
| --- | --- | --- |
| Output Created | | 05-DEC-2024 14:58:05 |
| Comments | |  |
| Input | Data | C:\Users\X415\Documents\Data analysis\Weaam's Data.sav |
|  | Active Dataset | DataSet1 |
|  | Filter | <none> |
|  | Weight | <none> |
|  | Split File | <none> |
|  | N of Rows in Working Data File | 985 |
| Missing Value Handling | Definition of Missing | User-defined missing values are treated as missing. |
|  | Cases Used | Statistics for each test are based on all cases with valid data for the variable(s) used in that test. |
| Syntax | | NPAR TESTS  /K-W=TotalZarit BY M.Occupation(1 3)  /MISSING ANALYSIS. |
| Resources | Processor Time | 00:00:00.00 |
|  | Elapsed Time | 00:00:00.00 |
|  | Number of Cases Allowed^a^ | 112347 |

| a. Based on availability of workspace memory. |
| --- |

[DataSet1] C:\Users\X415\Documents\Data analysis\Weaam's Data.sav

**Kruskal-Wallis Test**

| **Ranks** | | | |
| --- | --- | --- | --- |
|  | M. Occupation | N | Mean Rank |
| Total Zarit | Employee | 12 | 70.13 |
|  | Housewife | 104 | 59.91 |
|  | Worker | 7 | 79.14 |
|  | Total | 123 |  |

| **Test Statistics^a,b^** | |
| --- | --- |
|  | Total Zarit |
| Chi-Square | 2.617 |
| df | 2 |
| Asymp. Sig. | .270 |

| a. Kruskal Wallis Test |
| --- |
| b. Grouping Variable: M. Occupation |

NPAR TESTS

/K-W=TotalZarit BY F.Tribe(1 7)

/MISSING ANALYSIS.

**NPar Tests**

| **Notes** | | |
| --- | --- | --- |
| Output Created | | 05-DEC-2024 14:58:54 |
| Comments | |  |
| Input | Data | C:\Users\X415\Documents\Data analysis\Weaam's Data.sav |
|  | Active Dataset | DataSet1 |
|  | Filter | <none> |
|  | Weight | <none> |
|  | Split File | <none> |
|  | N of Rows in Working Data File | 985 |
| Missing Value Handling | Definition of Missing | User-defined missing values are treated as missing. |
|  | Cases Used | Statistics for each test are based on all cases with valid data for the variable(s) used in that test. |
| Syntax | | NPAR TESTS  /K-W=TotalZarit BY F.Tribe(1 7)  /MISSING ANALYSIS. |
| Resources | Processor Time | 00:00:00.00 |
|  | Elapsed Time | 00:00:00.00 |
|  | Number of Cases Allowed^a^ | 112347 |

| a. Based on availability of workspace memory. |
| --- |

[DataSet1] C:\Users\X415\Documents\Data analysis\Weaam's Data.sav

**Kruskal-Wallis Test**

| **Ranks** | | | |
| --- | --- | --- | --- |
|  | F. Tribe | N | Mean Rank |
| Total Zarit | Bagara | 26 | 59.38 |
|  | Bideriya | 18 | 66.00 |
|  | Gawam'a | 4 | 108.25 |
|  | Falata | 19 | 57.42 |
|  | Bargo | 6 | 42.58 |
|  | Dar Hamid | 7 | 52.07 |
|  | Others | 43 | 63.95 |
|  | Total | 123 |  |

| **Test Statistics^a,b^** | |
| --- | --- |
|  | Total Zarit |
| Chi-Square | 9.928 |
| df | 6 |
| Asymp. Sig. | .128 |

| a. Kruskal Wallis Test |
| --- |
| b. Grouping Variable: F. Tribe |

NPAR TESTS

/K-W=TotalZarit BY M.Tribe(1 7)

/MISSING ANALYSIS.

**NPar Tests**

| **Notes** | | |
| --- | --- | --- |
| Output Created | | 05-DEC-2024 14:59:38 |
| Comments | |  |
| Input | Data | C:\Users\X415\Documents\Data analysis\Weaam's Data.sav |
|  | Active Dataset | DataSet1 |
|  | Filter | <none> |
|  | Weight | <none> |
|  | Split File | <none> |
|  | N of Rows in Working Data File | 985 |
| Missing Value Handling | Definition of Missing | User-defined missing values are treated as missing. |
|  | Cases Used | Statistics for each test are based on all cases with valid data for the variable(s) used in that test. |
| Syntax | | NPAR TESTS  /K-W=TotalZarit BY M.Tribe(1 7)  /MISSING ANALYSIS. |
| Resources | Processor Time | 00:00:00.00 |
|  | Elapsed Time | 00:00:00.00 |
|  | Number of Cases Allowed^a^ | 112347 |

| a. Based on availability of workspace memory. |
| --- |

[DataSet1] C:\Users\X415\Documents\Data analysis\Weaam's Data.sav

**Kruskal-Wallis Test**

| **Ranks** | | | |
| --- | --- | --- | --- |
|  | M. Tribe | N | Mean Rank |
| Total Zarit | Bagara | 20 | 64.55 |
|  | Bideriya | 15 | 65.47 |
|  | Gawam'a | 6 | 67.33 |
|  | Falata | 22 | 54.91 |
|  | Bargo | 6 | 42.58 |
|  | Dar Hamid | 10 | 66.50 |
|  | Others | 44 | 64.10 |
|  | Total | 123 |  |

| **Test Statistics^a,b^** | |
| --- | --- |
|  | Total Zarit |
| Chi-Square | 3.363 |
| df | 6 |
| Asymp. Sig. | .762 |

| a. Kruskal Wallis Test |
| --- |
| b. Grouping Variable: M. Tribe |

NPAR TESTS

/K-W=TotalZarit BY Income(1 4)

/MISSING ANALYSIS.

**NPar Tests**

| **Notes** | | |
| --- | --- | --- |
| Output Created | | 05-DEC-2024 15:00:32 |
| Comments | |  |
| Input | Data | C:\Users\X415\Documents\Data analysis\Weaam's Data.sav |
|  | Active Dataset | DataSet1 |
|  | Filter | <none> |
|  | Weight | <none> |
|  | Split File | <none> |
|  | N of Rows in Working Data File | 985 |
| Missing Value Handling | Definition of Missing | User-defined missing values are treated as missing. |
|  | Cases Used | Statistics for each test are based on all cases with valid data for the variable(s) used in that test. |
| Syntax | | NPAR TESTS  /K-W=TotalZarit BY Income(1 4)  /MISSING ANALYSIS. |
| Resources | Processor Time | 00:00:00.00 |
|  | Elapsed Time | 00:00:00.00 |
|  | Number of Cases Allowed^a^ | 112347 |

| a. Based on availability of workspace memory. |
| --- |

[DataSet1] C:\Users\X415\Documents\Data analysis\Weaam's Data.sav

**Kruskal-Wallis Test**

| **Ranks** | | | |
| --- | --- | --- | --- |
|  | F. Income | N | Mean Rank |
| Total Zarit | <50,000 SDG | 53 | 66.07 |
|  | 50,000-100,000 SDG | 49 | 55.27 |
|  | 100,000-200,000 SDG | 12 | 64.46 |
|  | >200,000 SDG | 9 | 71.44 |
|  | Total | 123 |  |

| **Test Statistics^a,b^** | |
| --- | --- |
|  | Total Zarit |
| Chi-Square | 3.147 |
| df | 3 |
| Asymp. Sig. | .369 |

| a. Kruskal Wallis Test |
| --- |
| b. Grouping Variable: F. Income |

NPAR TESTS

/K-W=TotalZarit BY Totalchildren(1 4)

/MISSING ANALYSIS.

**NPar Tests**

| **Notes** | | |
| --- | --- | --- |
| Output Created | | 05-DEC-2024 15:01:27 |
| Comments | |  |
| Input | Data | C:\Users\X415\Documents\Data analysis\Weaam's Data.sav |
|  | Active Dataset | DataSet1 |
|  | Filter | <none> |
|  | Weight | <none> |
|  | Split File | <none> |
|  | N of Rows in Working Data File | 985 |
| Missing Value Handling | Definition of Missing | User-defined missing values are treated as missing. |
|  | Cases Used | Statistics for each test are based on all cases with valid data for the variable(s) used in that test. |
| Syntax | | NPAR TESTS  /K-W=TotalZarit BY Totalchildren(1 4)  /MISSING ANALYSIS. |
| Resources | Processor Time | 00:00:00.00 |
|  | Elapsed Time | 00:00:00.00 |
|  | Number of Cases Allowed^a^ | 112347 |

| a. Based on availability of workspace memory. |
| --- |

[DataSet1] C:\Users\X415\Documents\Data analysis\Weaam's Data.sav

**Kruskal-Wallis Test**

| **Ranks** | | | |
| --- | --- | --- | --- |
|  | Total children | N | Mean Rank |
| Total Zarit | 1 | 11 | 55.18 |
|  | 2 | 23 | 55.17 |
|  | 3 | 21 | 62.98 |
|  | >3 | 67 | 64.25 |
|  | Total | 122 |  |

| **Test Statistics^a,b^** | |
| --- | --- |
|  | Total Zarit |
| Chi-Square | 1.538 |
| df | 3 |
| Asymp. Sig. | .673 |

| a. Kruskal Wallis Test |
| --- |
| b. Grouping Variable: Total children |

NPAR TESTS

/K-W=TotalZarit BY Sicklerchildren(1 4)

/MISSING ANALYSIS.

**NPar Tests**

| **Notes** | | |
| --- | --- | --- |
| Output Created | | 05-DEC-2024 15:02:18 |
| Comments | |  |
| Input | Data | C:\Users\X415\Documents\Data analysis\Weaam's Data.sav |
|  | Active Dataset | DataSet1 |
|  | Filter | <none> |
|  | Weight | <none> |
|  | Split File | <none> |
|  | N of Rows in Working Data File | 985 |
| Missing Value Handling | Definition of Missing | User-defined missing values are treated as missing. |
|  | Cases Used | Statistics for each test are based on all cases with valid data for the variable(s) used in that test. |
| Syntax | | NPAR TESTS  /K-W=TotalZarit BY Sicklerchildren(1 4)  /MISSING ANALYSIS. |
| Resources | Processor Time | 00:00:00.00 |
|  | Elapsed Time | 00:00:00.00 |
|  | Number of Cases Allowed^a^ | 112347 |

| a. Based on availability of workspace memory. |
| --- |

[DataSet1] C:\Users\X415\Documents\Data analysis\Weaam's Data.sav

**Kruskal-Wallis Test**

| **Ranks** | | | |
| --- | --- | --- | --- |
|  | Sickler children | N | Mean Rank |
| Total Zarit | 1 | 101 | 59.75 |
|  | 2 | 20 | 72.88 |
|  | 3 | 1 | 53.50 |
|  | >3 | 1 | 80.50 |
|  | Total | 123 |  |

| **Test Statistics^a,b^** | |
| --- | --- |
|  | Total Zarit |
| Chi-Square | 2.607 |
| df | 3 |
| Asymp. Sig. | .456 |

| a. Kruskal Wallis Test |
| --- |
| b. Grouping Variable: Sickler children |

NPAR TESTS

/K-W=TotalZarit BY Genchild(1 2)

/MISSING ANALYSIS.

**NPar Tests**

| **Notes** | | |
| --- | --- | --- |
| Output Created | | 05-DEC-2024 15:04:52 |
| Comments | |  |
| Input | Data | C:\Users\X415\Documents\Data analysis\Weaam's Data.sav |
|  | Active Dataset | DataSet1 |
|  | Filter | <none> |
|  | Weight | <none> |
|  | Split File | <none> |
|  | N of Rows in Working Data File | 985 |
| Missing Value Handling | Definition of Missing | User-defined missing values are treated as missing. |
|  | Cases Used | Statistics for each test are based on all cases with valid data for the variable(s) used in that test. |
| Syntax | | NPAR TESTS  /K-W=TotalZarit BY Genchild(1 2)  /MISSING ANALYSIS. |
| Resources | Processor Time | 00:00:00.00 |
|  | Elapsed Time | 00:00:00.00 |
|  | Number of Cases Allowed^a^ | 112347 |

| a. Based on availability of workspace memory. |
| --- |

[DataSet1] C:\Users\X415\Documents\Data analysis\Weaam's Data.sav

**Kruskal-Wallis Test**

| **Ranks** | | | |
| --- | --- | --- | --- |
|  | Gen child | N | Mean Rank |
| Total Zarit | Male | 64 | 63.46 |
|  | Female | 59 | 60.42 |
|  | Total | 123 |  |

| **Test Statistics^a,b^** | |
| --- | --- |
|  | Total Zarit |
| Chi-Square | .226 |
| df | 1 |
| Asymp. Sig. | .635 |

| a. Kruskal Wallis Test |
| --- |
| b. Grouping Variable: Gen child |

NPAR TESTS

/K-W=TotalZarit BY Agechild(1 4)

/MISSING ANALYSIS.

**NPar Tests**

| **Notes** | | |
| --- | --- | --- |
| Output Created | | 05-DEC-2024 15:12:17 |
| Comments | |  |
| Input | Data | C:\Users\X415\Documents\Data analysis\Weaam's Data.sav |
|  | Active Dataset | DataSet1 |
|  | Filter | <none> |
|  | Weight | <none> |
|  | Split File | <none> |
|  | N of Rows in Working Data File | 985 |
| Missing Value Handling | Definition of Missing | User-defined missing values are treated as missing. |
|  | Cases Used | Statistics for each test are based on all cases with valid data for the variable(s) used in that test. |
| Syntax | | NPAR TESTS  /K-W=TotalZarit BY Agechild(1 4)  /MISSING ANALYSIS. |
| Resources | Processor Time | 00:00:00.00 |
|  | Elapsed Time | 00:00:00.01 |
|  | Number of Cases Allowed^a^ | 112347 |

| a. Based on availability of workspace memory. |
| --- |

[DataSet1] C:\Users\X415\Documents\Data analysis\Weaam's Data.sav

**Kruskal-Wallis Test**

| **Ranks** | | | |
| --- | --- | --- | --- |
|  | Age child | N | Mean Rank |
| Total Zarit | 0-4 | 46 | 52.03 |
|  | 5-9 | 40 | 77.23 |
|  | 10-13 | 29 | 59.93 |
|  | 14-18 | 8 | 50.69 |
|  | Total | 123 |  |

| **Test Statistics^a,b^** | |
| --- | --- |
|  | Total Zarit |
| Chi-Square | 11.871 |
| df | 3 |
| Asymp. Sig. | .008 |

| a. Kruskal Wallis Test |
| --- |
| b. Grouping Variable: Age child |

NPAR TESTS

/K-W=TotalZarit BY Diagnosisage(1 3)

/MISSING ANALYSIS.

**NPar Tests**

| **Notes** | | |
| --- | --- | --- |
| Output Created | | 05-DEC-2024 15:13:13 |
| Comments | |  |
| Input | Data | C:\Users\X415\Documents\Data analysis\Weaam's Data.sav |
|  | Active Dataset | DataSet1 |
|  | Filter | <none> |
|  | Weight | <none> |
|  | Split File | <none> |
|  | N of Rows in Working Data File | 985 |
| Missing Value Handling | Definition of Missing | User-defined missing values are treated as missing. |
|  | Cases Used | Statistics for each test are based on all cases with valid data for the variable(s) used in that test. |
| Syntax | | NPAR TESTS  /K-W=TotalZarit BY Diagnosisage(1 3)  /MISSING ANALYSIS. |
| Resources | Processor Time | 00:00:00.02 |
|  | Elapsed Time | 00:00:00.01 |
|  | Number of Cases Allowed^a^ | 112347 |

| a. Based on availability of workspace memory. |
| --- |

[DataSet1] C:\Users\X415\Documents\Data analysis\Weaam's Data.sav

**Kruskal-Wallis Test**

| **Ranks** | | | |
| --- | --- | --- | --- |
|  | Diagnosis age | N | Mean Rank |
| Total Zarit | <6 Months | 27 | 69.83 |
|  | 6-12 Months | 65 | 59.61 |
|  | >12 Months | 31 | 60.19 |
|  | Total | 123 |  |

| **Test Statistics^a,b^** | |
| --- | --- |
|  | Total Zarit |
| Chi-Square | 1.687 |
| df | 2 |
| Asymp. Sig. | .430 |

| a. Kruskal Wallis Test |
| --- |
| b. Grouping Variable: Diagnosis age |

NPAR TESTS

/K-W=TotalZarit BY Hydroxyurea(1 3)

/MISSING ANALYSIS.

**NPar Tests**

| **Notes** | | |
| --- | --- | --- |
| Output Created | | 05-DEC-2024 15:13:52 |
| Comments | |  |
| Input | Data | C:\Users\X415\Documents\Data analysis\Weaam's Data.sav |
|  | Active Dataset | DataSet1 |
|  | Filter | <none> |
|  | Weight | <none> |
|  | Split File | <none> |
|  | N of Rows in Working Data File | 985 |
| Missing Value Handling | Definition of Missing | User-defined missing values are treated as missing. |
|  | Cases Used | Statistics for each test are based on all cases with valid data for the variable(s) used in that test. |
| Syntax | | NPAR TESTS  /K-W=TotalZarit BY Hydroxyurea(1 3)  /MISSING ANALYSIS. |
| Resources | Processor Time | 00:00:00.02 |
|  | Elapsed Time | 00:00:00.01 |
|  | Number of Cases Allowed^a^ | 112347 |

| a. Based on availability of workspace memory. |
| --- |

[DataSet1] C:\Users\X415\Documents\Data analysis\Weaam's Data.sav

**Kruskal-Wallis Test**

| **Ranks** | | | |
| --- | --- | --- | --- |
|  | Hydroxyurea | N | Mean Rank |
| Total Zarit | Regularly | 106 | 60.43 |
|  | Irregularly | 5 | 79.70 |
|  | Never | 12 | 68.50 |
|  | Total | 123 |  |

| **Test Statistics^a,b^** | |
| --- | --- |
|  | Total Zarit |
| Chi-Square | 1.849 |
| df | 2 |
| Asymp. Sig. | .397 |

| a. Kruskal Wallis Test |
| --- |
| b. Grouping Variable: Hydroxyurea |

NPAR TESTS

/K-W=TotalZarit BY Folicacid(1 3)

/MISSING ANALYSIS.

**NPar Tests**

| **Notes** | | |
| --- | --- | --- |
| Output Created | | 05-DEC-2024 15:14:29 |
| Comments | |  |
| Input | Data | C:\Users\X415\Documents\Data analysis\Weaam's Data.sav |
|  | Active Dataset | DataSet1 |
|  | Filter | <none> |
|  | Weight | <none> |
|  | Split File | <none> |
|  | N of Rows in Working Data File | 985 |
| Missing Value Handling | Definition of Missing | User-defined missing values are treated as missing. |
|  | Cases Used | Statistics for each test are based on all cases with valid data for the variable(s) used in that test. |
| Syntax | | NPAR TESTS  /K-W=TotalZarit BY Folicacid(1 3)  /MISSING ANALYSIS. |
| Resources | Processor Time | 00:00:00.00 |
|  | Elapsed Time | 00:00:00.00 |
|  | Number of Cases Allowed^a^ | 112347 |

| a. Based on availability of workspace memory. |
| --- |

[DataSet1] C:\Users\X415\Documents\Data analysis\Weaam's Data.sav

**Kruskal-Wallis Test**

| **Ranks** | | | |
| --- | --- | --- | --- |
|  | Folic acid | N | Mean Rank |
| Total Zarit | Regularly | 120 | 60.94 |
|  | Irregularly | 1 | 122.00 |
|  | Never | 2 | 95.75 |
|  | Total | 123 |  |

| **Test Statistics^a,b^** | |
| --- | --- |
|  | Total Zarit |
| Chi-Square | 4.762 |
| df | 2 |
| Asymp. Sig. | .092 |

| a. Kruskal Wallis Test |
| --- |
| b. Grouping Variable: Folic acid |

NPAR TESTS

/K-W=TotalZarit BY School(1 3)

/MISSING ANALYSIS.

**NPar Tests**

| **Notes** | | |
| --- | --- | --- |
| Output Created | | 05-DEC-2024 15:15:07 |
| Comments | |  |
| Input | Data | C:\Users\X415\Documents\Data analysis\Weaam's Data.sav |
|  | Active Dataset | DataSet1 |
|  | Filter | <none> |
|  | Weight | <none> |
|  | Split File | <none> |
|  | N of Rows in Working Data File | 985 |
| Missing Value Handling | Definition of Missing | User-defined missing values are treated as missing. |
|  | Cases Used | Statistics for each test are based on all cases with valid data for the variable(s) used in that test. |
| Syntax | | NPAR TESTS  /K-W=TotalZarit BY School(1 3)  /MISSING ANALYSIS. |
| Resources | Processor Time | 00:00:00.00 |
|  | Elapsed Time | 00:00:00.00 |
|  | Number of Cases Allowed^a^ | 112347 |

| a. Based on availability of workspace memory. |
| --- |

[DataSet1] C:\Users\X415\Documents\Data analysis\Weaam's Data.sav

**Kruskal-Wallis Test**

| **Ranks** | | | |
| --- | --- | --- | --- |
|  | School | N | Mean Rank |
| Total Zarit | Regularly | 39 | 55.19 |
|  | Irregularly | 14 | 75.64 |
|  | Never | 70 | 63.06 |
|  | Total | 123 |  |

| **Test Statistics^a,b^** | |
| --- | --- |
|  | Total Zarit |
| Chi-Square | 3.558 |
| df | 2 |
| Asymp. Sig. | .169 |

| a. Kruskal Wallis Test |
| --- |
| b. Grouping Variable: School |

NPAR TESTS

/K-W=TotalZarit BY Ifnotothepreviousquestion(1 5)

/MISSING ANALYSIS.

**NPar Tests**

| **Notes** | | |
| --- | --- | --- |
| Output Created | | 05-DEC-2024 15:19:27 |
| Comments | |  |
| Input | Data | C:\Users\X415\Documents\Data analysis\Weaam's Data.sav |
|  | Active Dataset | DataSet1 |
|  | Filter | <none> |
|  | Weight | <none> |
|  | Split File | <none> |
|  | N of Rows in Working Data File | 985 |
| Missing Value Handling | Definition of Missing | User-defined missing values are treated as missing. |
|  | Cases Used | Statistics for each test are based on all cases with valid data for the variable(s) used in that test. |
| Syntax | | NPAR TESTS  /K-W=TotalZarit BY Ifnotothepreviousquestion(1 5)  /MISSING ANALYSIS. |
| Resources | Processor Time | 00:00:00.02 |
|  | Elapsed Time | 00:00:00.01 |
|  | Number of Cases Allowed^a^ | 112347 |

| a. Based on availability of workspace memory. |
| --- |

[DataSet1] C:\Users\X415\Documents\Data analysis\Weaam's Data.sav

**Kruskal-Wallis Test**

| **Ranks** | | | |
| --- | --- | --- | --- |
|  | If no to the previous question | N | Mean Rank |
| Total Zarit | Young age | 51 | 54.53 |
|  | Illness | 30 | 79.37 |
|  | Financial | 2 | 107.00 |
|  | Yes | 40 | 56.25 |
|  | Total | 123 |  |

| **Test Statistics^a,b^** | |
| --- | --- |
|  | Total Zarit |
| Chi-Square | 13.674 |
| df | 3 |
| Asymp. Sig. | .003 |

| a. Kruskal Wallis Test |
| --- |
| b. Grouping Variable: If no to the previous question |

NPAR TESTS

/M-W= TotalZarit BY Genchild(1 2)

/MISSING ANALYSIS.

**NPar Tests**

| **Notes** | | |
| --- | --- | --- |
| Output Created | | 05-DEC-2024 15:21:37 |
| Comments | |  |
| Input | Data | C:\Users\X415\Documents\Data analysis\Weaam's Data.sav |
|  | Active Dataset | DataSet1 |
|  | Filter | <none> |
|  | Weight | <none> |
|  | Split File | <none> |
|  | N of Rows in Working Data File | 985 |
| Missing Value Handling | Definition of Missing | User-defined missing values are treated as missing. |
|  | Cases Used | Statistics for each test are based on all cases with valid data for the variable(s) used in that test. |
| Syntax | | NPAR TESTS  /M-W= TotalZarit BY Genchild(1 2)  /MISSING ANALYSIS. |
| Resources | Processor Time | 00:00:00.00 |
|  | Elapsed Time | 00:00:00.01 |
|  | Number of Cases Allowed^a^ | 112347 |

| a. Based on availability of workspace memory. |
| --- |

[DataSet1] C:\Users\X415\Documents\Data analysis\Weaam's Data.sav

**Mann-Whitney Test**

| **Ranks** | | | | |
| --- | --- | --- | --- | --- |
|  | Gen child | N | Mean Rank | Sum of Ranks |
| Total Zarit | Male | 64 | 63.46 | 4061.50 |
|  | Female | 59 | 60.42 | 3564.50 |
|  | Total | 123 |  |  |

| **Test Statistics^a^** | |
| --- | --- |
|  | Total Zarit |
| Mann-Whitney U | 1794.500 |
| Wilcoxon W | 3564.500 |
| Z | -.475 |
| Asymp. Sig. (2-tailed) | .635 |

| a. Grouping Variable: Gen child |
| --- |

* Custom Tables.

CTABLES

/VLABELS VARIABLES=Relationshiptochild Genderofthecaregiver Area Residence Age Maritalstatus M.Education F.Education F.Occupation M.Occupation F.Tribe M.Tribe Income Totalchildren Sicklerchildren Genchild Agechild Diagnosisage Hydroxyurea Folicacid

Healthinsurance School Ifnotothepreviousquestion burdenoncaregiver Physical Social Financial Psychological TotalZarit

DISPLAY=LABEL

/TABLE Relationshiptochild + Genderofthecaregiver + Area + Residence + Age + Maritalstatus + M.Education + F.Education + F.Occupation + M.Occupation + F.Tribe + M.Tribe + Income + Totalchildren + Sicklerchildren + Genchild + Agechild + Diagnosisage +

Hydroxyurea + Folicacid + Healthinsurance + School + Ifnotothepreviousquestion + burdenoncaregiver + Physical + Social + Financial + Psychological BY TotalZarit [MEDIAN, PTILE 25, PTILE 75]

/CATEGORIES VARIABLES=Relationshiptochild Genderofthecaregiver Area Residence Age Maritalstatus M.Education F.Education F.Occupation M.Occupation F.Tribe M.Tribe Income Totalchildren Sicklerchildren Genchild Agechild Diagnosisage Hydroxyurea Folicacid

Healthinsurance School Ifnotothepreviousquestion burdenoncaregiver Physical Social Financial Psychological ORDER=A KEY=VALUE EMPTY=INCLUDE.

**Custom Tables**

| **Notes** | | |
| --- | --- | --- |
| Output Created | | 05-DEC-2024 15:31:02 |
| Comments | |  |
| Input | Data | C:\Users\X415\Documents\Data analysis\Weaam's Data.sav |
|  | Active Dataset | DataSet1 |
|  | Filter | <none> |
|  | Weight | <none> |
|  | Split File | <none> |
|  | N of Rows in Working Data File | 985 |

| **Notes** | |
| --- | --- |
| Syntax | CTABLES  /VLABELS VARIABLES=Relationshiptochild Genderofthecaregiver Area Residence Age Maritalstatus M.Education F.Education F.Occupation M.Occupation F.Tribe M.Tribe Income Totalchildren Sicklerchildren Genchild Agechild Diagnosisage Hydroxyurea Folicacid  Healthinsurance School Ifnotothepreviousquestion burdenoncaregiver Physical Social Financial Psychological TotalZarit  DISPLAY=LABEL  /TABLE Relationshiptochild + Genderofthecaregiver + Area + Residence + Age + Maritalstatus + M.Education + F.Education + F.Occupation + M.Occupation + F.Tribe + M.Tribe + Income + Totalchildren + Sicklerchildren + Genchild + Agechild + Diagnosisage +  Hydroxyurea + Folicacid + Healthinsurance + School + Ifnotothepreviousquestion + burdenoncaregiver + Physical + Social + Financial + Psychological BY TotalZarit [MEDIAN, PTILE 25, PTILE 75]  /CATEGORIES VARIABLES=Relationshiptochild Genderofthecaregiver Area Residence Age Maritalstatus M.Education F.Education F.Occupation M.Occupation F.Tribe M.Tribe Income Totalchildren Sicklerchildren Genchild Agechild Diagnosisage Hydroxyurea Folicacid  Healthinsurance School Ifnotothepreviousquestion burdenoncaregiver Physical Social Financial Psychological ORDER=A KEY=VALUE EMPTY=INCLUDE. |

| **Notes** | | |
| --- | --- | --- |
| Resources | Processor Time | 00:00:00.03 |
|  | Elapsed Time | 00:00:00.03 |

[DataSet1] C:\Users\X415\Documents\Data analysis\Weaam's Data.sav

| **Table 1** | | | | |
| --- | --- | --- | --- | --- |
|  | | Total Zarit | | |
|  |  | Median | Percentile 25 | Percentile 75 |
| Relationship to child | Mother | 12 | 8 | 20 |
|  | Father | 13 | 9 | 16 |
|  | Sibling | 16 | 16 | 16 |
|  | Aunt | 17 | 8 | 18 |
|  | Grandparent | 9 | 8 | 10 |
| Gender of the caregiver | Male | 13 | 9 | 16 |
|  | Female | 12 | 8 | 19 |
| Area | Elobeid | 10 | 8 | 17 |
|  | Outside Elobeid | 16 | 8 | 25 |
| Residence | Rural | 14 | 9 | 25 |
|  | Urban | 11 | 8 | 17 |
| Age | <20 Years | 28 | 16 | 40 |
|  | 20-30 Years | 10 | 8 | 18 |
|  | 31-40 Years | 10 | 8 | 17 |
|  | >40 Years | 15 | 10 | 20 |
| Marital status | Single | 12 | 8 | 16 |
|  | Married | 12 | 8 | 18 |
|  | Divorced | 17 | 4 | 32 |
|  | Widowed | 15 | 9 | 22 |
| M. Education | Illiteracy | 21 | 16 | 28 |
|  | Khalwa | 18 | 14 | 26 |
|  | Primary | 11 | 8 | 20 |
|  | Secondary | 10 | 6 | 14 |
|  | University | 11 | 8 | 18 |
|  | Postgraduate | . | . | . |
| F. Education | Illiteracy | 20 | 17 | 24 |
|  | Khalwa | 10 | 7 | 18 |
|  | Primary | 11 | 8 | 19 |
|  | Secondary | 10 | 8 | 15 |
|  | University | 10 | 6 | 18 |
|  | Postgraduate | 10 | 8 | 12 |
| F. Occupation | Employee | 8 | 8 | 12 |

| **Table 1** | | | | |
| --- | --- | --- | --- | --- |
|  | | Total Zarit | | |
|  |  | Median | Percentile 25 | Percentile 75 |
| F. Occupation | Freework | 12 | 8 | 20 |
|  | Unemployed | 20 | 18 | 21 |
| M. Occupation | Employee | 14 | 9 | 21 |
|  | Housewife | 11 | 8 | 18 |
|  | Worker | 20 | 10 | 26 |
| F. Tribe | Bagara | 12 | 8 | 18 |
|  | Bideriya | 13 | 10 | 20 |
|  | Gawam'a | 26 | 24 | 27 |
|  | Falata | 10 | 8 | 16 |
|  | Bargo | 8 | 4 | 15 |
|  | Dar Hamid | 10 | 4 | 16 |
|  | Others | 10 | 8 | 21 |
| M. Tribe | Bagara | 14 | 8 | 19 |
|  | Bideriya | 12 | 8 | 17 |
|  | Gawam'a | 17 | 4 | 26 |
|  | Falata | 9 | 8 | 16 |
|  | Bargo | 8 | 4 | 15 |
|  | Dar Hamid | 15 | 8 | 20 |
|  | Others | 12 | 8 | 20 |
| F. Income | <50,000 SDG | 13 | 8 | 20 |
|  | 50,000-100,000 SDG | 10 | 7 | 17 |
|  | 100,000-200,000 SDG | 14 | 8 | 18 |
|  | >200,000 SDG | 15 | 10 | 18 |
| Total children | 1 | 10 | 6 | 20 |
|  | 2 | 9 | 6 | 16 |
|  | 3 | 10 | 8 | 19 |
|  | >3 | 12 | 8 | 19 |
| Sickler children | 1 | 11 | 8 | 18 |
|  | 2 | 16 | 10 | 21 |
|  | 3 | 10 | 10 | 10 |
|  | >3 | 16 | 16 | 16 |
| Gen child | Male | 12 | 8 | 20 |

| **Table 1** | | | | |
| --- | --- | --- | --- | --- |
|  | | Total Zarit | | |
|  |  | Median | Percentile 25 | Percentile 75 |
| Gen child | Female | 10 | 8 | 17 |
| Age child | 0-4 | 9 | 6 | 16 |
|  | 5-9 | 16 | 10 | 25 |
|  | 10-13 | 10 | 8 | 18 |
|  | 14-18 | 9 | 8 | 15 |
| Diagnosis age | <6 Months | 14 | 8 | 25 |
|  | 6-12 Months | 11 | 8 | 17 |
|  | >12 Months | 10 | 6 | 20 |
| Hydroxyurea | Regularly | 12 | 8 | 18 |
|  | Irregularly | 16 | 11 | 32 |
|  | Never | 13 | 8 | 26 |
| Folic acid | Regularly | 11 | 8 | 18 |
|  | Irregularly | 38 | 38 | 38 |
|  | Never | 24 | 15 | 32 |
| Health insurance | Yes | 12 | 8 | 18 |
|  | No | 12 | 8 | 20 |
| School | Regularly | 10 | 8 | 16 |
|  | Irregularly | 15 | 12 | 21 |
|  | Never | 12 | 8 | 20 |
| If no to the previous question | Young age | 9 | 6 | 16 |
|  | Illness | 17 | 12 | 22 |
|  | Financial | 26 | 20 | 32 |
|  | Psychological | . | . | . |
|  | Yes | 10 | 8 | 17 |
| burden on care giver | Physical | 10 | 8 | 18 |
|  | Social | 10 | 8 | 16 |
|  | Financial | 12 | 8 | 17 |
|  | Psychological | 11 | 6 | 17 |
|  | None | 9 | 8 | 12 |
|  | All | 21 | 16 | 28 |
|  | Financial and Psychological | 10 | 7 | 11 |

| **Table 1** | | | | |
| --- | --- | --- | --- | --- |
|  | | Total Zarit | | |
|  |  | Median | Percentile 25 | Percentile 75 |
| burden on care giver | Financial and Physical | 18 | 15 | 20 |
| Physical | No | 10 | 8 | 17 |
|  | Yes | 17 | 8 | 22 |
| Social | No | 11 | 8 | 18 |
|  | Yes | 16 | 8 | 21 |
| Financial | No | 10 | 8 | 17 |
|  | Yes | 13 | 8 | 20 |
| Psychological | No | 11 | 8 | 17 |
|  | Yes | 13 | 8 | 20 |

NPAR TESTS

/M-W= TotalZarit BY Physical(0 1)

/MISSING ANALYSIS.

**NPar Tests**

| **Notes** | | |
| --- | --- | --- |
| Output Created | | 05-DEC-2024 15:32:38 |
| Comments | |  |
| Input | Data | C:\Users\X415\Documents\Data analysis\Weaam's Data.sav |
|  | Active Dataset | DataSet1 |
|  | Filter | <none> |
|  | Weight | <none> |
|  | Split File | <none> |
|  | N of Rows in Working Data File | 985 |
| Missing Value Handling | Definition of Missing | User-defined missing values are treated as missing. |
|  | Cases Used | Statistics for each test are based on all cases with valid data for the variable(s) used in that test. |
| Syntax | | NPAR TESTS  /M-W= TotalZarit BY Physical(0 1)  /MISSING ANALYSIS. |
| Resources | Processor Time | 00:00:00.02 |
|  | Elapsed Time | 00:00:00.00 |
|  | Number of Cases Allowed^a^ | 112347 |

| a. Based on availability of workspace memory. |
| --- |

[DataSet1] C:\Users\X415\Documents\Data analysis\Weaam's Data.sav

**Mann-Whitney Test**

| **Ranks** | | | | |
| --- | --- | --- | --- | --- |
|  | Physical | N | Mean Rank | Sum of Ranks |
| Total Zarit | No | 95 | 58.89 | 5594.50 |
|  | Yes | 28 | 72.55 | 2031.50 |
|  | Total | 123 |  |  |

| **Test Statistics^a^** | |
| --- | --- |
|  | Total Zarit |
| Mann-Whitney U | 1034.500 |
| Wilcoxon W | 5594.500 |
| Z | -1.788 |
| Asymp. Sig. (2-tailed) | .074 |

| a. Grouping Variable: Physical |
| --- |

NPAR TESTS

/M-W= TotalZarit BY Social(0 1)

/MISSING ANALYSIS.

**NPar Tests**

| **Notes** | | |
| --- | --- | --- |
| Output Created | | 05-DEC-2024 15:33:20 |
| Comments | |  |
| Input | Data | C:\Users\X415\Documents\Data analysis\Weaam's Data.sav |
|  | Active Dataset | DataSet1 |
|  | Filter | <none> |
|  | Weight | <none> |
|  | Split File | <none> |
|  | N of Rows in Working Data File | 985 |
| Missing Value Handling | Definition of Missing | User-defined missing values are treated as missing. |
|  | Cases Used | Statistics for each test are based on all cases with valid data for the variable(s) used in that test. |
| Syntax | | NPAR TESTS  /M-W= TotalZarit BY Social(0 1)  /MISSING ANALYSIS. |
| Resources | Processor Time | 00:00:00.00 |
|  | Elapsed Time | 00:00:00.00 |
|  | Number of Cases Allowed^a^ | 112347 |

| a. Based on availability of workspace memory. |
| --- |

[DataSet1] C:\Users\X415\Documents\Data analysis\Weaam's Data.sav

**Mann-Whitney Test**

| **Ranks** | | | | |
| --- | --- | --- | --- | --- |
|  | Social | N | Mean Rank | Sum of Ranks |
| Total Zarit | No | 98 | 60.20 | 5899.50 |
|  | Yes | 25 | 69.06 | 1726.50 |
|  | Total | 123 |  |  |

| **Test Statistics^a^** | |
| --- | --- |
|  | Total Zarit |
| Mann-Whitney U | 1048.500 |
| Wilcoxon W | 5899.500 |
| Z | -1.113 |
| Asymp. Sig. (2-tailed) | .266 |

| a. Grouping Variable: Social |
| --- |

NPAR TESTS

/M-W= TotalZarit BY Financial(0 1)

/MISSING ANALYSIS.

**NPar Tests**

| **Notes** | | |
| --- | --- | --- |
| Output Created | | 05-DEC-2024 15:33:57 |
| Comments | |  |
| Input | Data | C:\Users\X415\Documents\Data analysis\Weaam's Data.sav |
|  | Active Dataset | DataSet1 |
|  | Filter | <none> |
|  | Weight | <none> |
|  | Split File | <none> |
|  | N of Rows in Working Data File | 985 |
| Missing Value Handling | Definition of Missing | User-defined missing values are treated as missing. |
|  | Cases Used | Statistics for each test are based on all cases with valid data for the variable(s) used in that test. |
| Syntax | | NPAR TESTS  /M-W= TotalZarit BY Financial(0 1)  /MISSING ANALYSIS. |
| Resources | Processor Time | 00:00:00.00 |
|  | Elapsed Time | 00:00:00.01 |
|  | Number of Cases Allowed^a^ | 112347 |

| a. Based on availability of workspace memory. |
| --- |

[DataSet1] C:\Users\X415\Documents\Data analysis\Weaam's Data.sav

**Mann-Whitney Test**

| **Ranks** | | | | |
| --- | --- | --- | --- | --- |
|  | Financial | N | Mean Rank | Sum of Ranks |
| Total Zarit | No | 56 | 56.08 | 3140.50 |
|  | Yes | 67 | 66.95 | 4485.50 |
|  | Total | 123 |  |  |

| **Test Statistics^a^** | |
| --- | --- |
|  | Total Zarit |
| Mann-Whitney U | 1544.500 |
| Wilcoxon W | 3140.500 |
| Z | -1.689 |
| Asymp. Sig. (2-tailed) | .091 |

| a. Grouping Variable: Financial |
| --- |

NPAR TESTS

/M-W= TotalZarit BY Psychological(0 1)

/MISSING ANALYSIS.

**NPar Tests**

| **Notes** | | |
| --- | --- | --- |
| Output Created | | 05-DEC-2024 15:34:38 |
| Comments | |  |
| Input | Data | C:\Users\X415\Documents\Data analysis\Weaam's Data.sav |
|  | Active Dataset | DataSet1 |
|  | Filter | <none> |
|  | Weight | <none> |
|  | Split File | <none> |
|  | N of Rows in Working Data File | 985 |
| Missing Value Handling | Definition of Missing | User-defined missing values are treated as missing. |
|  | Cases Used | Statistics for each test are based on all cases with valid data for the variable(s) used in that test. |
| Syntax | | NPAR TESTS  /M-W= TotalZarit BY Psychological(0 1)  /MISSING ANALYSIS. |
| Resources | Processor Time | 00:00:00.00 |
|  | Elapsed Time | 00:00:00.00 |
|  | Number of Cases Allowed^a^ | 112347 |

| a. Based on availability of workspace memory. |
| --- |

[DataSet1] C:\Users\X415\Documents\Data analysis\Weaam's Data.sav

**Mann-Whitney Test**

| **Ranks** | | | | |
| --- | --- | --- | --- | --- |
|  | Psychological | N | Mean Rank | Sum of Ranks |
| Total Zarit | No | 85 | 60.65 | 5155.50 |
|  | Yes | 38 | 65.01 | 2470.50 |
|  | Total | 123 |  |  |

| **Test Statistics^a^** | |
| --- | --- |
|  | Total Zarit |
| Mann-Whitney U | 1500.500 |
| Wilcoxon W | 5155.500 |
| Z | -.629 |
| Asymp. Sig. (2-tailed) | .529 |

| a. Grouping Variable: Psychological |
| --- |

GET

FILE="C:\Users\X415\Documents\Data analysis\Weaam's Data.sav".

DATASET NAME DataSet1 WINDOW=FRONT.

USE ALL.

COMPUTE filter_$=(VAR00002 = 1).

VARIABLE LABELS filter_$ 'VAR00002 = 1 (FILTER)'.

VALUE LABELS filter_$ 0 'Not Selected' 1 'Selected'.

FORMATS filter_$ (f1.0).

FILTER BY filter_$.

EXECUTE.

FREQUENCIES VARIABLES=Relationshiptochild Genderofthecaregiver Area Residence Age Maritalstatus M.Education F.Education F.Occupation M.Occupation F.Tribe M.Tribe Income Totalchildren Sicklerchildren Genchild Agechild Diagnosisage Hydroxyurea Folicacid

Healthinsurance School Ifnotothepreviousquestion burdenoncaregiver Genchild2 Agechild2 Diagnosisage2 Hydroxyurea2 Folicacid2 Healthinsurance2 School2 Ifno2 Burden2 Genchild3 Agechild3 Diagnosisage3 Hydroxyurea3 Folicacid3 Healthinsurance3 school3 Ifno3

Burden3 Q1 Q2 Q3 Q4 Q5 Q6 Q7 Q8 Q9 Q10 Q11 Q12 Physical Social Financial Psychological

/ORDER=ANALYSIS.

**Frequencies**

| **Notes** | | |
| --- | --- | --- |
| Output Created | | 13-DEC-2024 19:18:38 |
| Comments | |  |
| Input | Data | C:\Users\X415\Documents\Data analysis\Weaam's Data.sav |
|  | Active Dataset | DataSet1 |
|  | Filter | VAR00002 = 1 (FILTER) |
|  | Weight | <none> |
|  | Split File | <none> |
|  | N of Rows in Working Data File | 123 |
| Missing Value Handling | Definition of Missing | User-defined missing values are treated as missing. |
|  | Cases Used | Statistics are based on all cases with valid data. |

| **Notes** | | |
| --- | --- | --- |
| Syntax | | FREQUENCIES VARIABLES=Relationshiptochild Genderofthecaregiver Area Residence Age Maritalstatus M.Education F.Education F.Occupation M.Occupation F.Tribe M.Tribe Income Totalchildren Sicklerchildren Genchild Agechild Diagnosisage Hydroxyurea Folicacid  Healthinsurance School Ifnotothepreviousquestion burdenoncaregiver Genchild2 Agechild2 Diagnosisage2 Hydroxyurea2 Folicacid2 Healthinsurance2 School2 Ifno2 Burden2 Genchild3 Agechild3 Diagnosisage3 Hydroxyurea3 Folicacid3 Healthinsurance3 school3 Ifno3  Burden3 Q1 Q2 Q3 Q4 Q5 Q6 Q7 Q8 Q9 Q10 Q11 Q12 Physical Social Financial Psychological  /ORDER=ANALYSIS. |
| Resources | Processor Time | 00:00:00.03 |
|  | Elapsed Time | 00:00:00.03 |

[DataSet1] C:\Users\X415\Documents\Data analysis\Weaam's Data.sav

| **Statistics** | | | | | | | |
| --- | --- | --- | --- | --- | --- | --- | --- |
|  | | Relationship to child | Gender of the caregiver | Area | Residence | Age | Marital status |
| N | Valid | 123 | 123 | 123 | 123 | 123 | 123 |
|  | Missing | 0 | 0 | 0 | 0 | 0 | 0 |

| **Statistics** | | | | | | | |
| --- | --- | --- | --- | --- | --- | --- | --- |
|  | | M. Education | F. Education | F. Occupation | M. Occupation | F. Tribe | M. Tribe |
| N | Valid | 123 | 123 | 123 | 123 | 123 | 123 |
|  | Missing | 0 | 0 | 0 | 0 | 0 | 0 |

| **Statistics** | | | | | | | |
| --- | --- | --- | --- | --- | --- | --- | --- |
|  | | F. Income | Total children | Sickler children | Gen child | Age child | Diagnosis age |
| N | Valid | 123 | 122 | 123 | 123 | 123 | 123 |
|  | Missing | 0 | 1 | 0 | 0 | 0 | 0 |

| **Statistics** | | | | | | | |
| --- | --- | --- | --- | --- | --- | --- | --- |
|  | | Hydroxyurea | Folic acid | Health insurance | School | If no to the previous question | burden on care giver |
| N | Valid | 123 | 123 | 123 | 123 | 123 | 123 |
|  | Missing | 0 | 0 | 0 | 0 | 0 | 0 |

| **Statistics** | | | | | | |
| --- | --- | --- | --- | --- | --- | --- |
|  | | Gen child 2 | Age child 2 | Diagnosis age 2 | Hydroxyurea 2 | Folic acid 2 |
| N | Valid | 17 | 17 | 17 | 17 | 17 |
|  | Missing | 106 | 106 | 106 | 106 | 106 |

| **Statistics** | | | | | | | |
| --- | --- | --- | --- | --- | --- | --- | --- |
|  | | Health insurance 2 | School 2 | If no 2 | Burden 2 | Gen child 3 | Age child 3 |
| N | Valid | 17 | 17 | 8 | 17 | 1 | 1 |
|  | Missing | 106 | 106 | 115 | 106 | 122 | 122 |

| **Statistics** | | | | | | | |
| --- | --- | --- | --- | --- | --- | --- | --- |
|  | | Diagnosis age 3 | Hydroxyurea 3 | Folic acid 3 | Health insurance 3 | school 3 | If no 3 |
| N | Valid | 1 | 1 | 1 | 1 | 1 | 1 |
|  | Missing | 122 | 122 | 122 | 122 | 122 | 122 |

| **Statistics** | | | | | | | | |
| --- | --- | --- | --- | --- | --- | --- | --- | --- |
|  | | Burden 3 | Q1 | Q2 | Q3 | Q4 | Q5 | Q6 |
| N | Valid | 1 | 123 | 123 | 123 | 123 | 123 | 123 |
|  | Missing | 122 | 0 | 0 | 0 | 0 | 0 | 0 |

| **Statistics** | | | | | | | | |
| --- | --- | --- | --- | --- | --- | --- | --- | --- |
|  | | Q7 | Q8 | Q9 | Q10 | Q11 | Q12 | Physical |
| N | Valid | 123 | 123 | 123 | 123 | 123 | 123 | 123 |
|  | Missing | 0 | 0 | 0 | 0 | 0 | 0 | 0 |

| **Statistics** | | | | |
| --- | --- | --- | --- | --- |
|  | | Social | Financial | Psychological |
| N | Valid | 123 | 123 | 123 |
|  | Missing | 0 | 0 | 0 |

**Frequency Table**

| **Relationship to child** | | | | | |
| --- | --- | --- | --- | --- | --- |
|  | | Frequency | Percent | Valid Percent | Cumulative Percent |
| Valid | Mother | 104 | 84.6 | 84.6 | 84.6 |
|  | Father | 13 | 10.6 | 10.6 | 95.1 |
|  | Sibling | 1 | .8 | .8 | 95.9 |
|  | Aunt | 3 | 2.4 | 2.4 | 98.4 |
|  | Grandparent | 2 | 1.6 | 1.6 | 100.0 |
|  | Total | 123 | 100.0 | 100.0 |  |

| **Gender of the caregiver** | | | | | |
| --- | --- | --- | --- | --- | --- |
|  | | Frequency | Percent | Valid Percent | Cumulative Percent |
| Valid | Male | 13 | 10.6 | 10.6 | 10.6 |
|  | Female | 110 | 89.4 | 89.4 | 100.0 |
|  | Total | 123 | 100.0 | 100.0 |  |

| **Area** | | | | | |
| --- | --- | --- | --- | --- | --- |
|  | | Frequency | Percent | Valid Percent | Cumulative Percent |
| Valid | Elobeid | 75 | 61.0 | 61.0 | 61.0 |
|  | Outside Elobeid | 48 | 39.0 | 39.0 | 100.0 |
|  | Total | 123 | 100.0 | 100.0 |  |

| **Residence** | | | | | |
| --- | --- | --- | --- | --- | --- |
|  | | Frequency | Percent | Valid Percent | Cumulative Percent |
| Valid | Rural | 34 | 27.6 | 27.6 | 27.6 |
|  | Urban | 89 | 72.4 | 72.4 | 100.0 |
|  | Total | 123 | 100.0 | 100.0 |  |

| **Age** | | | | | |
| --- | --- | --- | --- | --- | --- |
|  | | Frequency | Percent | Valid Percent | Cumulative Percent |
| Valid | <20 Years | 2 | 1.6 | 1.6 | 1.6 |
|  | 20-30 Years | 51 | 41.5 | 41.5 | 43.1 |
|  | 31-40 Years | 44 | 35.8 | 35.8 | 78.9 |
|  | >40 Years | 26 | 21.1 | 21.1 | 100.0 |
|  | Total | 123 | 100.0 | 100.0 |  |

| **Marital status** | | | | | |
| --- | --- | --- | --- | --- | --- |
|  | | Frequency | Percent | Valid Percent | Cumulative Percent |
| Valid | Single | 2 | 1.6 | 1.6 | 1.6 |
|  | Married | 112 | 91.1 | 91.1 | 92.7 |
|  | Divorced | 3 | 2.4 | 2.4 | 95.1 |
|  | Widowed | 6 | 4.9 | 4.9 | 100.0 |
|  | Total | 123 | 100.0 | 100.0 |  |

| **M. Education** | | | | | |
| --- | --- | --- | --- | --- | --- |
|  | | Frequency | Percent | Valid Percent | Cumulative Percent |
| Valid | Illiteracy | 9 | 7.3 | 7.3 | 7.3 |
|  | Khalwa | 12 | 9.8 | 9.8 | 17.1 |
|  | Primary | 46 | 37.4 | 37.4 | 54.5 |
|  | Secondary | 33 | 26.8 | 26.8 | 81.3 |
|  | University | 23 | 18.7 | 18.7 | 100.0 |
|  | Total | 123 | 100.0 | 100.0 |  |

| **F. Education** | | | | | |
| --- | --- | --- | --- | --- | --- |
|  | | Frequency | Percent | Valid Percent | Cumulative Percent |
| Valid | Illiteracy | 13 | 10.6 | 10.6 | 10.6 |
|  | Khalwa | 10 | 8.1 | 8.1 | 18.7 |
|  | Primary | 49 | 39.8 | 39.8 | 58.5 |
|  | Secondary | 34 | 27.6 | 27.6 | 86.2 |
|  | University | 15 | 12.2 | 12.2 | 98.4 |
|  | Postgraduate | 2 | 1.6 | 1.6 | 100.0 |
|  | Total | 123 | 100.0 | 100.0 |  |

| **F. Occupation** | | | | | |
| --- | --- | --- | --- | --- | --- |
|  | | Frequency | Percent | Valid Percent | Cumulative Percent |
| Valid | Employee | 23 | 18.7 | 18.7 | 18.7 |
|  | Freework | 95 | 77.2 | 77.2 | 95.9 |
|  | Unemployed | 5 | 4.1 | 4.1 | 100.0 |
|  | Total | 123 | 100.0 | 100.0 |  |

| **M. Occupation** | | | | | |
| --- | --- | --- | --- | --- | --- |
|  | | Frequency | Percent | Valid Percent | Cumulative Percent |
| Valid | Employee | 12 | 9.8 | 9.8 | 9.8 |
|  | Housewife | 104 | 84.6 | 84.6 | 94.3 |
|  | Worker | 7 | 5.7 | 5.7 | 100.0 |
|  | Total | 123 | 100.0 | 100.0 |  |

| **F. Tribe** | | | | | |
| --- | --- | --- | --- | --- | --- |
|  | | Frequency | Percent | Valid Percent | Cumulative Percent |
| Valid | Bagara | 26 | 21.1 | 21.1 | 21.1 |
|  | Bideriya | 18 | 14.6 | 14.6 | 35.8 |
|  | Gawam'a | 4 | 3.3 | 3.3 | 39.0 |
|  | Falata | 19 | 15.4 | 15.4 | 54.5 |
|  | Bargo | 6 | 4.9 | 4.9 | 59.3 |
|  | Dar Hamid | 7 | 5.7 | 5.7 | 65.0 |
|  | Others | 43 | 35.0 | 35.0 | 100.0 |
|  | Total | 123 | 100.0 | 100.0 |  |

| **M. Tribe** | | | | | |
| --- | --- | --- | --- | --- | --- |
|  | | Frequency | Percent | Valid Percent | Cumulative Percent |
| Valid | Bagara | 20 | 16.3 | 16.3 | 16.3 |
|  | Bideriya | 15 | 12.2 | 12.2 | 28.5 |
|  | Gawam'a | 6 | 4.9 | 4.9 | 33.3 |
|  | Falata | 22 | 17.9 | 17.9 | 51.2 |
|  | Bargo | 6 | 4.9 | 4.9 | 56.1 |
|  | Dar Hamid | 10 | 8.1 | 8.1 | 64.2 |
|  | Others | 44 | 35.8 | 35.8 | 100.0 |
|  | Total | 123 | 100.0 | 100.0 |  |

| **F. Income** | | | | | |
| --- | --- | --- | --- | --- | --- |
|  | | Frequency | Percent | Valid Percent | Cumulative Percent |
| Valid | <50,000 SDG | 53 | 43.1 | 43.1 | 43.1 |
|  | 50,000-100,000 SDG | 49 | 39.8 | 39.8 | 82.9 |
|  | 100,000-200,000 SDG | 12 | 9.8 | 9.8 | 92.7 |
|  | >200,000 SDG | 9 | 7.3 | 7.3 | 100.0 |
|  | Total | 123 | 100.0 | 100.0 |  |

| **Total children** | | | | | |
| --- | --- | --- | --- | --- | --- |
|  | | Frequency | Percent | Valid Percent | Cumulative Percent |
| Valid | 1 | 11 | 8.9 | 9.0 | 9.0 |
|  | 2 | 23 | 18.7 | 18.9 | 27.9 |
|  | 3 | 21 | 17.1 | 17.2 | 45.1 |
|  | >3 | 67 | 54.5 | 54.9 | 100.0 |
|  | Total | 122 | 99.2 | 100.0 |  |
| Missing | System | 1 | .8 |  |  |
| Total | | 123 | 100.0 |  |  |

| **Sickler children** | | | | | |
| --- | --- | --- | --- | --- | --- |
|  | | Frequency | Percent | Valid Percent | Cumulative Percent |
| Valid | 1 | 101 | 82.1 | 82.1 | 82.1 |
|  | 2 | 20 | 16.3 | 16.3 | 98.4 |
|  | 3 | 1 | .8 | .8 | 99.2 |
|  | >3 | 1 | .8 | .8 | 100.0 |
|  | Total | 123 | 100.0 | 100.0 |  |

| **Gen child** | | | | | |
| --- | --- | --- | --- | --- | --- |
|  | | Frequency | Percent | Valid Percent | Cumulative Percent |
| Valid | Male | 64 | 52.0 | 52.0 | 52.0 |
|  | Female | 59 | 48.0 | 48.0 | 100.0 |
|  | Total | 123 | 100.0 | 100.0 |  |

| **Age child** | | | | | |
| --- | --- | --- | --- | --- | --- |
|  | | Frequency | Percent | Valid Percent | Cumulative Percent |
| Valid | 0-4 | 46 | 37.4 | 37.4 | 37.4 |
|  | 5-9 | 40 | 32.5 | 32.5 | 69.9 |
|  | 10-13 | 29 | 23.6 | 23.6 | 93.5 |
|  | 14-18 | 8 | 6.5 | 6.5 | 100.0 |
|  | Total | 123 | 100.0 | 100.0 |  |

| **Diagnosis age** | | | | | |
| --- | --- | --- | --- | --- | --- |
|  | | Frequency | Percent | Valid Percent | Cumulative Percent |
| Valid | <6 Months | 27 | 22.0 | 22.0 | 22.0 |
|  | 6-12 Months | 65 | 52.8 | 52.8 | 74.8 |
|  | >12 Months | 31 | 25.2 | 25.2 | 100.0 |
|  | Total | 123 | 100.0 | 100.0 |  |

| **Hydroxyurea** | | | | | |
| --- | --- | --- | --- | --- | --- |
|  | | Frequency | Percent | Valid Percent | Cumulative Percent |
| Valid | Regularly | 106 | 86.2 | 86.2 | 86.2 |
|  | Irregularly | 5 | 4.1 | 4.1 | 90.2 |
|  | Never | 12 | 9.8 | 9.8 | 100.0 |
|  | Total | 123 | 100.0 | 100.0 |  |

| **Folic acid** | | | | | |
| --- | --- | --- | --- | --- | --- |
|  | | Frequency | Percent | Valid Percent | Cumulative Percent |
| Valid | Regularly | 120 | 97.6 | 97.6 | 97.6 |
|  | Irregularly | 1 | .8 | .8 | 98.4 |
|  | Never | 2 | 1.6 | 1.6 | 100.0 |
|  | Total | 123 | 100.0 | 100.0 |  |

| **Health insurance** | | | | | |
| --- | --- | --- | --- | --- | --- |
|  | | Frequency | Percent | Valid Percent | Cumulative Percent |
| Valid | Yes | 88 | 71.5 | 71.5 | 71.5 |
|  | No | 35 | 28.5 | 28.5 | 100.0 |
|  | Total | 123 | 100.0 | 100.0 |  |

| **School** | | | | | |
| --- | --- | --- | --- | --- | --- |
|  | | Frequency | Percent | Valid Percent | Cumulative Percent |
| Valid | Regularly | 39 | 31.7 | 31.7 | 31.7 |
|  | Irregularly | 14 | 11.4 | 11.4 | 43.1 |
|  | Never | 70 | 56.9 | 56.9 | 100.0 |
|  | Total | 123 | 100.0 | 100.0 |  |

| **If no to the previous question** | | | | | |
| --- | --- | --- | --- | --- | --- |
|  | | Frequency | Percent | Valid Percent | Cumulative Percent |
| Valid | Young age | 51 | 41.5 | 41.5 | 41.5 |
|  | Illness | 30 | 24.4 | 24.4 | 65.9 |
|  | Financial | 2 | 1.6 | 1.6 | 67.5 |
|  | Yes | 40 | 32.5 | 32.5 | 100.0 |
|  | Total | 123 | 100.0 | 100.0 |  |

| **burden on care giver** | | | | | |
| --- | --- | --- | --- | --- | --- |
|  | | Frequency | Percent | Valid Percent | Cumulative Percent |
| Valid | Physical | 15 | 12.2 | 12.2 | 12.2 |
|  | Social | 14 | 11.4 | 11.4 | 23.6 |
|  | Financial | 45 | 36.6 | 36.6 | 60.2 |
|  | Psychological | 18 | 14.6 | 14.6 | 74.8 |
|  | None | 9 | 7.3 | 7.3 | 82.1 |
|  | All | 11 | 8.9 | 8.9 | 91.1 |
|  | Financial and Psychological | 9 | 7.3 | 7.3 | 98.4 |
|  | Financial and Physical | 2 | 1.6 | 1.6 | 100.0 |
|  | Total | 123 | 100.0 | 100.0 |  |

| **Gen child 2** | | | | | |
| --- | --- | --- | --- | --- | --- |
|  | | Frequency | Percent | Valid Percent | Cumulative Percent |
| Valid | Male | 6 | 4.9 | 35.3 | 35.3 |
|  | Female | 11 | 8.9 | 64.7 | 100.0 |
|  | Total | 17 | 13.8 | 100.0 |  |
| Missing | 99 | 106 | 86.2 |  |  |
| Total | | 123 | 100.0 |  |  |

| **Age child 2** | | | | | |
| --- | --- | --- | --- | --- | --- |
|  | | Frequency | Percent | Valid Percent | Cumulative Percent |
| Valid | 0-4 | 3 | 2.4 | 17.6 | 17.6 |
|  | 5-9 | 6 | 4.9 | 35.3 | 52.9 |
|  | 10-13 | 3 | 2.4 | 17.6 | 70.6 |
|  | 14-18 | 5 | 4.1 | 29.4 | 100.0 |
|  | Total | 17 | 13.8 | 100.0 |  |
| Missing | 99 | 106 | 86.2 |  |  |
| Total | | 123 | 100.0 |  |  |

| **Diagnosis age 2** | | | | | |
| --- | --- | --- | --- | --- | --- |
|  | | Frequency | Percent | Valid Percent | Cumulative Percent |
| Valid | <6 Months | 6 | 4.9 | 35.3 | 35.3 |
|  | 6-12 Months | 7 | 5.7 | 41.2 | 76.5 |
|  | >12 Months | 4 | 3.3 | 23.5 | 100.0 |
|  | Total | 17 | 13.8 | 100.0 |  |
| Missing | 99 | 106 | 86.2 |  |  |
| Total | | 123 | 100.0 |  |  |

| **Hydroxyurea 2** | | | | | |
| --- | --- | --- | --- | --- | --- |
|  | | Frequency | Percent | Valid Percent | Cumulative Percent |
| Valid | Regularly | 15 | 12.2 | 88.2 | 88.2 |
|  | Never | 2 | 1.6 | 11.8 | 100.0 |
|  | Total | 17 | 13.8 | 100.0 |  |
| Missing | 99 | 106 | 86.2 |  |  |
| Total | | 123 | 100.0 |  |  |

| **Folic acid 2** | | | | | |
| --- | --- | --- | --- | --- | --- |
|  | | Frequency | Percent | Valid Percent | Cumulative Percent |
| Valid | Regularly | 17 | 13.8 | 100.0 | 100.0 |
| Missing | 99 | 106 | 86.2 |  |  |
| Total | | 123 | 100.0 |  |  |

| **Health insurance 2** | | | | | |
| --- | --- | --- | --- | --- | --- |
|  | | Frequency | Percent | Valid Percent | Cumulative Percent |
| Valid | Yes | 13 | 10.6 | 76.5 | 76.5 |
|  | No | 4 | 3.3 | 23.5 | 100.0 |
|  | Total | 17 | 13.8 | 100.0 |  |
| Missing | 99 | 106 | 86.2 |  |  |
| Total | | 123 | 100.0 |  |  |

| **School 2** | | | | | |
| --- | --- | --- | --- | --- | --- |
|  | | Frequency | Percent | Valid Percent | Cumulative Percent |
| Valid | Regularly | 9 | 7.3 | 52.9 | 52.9 |
|  | Irregularly | 3 | 2.4 | 17.6 | 70.6 |
|  | Never | 5 | 4.1 | 29.4 | 100.0 |
|  | Total | 17 | 13.8 | 100.0 |  |
| Missing | 99 | 106 | 86.2 |  |  |
| Total | | 123 | 100.0 |  |  |

| **If no 2** | | | | | |
| --- | --- | --- | --- | --- | --- |
|  | | Frequency | Percent | Valid Percent | Cumulative Percent |
| Valid | Young age | 3 | 2.4 | 37.5 | 37.5 |
|  | Illness | 4 | 3.3 | 50.0 | 87.5 |
|  | Yes | 1 | .8 | 12.5 | 100.0 |
|  | Total | 8 | 6.5 | 100.0 |  |
| Missing | 99 | 115 | 93.5 |  |  |
| Total | | 123 | 100.0 |  |  |

| **Burden 2** | | | | | |
| --- | --- | --- | --- | --- | --- |
|  | | Frequency | Percent | Valid Percent | Cumulative Percent |
| Valid | Physical | 4 | 3.3 | 23.5 | 23.5 |
|  | Social | 4 | 3.3 | 23.5 | 47.1 |
|  | Financial | 6 | 4.9 | 35.3 | 82.4 |
|  | Psychological | 2 | 1.6 | 11.8 | 94.1 |
|  | Financial and Physical | 1 | .8 | 5.9 | 100.0 |
|  | Total | 17 | 13.8 | 100.0 |  |
| Missing | 99 | 106 | 86.2 |  |  |
| Total | | 123 | 100.0 |  |  |

| **Gen child 3** | | | | | |
| --- | --- | --- | --- | --- | --- |
|  | | Frequency | Percent | Valid Percent | Cumulative Percent |
| Valid | Female | 1 | .8 | 100.0 | 100.0 |
| Missing | 99 | 122 | 99.2 |  |  |
| Total | | 123 | 100.0 |  |  |

| **Age child 3** | | | | | |
| --- | --- | --- | --- | --- | --- |
|  | | Frequency | Percent | Valid Percent | Cumulative Percent |
| Valid | 0-4 | 1 | .8 | 100.0 | 100.0 |
| Missing | 99 | 122 | 99.2 |  |  |
| Total | | 123 | 100.0 |  |  |

| **Diagnosis age 3** | | | | | |
| --- | --- | --- | --- | --- | --- |
|  | | Frequency | Percent | Valid Percent | Cumulative Percent |
| Valid | <6 Months | 1 | .8 | 100.0 | 100.0 |
| Missing | 99 | 122 | 99.2 |  |  |
| Total | | 123 | 100.0 |  |  |

| **Hydroxyurea 3** | | | | | |
| --- | --- | --- | --- | --- | --- |
|  | | Frequency | Percent | Valid Percent | Cumulative Percent |
| Valid | Regularly | 1 | .8 | 100.0 | 100.0 |
| Missing | 99 | 122 | 99.2 |  |  |
| Total | | 123 | 100.0 |  |  |

| **Folic acid 3** | | | | | |
| --- | --- | --- | --- | --- | --- |
|  | | Frequency | Percent | Valid Percent | Cumulative Percent |
| Valid | Regularly | 1 | .8 | 100.0 | 100.0 |
| Missing | 99 | 122 | 99.2 |  |  |
| Total | | 123 | 100.0 |  |  |

| **Health insurance 3** | | | | | |
| --- | --- | --- | --- | --- | --- |
|  | | Frequency | Percent | Valid Percent | Cumulative Percent |
| Valid | No | 1 | .8 | 100.0 | 100.0 |
| Missing | 99 | 122 | 99.2 |  |  |
| Total | | 123 | 100.0 |  |  |

| **school 3** | | | | | |
| --- | --- | --- | --- | --- | --- |
|  | | Frequency | Percent | Valid Percent | Cumulative Percent |
| Valid | Never | 1 | .8 | 100.0 | 100.0 |
| Missing | 99 | 122 | 99.2 |  |  |
| Total | | 123 | 100.0 |  |  |

| **If no 3** | | | | | |
| --- | --- | --- | --- | --- | --- |
|  | | Frequency | Percent | Valid Percent | Cumulative Percent |
| Valid | Young age | 1 | .8 | 100.0 | 100.0 |
| Missing | 99 | 122 | 99.2 |  |  |
| Total | | 123 | 100.0 |  |  |

| **Burden 3** | | | | | |
| --- | --- | --- | --- | --- | --- |
|  | | Frequency | Percent | Valid Percent | Cumulative Percent |
| Valid | Financial | 1 | .8 | 100.0 | 100.0 |
| Missing | 99 | 122 | 99.2 |  |  |
| Total | | 123 | 100.0 |  |  |

| **Q1** | | | | | |
| --- | --- | --- | --- | --- | --- |
|  | | Frequency | Percent | Valid Percent | Cumulative Percent |
| Valid | Never | 66 | 53.7 | 53.7 | 53.7 |
|  | Rarely | 5 | 4.1 | 4.1 | 57.7 |
|  | Sometimes | 25 | 20.3 | 20.3 | 78.0 |
|  | Frequently | 4 | 3.3 | 3.3 | 81.3 |
|  | Nearly always | 23 | 18.7 | 18.7 | 100.0 |
|  | Total | 123 | 100.0 | 100.0 |  |

| **Q2** | | | | | |
| --- | --- | --- | --- | --- | --- |
|  | | Frequency | Percent | Valid Percent | Cumulative Percent |
| Valid | Never | 61 | 49.6 | 49.6 | 49.6 |
|  | Rarely | 13 | 10.6 | 10.6 | 60.2 |
|  | Sometimes | 20 | 16.3 | 16.3 | 76.4 |
|  | Frequently | 10 | 8.1 | 8.1 | 84.6 |
|  | Nearly always | 19 | 15.4 | 15.4 | 100.0 |
|  | Total | 123 | 100.0 | 100.0 |  |

| **Q3** | | | | | |
| --- | --- | --- | --- | --- | --- |
|  | | Frequency | Percent | Valid Percent | Cumulative Percent |
| Valid | Never | 88 | 71.5 | 71.5 | 71.5 |
|  | Rarely | 11 | 8.9 | 8.9 | 80.5 |
|  | Sometimes | 18 | 14.6 | 14.6 | 95.1 |
|  | Frequently | 3 | 2.4 | 2.4 | 97.6 |
|  | Nearly always | 3 | 2.4 | 2.4 | 100.0 |
|  | Total | 123 | 100.0 | 100.0 |  |

| **Q4** | | | | | |
| --- | --- | --- | --- | --- | --- |
|  | | Frequency | Percent | Valid Percent | Cumulative Percent |
| Valid | Never | 96 | 78.0 | 78.0 | 78.0 |
|  | Rarely | 3 | 2.4 | 2.4 | 80.5 |
|  | Sometimes | 11 | 8.9 | 8.9 | 89.4 |
|  | Frequently | 5 | 4.1 | 4.1 | 93.5 |
|  | Nearly always | 8 | 6.5 | 6.5 | 100.0 |
|  | Total | 123 | 100.0 | 100.0 |  |

| **Q5** | | | | | |
| --- | --- | --- | --- | --- | --- |
|  | | Frequency | Percent | Valid Percent | Cumulative Percent |
| Valid | Never | 78 | 63.4 | 63.4 | 63.4 |
|  | Rarely | 10 | 8.1 | 8.1 | 71.5 |
|  | Sometimes | 26 | 21.1 | 21.1 | 92.7 |
|  | Frequently | 1 | .8 | .8 | 93.5 |
|  | Nearly always | 8 | 6.5 | 6.5 | 100.0 |
|  | Total | 123 | 100.0 | 100.0 |  |

| **Q6** | | | | | |
| --- | --- | --- | --- | --- | --- |
|  | | Frequency | Percent | Valid Percent | Cumulative Percent |
| Valid | Never | 76 | 61.8 | 61.8 | 61.8 |
|  | Rarely | 12 | 9.8 | 9.8 | 71.5 |
|  | Sometimes | 12 | 9.8 | 9.8 | 81.3 |
|  | Frequently | 6 | 4.9 | 4.9 | 86.2 |
|  | Nearly always | 17 | 13.8 | 13.8 | 100.0 |
|  | Total | 123 | 100.0 | 100.0 |  |

| **Q7** | | | | | |
| --- | --- | --- | --- | --- | --- |
|  | | Frequency | Percent | Valid Percent | Cumulative Percent |
| Valid | Never | 84 | 68.3 | 68.3 | 68.3 |
|  | Rarely | 8 | 6.5 | 6.5 | 74.8 |
|  | Sometimes | 16 | 13.0 | 13.0 | 87.8 |
|  | Frequently | 1 | .8 | .8 | 88.6 |
|  | Nearly always | 14 | 11.4 | 11.4 | 100.0 |
|  | Total | 123 | 100.0 | 100.0 |  |

| **Q8** | | | | | |
| --- | --- | --- | --- | --- | --- |
|  | | Frequency | Percent | Valid Percent | Cumulative Percent |
| Valid | Never | 80 | 65.0 | 65.0 | 65.0 |
|  | Rarely | 6 | 4.9 | 4.9 | 69.9 |
|  | Sometimes | 19 | 15.4 | 15.4 | 85.4 |
|  | Frequently | 3 | 2.4 | 2.4 | 87.8 |
|  | Nearly always | 15 | 12.2 | 12.2 | 100.0 |
|  | Total | 123 | 100.0 | 100.0 |  |

| **Q9** | | | | | |
| --- | --- | --- | --- | --- | --- |
|  | | Frequency | Percent | Valid Percent | Cumulative Percent |
| Valid | Never | 86 | 69.9 | 69.9 | 69.9 |
|  | Rarely | 5 | 4.1 | 4.1 | 74.0 |
|  | Sometimes | 21 | 17.1 | 17.1 | 91.1 |
|  | Frequently | 2 | 1.6 | 1.6 | 92.7 |
|  | Nearly always | 9 | 7.3 | 7.3 | 100.0 |
|  | Total | 123 | 100.0 | 100.0 |  |

| **Q10** | | | | | |
| --- | --- | --- | --- | --- | --- |
|  | | Frequency | Percent | Valid Percent | Cumulative Percent |
| Valid | Never | 110 | 89.4 | 89.4 | 89.4 |
|  | Rarely | 1 | .8 | .8 | 90.2 |
|  | Sometimes | 3 | 2.4 | 2.4 | 92.7 |
|  | Frequently | 1 | .8 | .8 | 93.5 |
|  | Nearly always | 8 | 6.5 | 6.5 | 100.0 |
|  | Total | 123 | 100.0 | 100.0 |  |

| **Q11** | | | | | |
| --- | --- | --- | --- | --- | --- |
|  | | Frequency | Percent | Valid Percent | Cumulative Percent |
| Valid | Never | 16 | 13.0 | 13.0 | 13.0 |
|  | Rarely | 1 | .8 | .8 | 13.8 |
|  | Sometimes | 7 | 5.7 | 5.7 | 19.5 |
|  | Frequently | 3 | 2.4 | 2.4 | 22.0 |
|  | Nearly always | 96 | 78.0 | 78.0 | 100.0 |
|  | Total | 123 | 100.0 | 100.0 |  |

| **Q12** | | | | | |
| --- | --- | --- | --- | --- | --- |
|  | | Frequency | Percent | Valid Percent | Cumulative Percent |
| Valid | Never | 49 | 39.8 | 39.8 | 39.8 |
|  | Rarely | 1 | .8 | .8 | 40.7 |
|  | Sometimes | 2 | 1.6 | 1.6 | 42.3 |
|  | Nearly always | 71 | 57.7 | 57.7 | 100.0 |
|  | Total | 123 | 100.0 | 100.0 |  |

| **Physical** | | | | | |
| --- | --- | --- | --- | --- | --- |
|  | | Frequency | Percent | Valid Percent | Cumulative Percent |
| Valid | No | 95 | 77.2 | 77.2 | 77.2 |
|  | Yes | 28 | 22.8 | 22.8 | 100.0 |
|  | Total | 123 | 100.0 | 100.0 |  |

| **Social** | | | | | |
| --- | --- | --- | --- | --- | --- |
|  | | Frequency | Percent | Valid Percent | Cumulative Percent |
| Valid | No | 98 | 79.7 | 79.7 | 79.7 |
|  | Yes | 25 | 20.3 | 20.3 | 100.0 |
|  | Total | 123 | 100.0 | 100.0 |  |

| **Financial** | | | | | |
| --- | --- | --- | --- | --- | --- |
|  | | Frequency | Percent | Valid Percent | Cumulative Percent |
| Valid | No | 56 | 45.5 | 45.5 | 45.5 |
|  | Yes | 67 | 54.5 | 54.5 | 100.0 |
|  | Total | 123 | 100.0 | 100.0 |  |

| **Psychological** | | | | | |
| --- | --- | --- | --- | --- | --- |
|  | | Frequency | Percent | Valid Percent | Cumulative Percent |
| Valid | No | 85 | 69.1 | 69.1 | 69.1 |
|  | Yes | 38 | 30.9 | 30.9 | 100.0 |
|  | Total | 123 | 100.0 | 100.0 |  |

NEW FILE.

DATASET NAME DataSet1 WINDOW=FRONT.

GET

FILE="C:\Users\X415\Documents\Data analysis\Weaam's Data.sav".

DATASET NAME DataSet2 WINDOW=FRONT.

FREQUENCIES VARIABLES=School

/ORDER=ANALYSIS.

**Frequencies**

| **Notes** | | |
| --- | --- | --- |
| Output Created | | 25-DEC-2024 21:59:35 |
| Comments | |  |
| Input | Data | C:\Users\X415\Documents\Data analysis\Weaam's Data.sav |
|  | Active Dataset | DataSet2 |
|  | Filter | <none> |
|  | Weight | <none> |
|  | Split File | <none> |
|  | N of Rows in Working Data File | 985 |
| Missing Value Handling | Definition of Missing | User-defined missing values are treated as missing. |
|  | Cases Used | Statistics are based on all cases with valid data. |
| Syntax | | FREQUENCIES VARIABLES=School  /ORDER=ANALYSIS. |
| Resources | Processor Time | 00:00:00.00 |
|  | Elapsed Time | 00:00:00.01 |

[DataSet2] C:\Users\X415\Documents\Data analysis\Weaam's Data.sav

| **Statistics** | | |
| --- | --- | --- |
| School | | |
| N | Valid | 123 |
|  | Missing | 862 |

| **School** | | | | | |
| --- | --- | --- | --- | --- | --- |
|  | | Frequency | Percent | Valid Percent | Cumulative Percent |
| Valid | Regularly | 40 | 4.1 | 32.5 | 32.5 |
|  | Irregularly | 13 | 1.3 | 10.6 | 43.1 |
|  | Never | 70 | 7.1 | 56.9 | 100.0 |
|  | Total | 123 | 12.5 | 100.0 |  |
| Missing | System | 862 | 87.5 |  |  |
| Total | | 985 | 100.0 |  |  |

USE ALL.

COMPUTE filter_$=(VAR00002 = 1).

VARIABLE LABELS filter_$ 'VAR00002 = 1 (FILTER)'.

VALUE LABELS filter_$ 0 'Not Selected' 1 'Selected'.

FORMATS filter_$ (f1.0).

FILTER BY filter_$.

EXECUTE.

FREQUENCIES VARIABLES=School

/ORDER=ANALYSIS.

**Frequencies**

| **Notes** | | |
| --- | --- | --- |
| Output Created | | 25-DEC-2024 22:00:42 |
| Comments | |  |
| Input | Data | C:\Users\X415\Documents\Data analysis\Weaam's Data.sav |
|  | Active Dataset | DataSet2 |
|  | Filter | VAR00002 = 1 (FILTER) |
|  | Weight | <none> |
|  | Split File | <none> |
|  | N of Rows in Working Data File | 123 |
| Missing Value Handling | Definition of Missing | User-defined missing values are treated as missing. |
|  | Cases Used | Statistics are based on all cases with valid data. |
| Syntax | | FREQUENCIES VARIABLES=School  /ORDER=ANALYSIS. |
| Resources | Processor Time | 00:00:00.00 |
|  | Elapsed Time | 00:00:00.01 |

[DataSet2] C:\Users\X415\Documents\Data analysis\Weaam's Data.sav

| **Statistics** | | |
| --- | --- | --- |
| School | | |
| N | Valid | 123 |
|  | Missing | 0 |

| **School** | | | | | |
| --- | --- | --- | --- | --- | --- |
|  | | Frequency | Percent | Valid Percent | Cumulative Percent |
| Valid | Regularly | 40 | 32.5 | 32.5 | 32.5 |
|  | Irregularly | 13 | 10.6 | 10.6 | 43.1 |
|  | Never | 70 | 56.9 | 56.9 | 100.0 |
|  | Total | 123 | 100.0 | 100.0 |  |

NPAR TESTS

/K-W=TotalZarit BY School(1 3)

/MEDIAN=TotalZarit BY School(1 3)

/MISSING ANALYSIS.

**NPar Tests**

| **Notes** | | |
| --- | --- | --- |
| Output Created | | 25-DEC-2024 22:01:56 |
| Comments | |  |
| Input | Data | C:\Users\X415\Documents\Data analysis\Weaam's Data.sav |
|  | Active Dataset | DataSet2 |
|  | Filter | VAR00002 = 1 (FILTER) |
|  | Weight | <none> |
|  | Split File | <none> |
|  | N of Rows in Working Data File | 123 |
| Missing Value Handling | Definition of Missing | User-defined missing values are treated as missing. |
|  | Cases Used | Statistics for each test are based on all cases with valid data for the variable(s) used in that test. |
| Syntax | | NPAR TESTS  /K-W=TotalZarit BY School(1 3)  /MEDIAN=TotalZarit BY School(1 3)  /MISSING ANALYSIS. |
| Resources | Processor Time | 00:00:00.00 |
|  | Elapsed Time | 00:00:00.01 |
|  | Number of Cases Allowed^a^ | 112347 |

| a. Based on availability of workspace memory. |
| --- |

[DataSet2] C:\Users\X415\Documents\Data analysis\Weaam's Data.sav

**Kruskal-Wallis Test**

| **Ranks** | | | |
| --- | --- | --- | --- |
|  | School | N | Mean Rank |
| Total Zarit | Regularly | 40 | 56.55 |
|  | Irregularly | 13 | 73.04 |
|  | Never | 70 | 63.06 |
|  | Total | 123 |  |

| **Test Statistics^a,b^** | |
| --- | --- |
|  | Total Zarit |
| Chi-Square | 2.258 |
| df | 2 |
| Asymp. Sig. | .323 |

| a. Kruskal Wallis Test |
| --- |
| b. Grouping Variable: School |

**Median Test**

| **Frequencies** | | | | |
| --- | --- | --- | --- | --- |
|  | | School | | |
|  |  | Regularly | Irregularly | Never |
| Total Zarit | > Median | 15 | 8 | 33 |
|  | <= Median | 25 | 5 | 37 |

| **Test Statistics^a^** | |
| --- | --- |
|  | Total Zarit |
| N | 123 |
| Median | 12.00 |
| Chi-Square | 2.457^b^ |
| df | 2 |
| Asymp. Sig. | .293 |

| a. Grouping Variable: School |
| --- |
| b. 0 cells (.0%) have expected frequencies less than 5. The minimum expected cell frequency is 5.9. |

DATASET ACTIVATE DataSet2.

DATASET CLOSE DataSet1.

* Custom Tables.

CTABLES

/VLABELS VARIABLES=School TotalZarit DISPLAY=LABEL

/TABLE School BY TotalZarit [MEDIAN, PTILE 25, PTILE 75]

/CATEGORIES VARIABLES=School ORDER=A KEY=VALUE EMPTY=INCLUDE.

**Custom Tables**

| **Notes** | | |
| --- | --- | --- |
| Output Created | | 25-DEC-2024 22:11:01 |
| Comments | |  |
| Input | Data | C:\Users\X415\Documents\Data analysis\Weaam's Data.sav |
|  | Active Dataset | DataSet2 |
|  | Filter | VAR00002 = 1 (FILTER) |
|  | Weight | <none> |
|  | Split File | <none> |
|  | N of Rows in Working Data File | 123 |
| Syntax | | CTABLES  /VLABELS VARIABLES=School TotalZarit DISPLAY=LABEL  /TABLE School BY TotalZarit [MEDIAN, PTILE 25, PTILE 75]  /CATEGORIES VARIABLES=School ORDER=A KEY=VALUE EMPTY=INCLUDE. |
| Resources | Processor Time | 00:00:00.00 |
|  | Elapsed Time | 00:00:00.01 |

[DataSet2] C:\Users\X415\Documents\Data analysis\Weaam's Data.sav

| **Table 1** | | | | |
| --- | --- | --- | --- | --- |
|  | | Total Zarit | | |
|  |  | Median | Percentile 25 | Percentile 75 |
| School | Regularly | 10 | 8 | 17 |
|  | Irregularly | 14 | 12 | 20 |
|  | Never | 12 | 8 | 20 |
